# Cellular stressors may alter islet hormone cell proportions by moderation of alternative splicing patterns

**DOI:** 10.1093/hmg/ddz094

**Published:** 2019-05-17

**Authors:** Nicola Jeffery, Sarah Richardson, David Chambers, Noel G Morgan, Lorna W Harries

**Affiliations:** 1 Institute of Biomedical and Clinical Sciences, University of Exeter Medical School, Barrack Road, Exeter EX2 5DW, UK; 2 Wolfson Centre for Age-Related Diseases, King’s College London, London WC2R 2LS, UK

## Abstract

Changes to islet cell identity in response to type 2 diabetes (T2D) have been reported in rodent models, but are less well characterized in humans. We assessed the effects of aspects of the diabetic microenvironment on hormone staining, total gene expression, splicing regulation and the alternative splicing patterns of key genes in EndoC-βH1 human beta cells. Genes encoding islet hormones [somatostatin (*SST*), insulin (INS), *Glucagon (GCG)*], differentiation markers [Forkhead box O1 (*FOXO1*), Paired box 6, SRY box 9, NK6 Homeobox 1, NK6 Homeobox 2] and cell stress markers (DNA damage inducible transcript 3, *FOXO1*) were dysregulated in stressed EndoC-βH1 cells, as were some serine arginine rich splicing factor splicing activator and heterogeneous ribonucleoprotein particle inhibitor genes. Whole transcriptome analysis of primary T2D islets and matched controls demonstrated dysregulated splicing for ~25% of splicing events, of which genes themselves involved in messenger ribonucleic acid processing and regulation of gene expression comprised the largest group. Approximately 5% of EndoC-βH1 cells exposed to these factors gained SST positivity *in vitro*. An increased area of SST staining was also observed *ex vivo* in pancreas sections recovered at autopsy from donors with type 1 diabetes (T1D) or T2D (9.3% for T1D and 3% for T2D, respectively compared with 1% in controls). Removal of the stressful stimulus or treatment with the AKT Serine/Threonine kinase inhibitor SH-6 restored splicing factor expression and reversed both hormone staining effects and patterns of gene expression. This suggests that reversible changes in hormone expression may occur during exposure to diabetomimetic cellular stressors, which may be mediated by changes in splicing regulation.

## Introduction

A reduction in beta cell mass occurs during the progression of type 2 diabetes (T2D) and has been attributed to net enhancement of the rate of beta cell death (1,2). It is increasingly apparent, however, that changes in the differentiation status of beta cells may also be a contributory factor (3,4). Studies in mouse models of diabetes have described a gradual process of transdifferentiation from beta cells to alpha (5,6), and dedifferentiation to earlier progenitor cell types has also been reported (4,7). Beta to delta cell transdifferentiation has also been reported by lineage tracing in mouse islets in response to immunological stimuli (8). Data from human pancreas are scarce, but the limited information available suggests that similar changes in differentiation status may also occur in humans (9,10).

**Figure 1:**
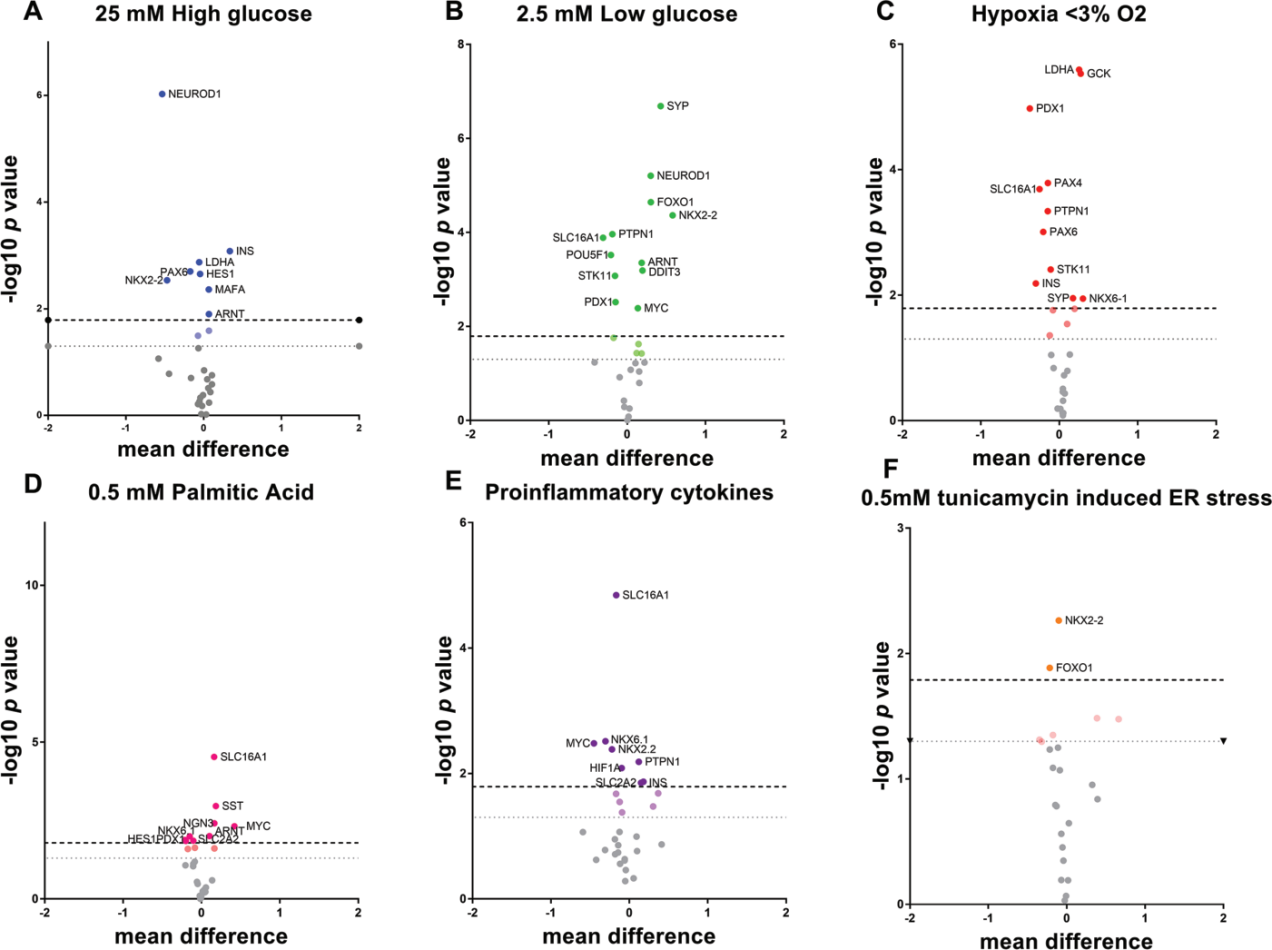
Effects of cell stresses on total gene expression of genes with roles in beta cell differentiation or function. Volcano plots demonstrating the most dysregulated genes in response to 24 h treatment with cellular stressors associated with a T2D microenvironment. Volcano plots are illustrative of the numbers of genes dysregulated by cell stresses from qRTPCR data. The *y* axis shows −log10 of *P*-value; *x* axis shows differences in mean logged relative quantification units between treatments and controls. Bonferroni corrections were performed to take account of the number of time points but not for the number of genes in the target panel as these are a priori. Upper line infers the Bonferroni multiple testing limit. The lower dotted line gives nominal significance. **(A)** 25 mM high glucose; **(B)** 2.5 mM low glucose; **(C)** Hypoxia < 3% O2; **(D)** 0.5 mM PI; **(E)** Cytokines TNFα, IL1β, INFγ; **(F)** 0.5 mM Tunicamycin for ER stress.

**Figure 2:**
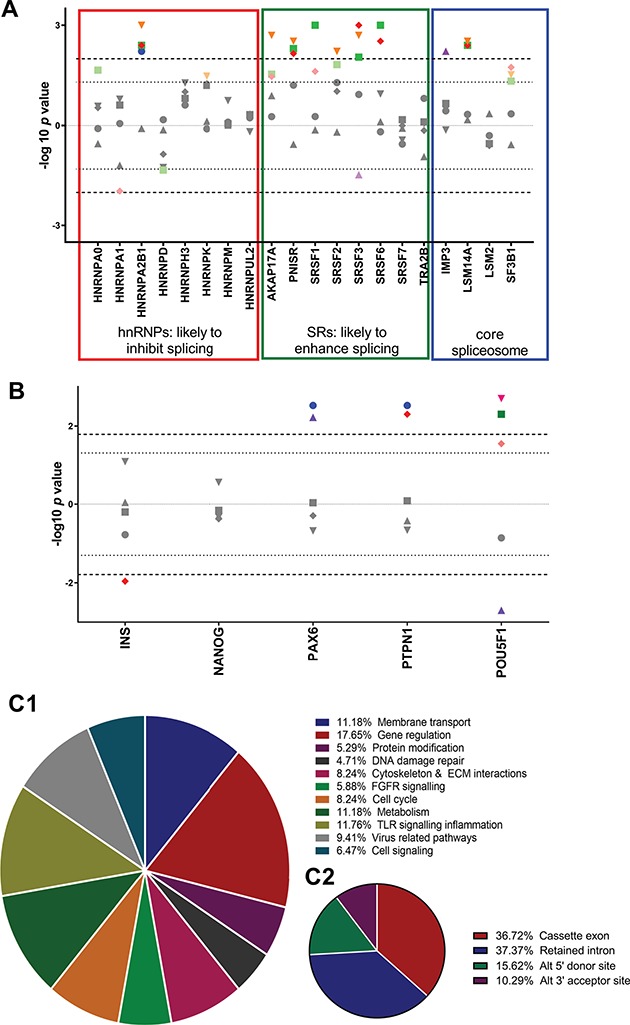
**(A)** Effects of cell stresses on splicing factor expression. Modified Manhattan-style plot corrected for direction of effect showing the effects of cellular stressors on splicing factor expression. The *y* axis shows −log10 of *P*-value with direction of effect from data generated by TLDA cards. Bonferroni corrections were performed to take account of the number of treatments but not for the splicing regulators as these are known to be affected by cell stresses in a number of other cell types (16). Upper line infers the Bonferroni multiple testing limit. The lower dotted line gives nominal significance. Blue circles 25 mM high glucose. Green squares, 2.5 mM low glucose; Red diamonds, < 3% O2; Purple triangles, 0.5 mM PI; Orange triangles, cytokines. Upper line infers the Bonferroni multiple testing limit. The lower dotted line gives nominal significance. **(B)** Effects of cell insult treatments on alternatively spliced gene expression. Modified Manhattan-style plot corrected for direction of effect. The *x* axis refs to the ratio of alternatively expressed isoforms at each locus from data generated by qRTPCR. Ratio was calculated by dividing expression of variants by expression of canonical transcripts. The *y* axis shows −log10 of *P*-value with direction of effect. Bonferroni corrections were performed to take account of the number of time points but not for the genes in the target panel as these are a priori. Upper line infers the Bonferroni multiple testing limit. The lower dotted line gives nominal significance. Blue circles, 25 mM glucose; green squares, 2.5 mM glucose; red diamonds, < 3% O2; purple triangles, 0.5 mM PI; pink inverse triangles, cytokines; orange hexagons, 0.5 mM. **(C)** The proportion of alternatively spliced genes demonstrating dysregulation in islets from donors with T2D. Data generated by Clariom D pico array. **(C1)** Pie chart showing the cellular and molecular functions of alternatively spliced transcripts that display dysregulated splicing patterns in islets from donors with T2D compared with matched controls. **(C2)** Pie chart showing the nature of splicing events that are dysregulated in islets from donors with T2D compared with matched controls.

**Figure 3:**
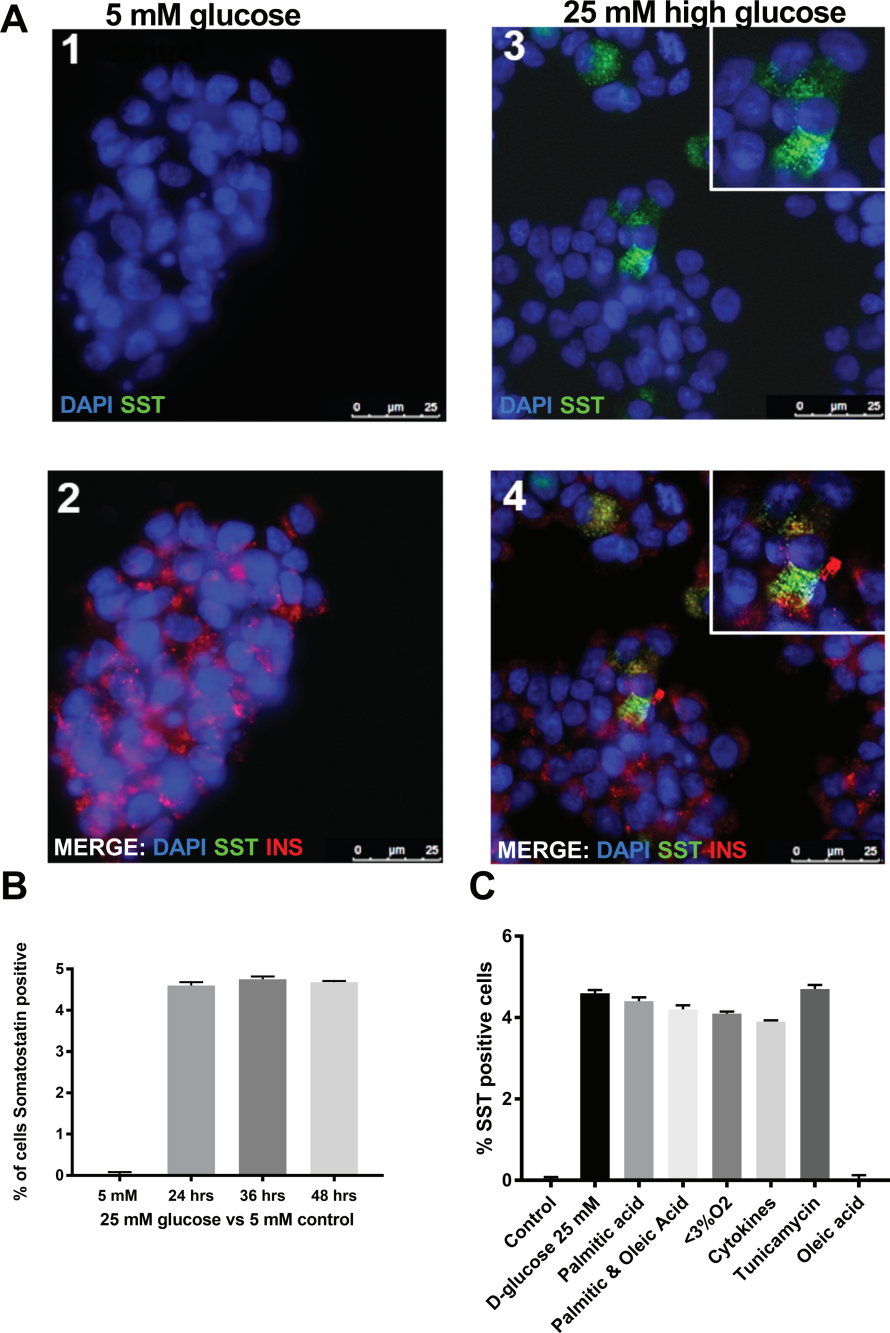
The effects of cell stressors to hormone expression in human beta cell line EndoC-βH1. **(A)** Immunofluorescence cytochemistry showing gain of SST expression in cells treated with 25 mM high glucose. Cells were treated for 24, 36 and 48 h and showed gains of SST expression at all time points. There was no increase in the proportion of cells gaining SST expression at later time points. Representative image panels from 36 h time point. (A) Panels 1 and 2 refer to treatment with 5 mM glucose as a control. Panels 3 and 4 refer to treatment with 25 mM glucose to mimic hyperglycaemia. The identity of the antibody used to stain is indicated in each panel. Of those cells that gained SST positivity, 17% were dual hormone positive. There were accompanying changes in the expression of INS and *SST* (*P* = 0.035, mean difference 0.223 and *P* = 0.004 mean difference 0.211, respectively). **(B)** Timecourse of SST positivity in cultures of EndoC-βH1cells treated with high glucose (25 mM). **(C)** The number of SST-positive cells in cultures of EndoC-βH1 cells treated with high glucose (25 mM), PI 0.5 mM, Hypoxia (3% O_2_), Cytokines (TNFα [1000 U/mL], INFγ [750 U/mL] and IL1β [75 U/mL]), Tunicamycin (0.5 mM) or oleic acid (0.5 mM).

**Figure 4:**
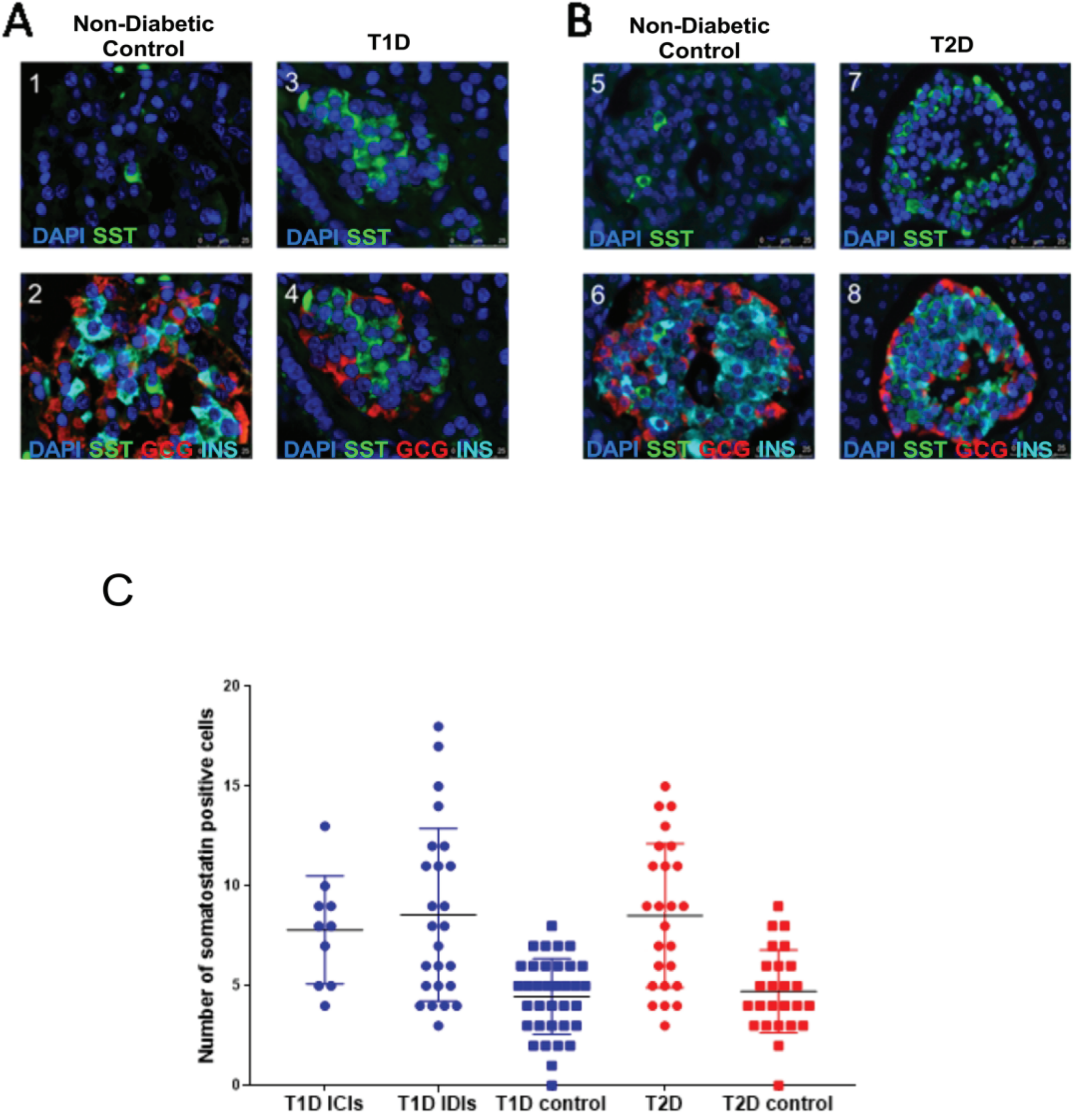
Hormone staining patterns of donor islets from controls or individuals with T2D. **(A)** Panels 1–4 are representative immunofluorescence images from human donor pancreatic tissue from controls (panels 1 and 2) and from cases of T1D compared to matched controls (panels 3 and 4). The identity of the antibody used to stain is indicated in each panel. **(B)** Panels 5–8 are representative immunofluorescence images from human donor pancreatic tissue from controls (panels 5 and 6) and from cases of T2D compared to matched controls (panels 7 and 8). **(C)** Graph showing interquartile range from delta cell counts for individuals without diabetes and patients with either T1D or T2D. We found a significant increase in the number of SST-positive cells in patients with either T1D INS-containing islets and INS-deficient islets (*P* = 3.0 × 10^−6^) or T2D (*P* = 2.20× 10^−4^) compared to their respective controls.

**Figure 5:**
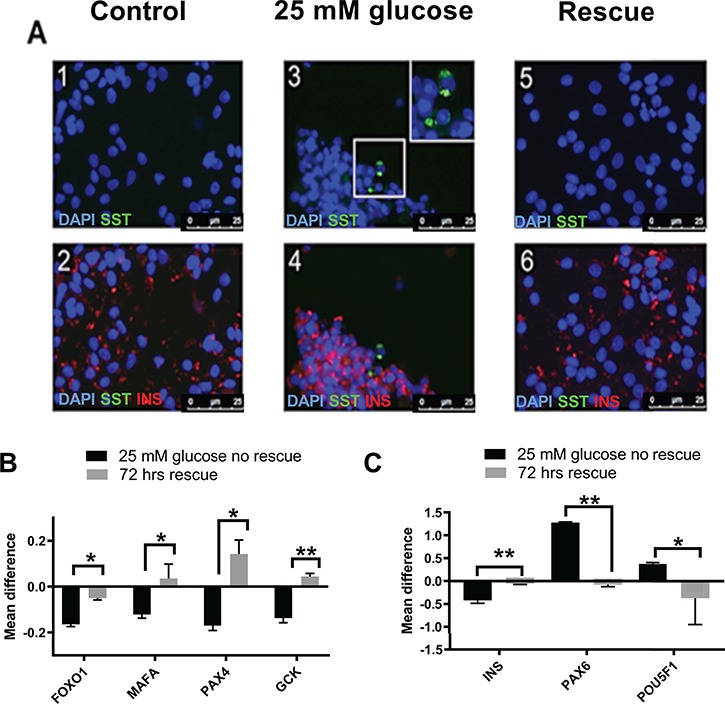
Rescue of phenotype by restoration to normal culture conditions. This figure illustrates the rescue of beta cell phenotype in EndoC-βH1 cells treated with 25 mM glucose for 24 h followed by restoration to 5 mM glucose culture conditions for 72 h. **(A)** Panels 1 and 2 are representative immunofluorescence images from untreated EndoC-βH1 cells. **(B)** Panels 3 and 4 are representative immunofluorescence images from EndoC-βH1 cells treated with 25 mM glucose for 24 h. **(C)** Panels 5 and 6 are representative immunofluorescence images from EndoC-βH1 cells treated with 25 mM glucose for 24 h but then restored to 5 mM glucose for 72 h. The identity of the antibody used to stain is indicated in each panel. Immunofluorescence image panel. **(B)** The graph gives the total gene expression levels, generated by qRTPCR, for genes previously demonstrating dysregulated expression in response to glucose under treated and rescued conditions. **(C)** The graph gives the isoform expression levels for genes previously demonstrating dysregulated splicing in response to glucose under treated and rescued conditions.

**Figure 6:**
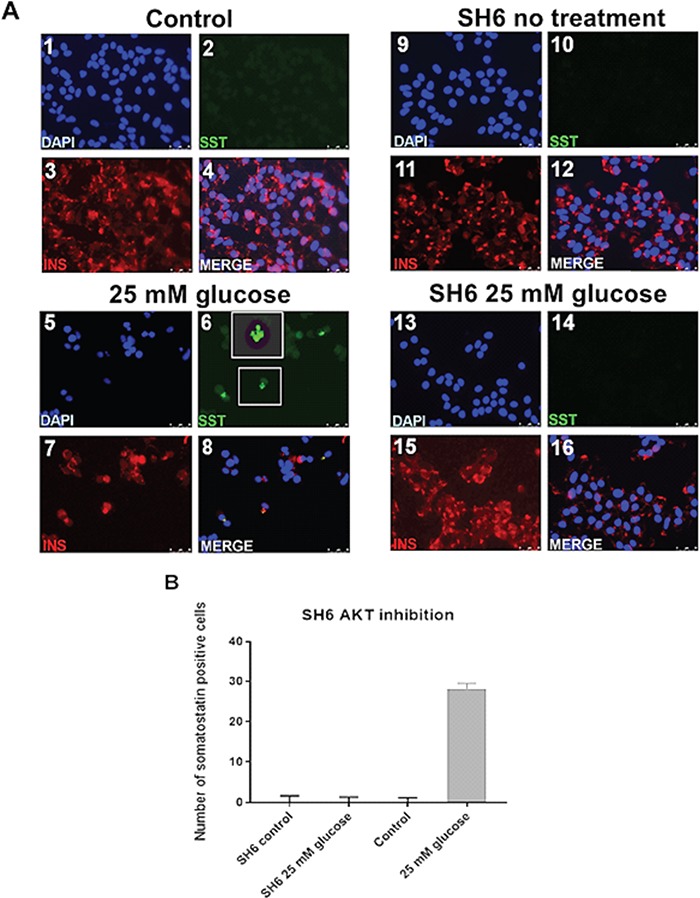
Hormone staining and gene expression changes following restoration of splicing factor expression using the AKT inhibitor SH-6. This figure illustrates the rescue of beta cell phenotype in EndoC-βH1 cells following restoration of splicing factor expression to that seen in normal glucose conditions. **(A)** Panels 1–4 are representative immunofluorescence images from untreated EndoC-βH1 cells. Panels 5–8 are representative immunofluorescence images from EndoC-βH1 cells treated with 25 mM glucose for 24 h. Panels 9–12 are representative Immunofluorescence images from EndoC-βH1 cells treated with SH-6 alone. Panels 13–16 are representative immunofluorescence images from EndoC-βH1 cells treated with 25 mM glucose and SH-6 for 24 h. The identity of the antibody used to stain is indicated in each panel. **(B)** Graph showing the number of SST-positive cells under different culture conditions.

Maintenance of beta-cell identity is determined by a tightly regulated transcriptional network, consisting of proteins encoded by the Pancreatic and Duodenal Homeobox 1 (*PDX1*), Forkhead Box O1 (*FOXO1*), NK2 Homeobox 2 (*NKX2-2*), MAF BZIP Transcription Factor A (*MAFA*), NK6 Homeobox 1 (*NKX6-1*) and Paired Box 6 (*PAX6*) genes amongst others. Most of these do not display specific expression in beta cells but are also expressed in other pancreatic islet cell types; their role in cell identity definition is likely to involve the relative balance of expression (5,11,12). Among these, the *FOXO1* gene, a downstream effector of AKT signalling in beta cells (13), has been demonstrated to play a specific role in the maintenance of beta cell differentiation status in mice (3). In addition to its role in regulation of beta cell plasticity (14) and in stress responses (15), FOXO1 has also been demonstrated to regulate alternative splicing (a powerful interface between cell identity and cell stress) by moderation of splicing factor expression in human primary fibroblasts (16).

The cellular microenvironment created by diabetes is stressful for beta cells (17), and elevated glucose levels have been linked to reduced expression of nodal genes within the transcriptional network that controls beta cell identity at the level of total gene expression (18). Changes to beta cell differentiation status also occur in response to chronic hyperglycaemia (7,19). Exposure of beta cells to the saturated fatty acid, palmitate or to pro-inflammatory cytokines has also been shown to induce widespread changes to the beta cell transcriptome (20,21). Altered beta cell identity may occur as a protectivemechanism in response to a stressful extracellular milieu, with cellular plasticity serving to protect beta cells which might, otherwise, be lost via apoptosis. As such, this reversible plasticity may allow for later re-differentiation should the extracellular environment become more conducive (7). Such effects could be relevant to all beta cells but they may be particularly important for ‘hub cells’ within islets, which are known to be more sensitive to insult than other beta cell subsets, resulting in beta cell failure (22,23). A recent study has also shown that beta cells are heterogeneous and can be ordered into three major states characterized by relative insulin (INS) expression and ER stress levels. High ER stress and low INS gene expression levels relate to a more immature beta cell state that doesn’t itself drive dedifferentiation but that may render them vulnerable to further insult (24).

We hypothesized that exposure to the cellular stressors that accompany the development of diabetes may cause disrupted regulation of key genes involved in the maintenance of beta cell identity, leading to changes in beta cell fate. We exposed human EndoC-βH1 beta cells in culture to a variety of diabetes-relevant cellular stressors and demonstrated alterations in the expression patterns of several key beta cell genes involved in the control of cell fate and cell identity and also in those controlling alternative splicing. Changes to the splicing patterns of 26% of genes were also apparent in human islets from donors with diabetes compared with non-diabetic controls. These changes were accompanied by alterations in hormone staining both *in vitro* and in *ex vivo*. These changes were reversible upon removal of the stressful stimulus or by moderation of splicing factor expression. Our observations suggest that altered splicing regulation and dysregulated splicing induced by cellular stress may influence the identity and proportions of hormone-expressing cells in human islets. Targeting splicing regulation may prove a fruitful avenue of investigation for future interventions to maintain beta cell differentiation in diabetes.

## Results

### Key beta cell fate and function genes show disrupted expression in response to cell stress stimuli in EndoC-**β**H1 cells

We tested the total expression levels of a panel of 31 preselected genes encoding hormones, determinants of beta cell identity or function and markers of cell stress in response to treatments designed to mimic aspects of diabetic pathophysiology. Large-scale changes in the expression of the majority of these genes were noted in response to all treatments across all time points (Supplementary Material, Table S1; Fig. 1A–F). Notable amongst these were *MAFA*, *PAX6*, *NEUROD1*, *NKX2-2*, *PDX1*, *NKX6-1* and *PAX4*, which are known to form an interacting network. All of these were responsive to either 25 mM D-glucose or 0.5 mM palmitic acid (PI). *PAX6*, *NKX2-2*, *PDX1*, *NKX6-1* and *PAX4* were similarly responsive to hypoxia. Differences in expression were also evident for *HES1*, SRY box 9, *NGN3 NANOG* and *POU5F1* genes, which are markers for cellular plasticity. Markers of cellular stress including DNA damage inducible transcript 3, *ARNT* and *MYC* also showed altered expression patterns under all conditions tested. Similar results were achieved following treatment with the generalized cellular stressor tunicamycin (Supplementary Material, Table S2).

#### Changes to the expression of splicing factors and beta cell splicing patterns in response to cell stress stimuli in EndoC-**β**H1 cells

We assessed the responses of genes encoding splicing factor genes, given their role in mediating the relationships between cell and environment (25,26). Several splicing factors, notably the genes encoding *HNRNPA2B1*, *AKAP17A*, *PNSIR*, serine arginine -rich splicing factor (*SRSF1*), *SRSF2*, *SRSF3*, *SRSF6* and *LSM14A* demonstrated dysregulated expression in response to treatment with cytokines, hypoxia, high/low glucose or altered lipids (Fig. 2A; Supplementary Material, Table S3). To determine whether these changes had functional significance, we also assessed the splicing patterns of key beta cell genes. Genes were included for analysis where they were known to produce alternatively spliced isoforms with functionally different roles in beta cell function or differentiation status that we could identify uniquely. A total of 3/5 genes fitting these criteria (*PAX6*, *PTPN1* and *POU5F1/OCT4)* demonstrated changes in the ratio of alternatively expressed isoforms in response to treatment (Fig. 2B; Supplementary Material, Table S4).

#### 26% Of alternative splicing events demonstrate dysregulation in human islets form donors with T2D

Assessment of transcriptome-wide patterns of alternative splicing demonstrated that of 47 944 splicing events detected in coding genes, 12 438 (26%) of splice sites in 6209 genes demonstrated altered usage (gene list available on request). Pathways analysis revealed that 6209 genes demonstrated altered splicing events in T2D islets, and these were clustered in 196 pathways that grouped into 11 cellular functions. Of these, gene regulation amounted to the largest share at 17.65% of all pathways (Fig. 2C1 and C2). Gene ontology (GO) analysis for biological processes and molecular function showed the top results were RNA metabolic process and RNA binding, respectively (Supplementary Material, Table S5).

#### Metabolic and ER stress is associated with gain of SST positivity in EndoC-**β**H1 cells

We quantified the effect of treatments designed to mimic the islet milieu in diabetes on cell identity in EndoC-βH1 cells cultured *in vitro*. A small (~5%) but consistent and statistically robust increase in the number of somatostatin (SST)-positive cells was noted in EndoC-βH1 cell cultures in response to incubation with elevated glucose levels (both D- and L-glucose) for 24 h. Control cells, incubated in 5.5 mM D-glucose, showed no evidence of SST immunopositivity (*P* = 9.1 × 10^−5^, Fig. 3A). The proportion of SST-positive cells remained stable over longer periods of culture, and no evidence of increased rates of cell death was seen in cells incubated under hyperglycaemic conditions (Fig. 3B). An increase in SST-positive cells was also noted in response to PI, hypoxia, pro-inflammatory cytokines and tunicamycin (*P* = 5.2 × 10^−5^, *P* = 4.2 × 10^−5^, *P* = 4.0 × 10^−3^ and *P* = 5.0 × 10^−3^, respectively; Fig. 3C). Conversely, no such increase was seen in response to oleic acid (*P* = 0.343), although cells exposed to both PI and oleic acids concurrently responded in a similar manner to those treated with palmitate alone (*P* = 0.003). No evidence of increased glucagon (CGC) expression was noted in control or treated EndoC-βH1 cells at the protein level although a rise in *GCG* messenger ribonucleic acid (mRNA) (and that encoding the transcription factor ARX) was evident in response to some treatment conditions. No change in the expression of *HHEX* mRNA transcripts was noted.

#### Pancreatic sections from donors with T1D or T2D demonstrate increased numbers of SST-positive cells

To determine if the effects we had noted in EndoC-βH1 cells *in vitro* were also recapitulated in human pancreas, we determined the proportion of different endocrine cell types in islet preparations from donors with long duration type 1 diabetes (T1D) or T2D and in control islets. We identified a significant increase in SST-positive (delta) cell area in tissue sections from patients with either long duration T1D or T2D compared to control islets. The total SST-positive area in *in situ* islets from T1D or T2D donors compared to control islets was 17069.9 μM (3%) v/s 6166.24 μM (1%) for T2D (*P* = 2.47 × 10^−9^) and 31 713 μM (9.3%) v/s 7360 μM (1% of islet area) for T1D (*P* = 2.1 × 10^−6^) (Fig. 4A and B; *n* = 10 images for 5 cases and 5 controls). An assessment using direct counting of cells rather than staining area also showed significant increases in the number of delta cells (Fig. 4C). There was no increase in the total islet cell count. Pancreatic islets from donors with long duration T1D contained a median number of 8 delta cells out of a total cell count of 62 (IQR = 6) compared with 4.5 delta cells (IQR = 3) out of a total cell count of 67 for age and sex-matched controls (*P* = 3.0 × 10^−6^). Similarly, pancreatic islets from donors with long duration T2D contained a median number of 8.5 delta cells (IQR = 6) out of a total cell count of 77 (IQR = 6) compared with 5 delta cells (IQR = 3) out of a total cell count of 71 for age and sex-matched controls (*P* = 2.2 × 10^−4^; Supplementary Material, Table S6).

#### Changes in hormone staining and beta cell expression patterns are reversible upon restoration of homeostatic conditions in EndoC-**β**H1 cells

To determine whether the changes in cell identity and gene expression patterns we have observed were permanent or reversible, we exposed EndoC-βH1 cells to cellular stressors for 24 h but then returned the cells to control conditions for 72 h prior to assessment. Following treatment with either high glucose or tunicamycin, we noted an increase in the number of SST-positive cells compared to controls (Fig. 5A; *P* = 3.69 × 10^−9^ and *P* = 1.3 × 10^−4^) and dysregulation of splicing ratios of candidate alternatively spliced genes (Fig. 5B and C; Supplementary Material, Table S7). Following return to control conditions, the number of SST-positive cells was indistinguishable from zero time point controls (Fig. 5A; *P* = 0.568 and *P* = 0.643), and patterns of alternative splicing were restored (Fig. 5B and C; Supplementary Material, Table S7) indicating that the treatment induced changes we noted were reversible.

#### The AKT inhibitor SH-6 restores splicing factor expression and abolishes SST positivity in EndoC BH1 cells

The AKT pathway has previously been suggested to have a role in regulation of splicing and splicing factor activity (26). There was no emergence of SST-positive staining in cells treated with low concentration of the AKT inhibitor SH-6 alone, whereas cells treated with 25 mM glucose demonstrated an increase in SST positivity similar to that we observed previously (*P* = 0.0004). EndoC-βH1 cells treated with SH-6 and subsequently exposed to 25 mM glucose for 24 h, however, showed no increase in the number of SST-positive cells compared to 5 mM glucose controls (Fig. 6A and B). This effect was also noted at the level of splicing factor expression and alternative splicing (Supplementary Material, Table S8).

## Discussion

In other cell types, alternative splicing forms a key part of the cellular defence against stress by adjusting the transcriptomic output of the cell to meet its new requirements (27). The cellular microenvironment induced by diabetes is stressful to beta cells, which may respond by either undergoing apoptosis (1,2) or by altering their differentiation state (10,28,29) resulting in an overall loss of beta cell mass. We hypothesized that exposure to diabetes-related cellular stresses may disrupt the normal patterns of splicing occurring in beta cells, potentially leading to changes in beta cell fate or function. We report here that human EndoC-βH1 beta cells treated with diabetes-related cellular stressors, such as altered glucose, fatty acids, cytokines or hypoxia or with the ER stress inducer tunicamycin, displayed altered expression of genes involved in splicing regulation, as well as altered expression of important genes for cell fate or function *in vitro*. Furthermore, islet samples from patients with T2D diabetes displayed large-scale disruption to transcriptome-wide splicing patterns, with over a quarter of genes demonstrating splicing alterations. The main class of genes with altered splicing comprised genes themselves involved in mRNA processing and regulation of gene expression. These transcriptomic changes were accompanied by an increase in the number of SST-positive cells in cultured EndoC-βH1 cells, an observation that was also evident in islet sections from human donors with either T1D or T2D. Similar effects were also noted when cells were exposed to L-glucose, suggesting that the response may occur in response to an osmotic stress and also that changes to beta cell differentiation status were not restricted to metabolic stress. The monounsaturated fatty acid oleate, a fatty acid that is not known to induce metabolic stress in human beta cells (30), elicited no response. The changes in cell hormone staining were reversible by either removal of the cellular stress or by resetting splicing factor expression to their previous level using small molecule moderators of splicing factor expression. These observations are consistent with previous reports that stress-related changes in beta cell differentiation status are modifiable once the noxious stimuli are removed (18).

Beta cell differentiation status is influenced by a transcriptional network of genes including *PAX6*, *PDX1*, *NEUROD1*, *NKX2-2*, *NKX6-1*, *MAFA*, *PAX4* and *FOXO1* (31,32), which demonstrated altered expression in our work. As the majority of these genes are also expressed in the other islet cell types, it is likely that beta cell maturity and identity is probably conferred by the subtle balance of these regulators. *PDX1* typically exhibits shared expression patterns across beta and delta cells; however, here we found reduced expression of *PDX1* with concomitant gain of *SST* gene and protein expression, which suggests they may not be fully committed to a delta cell phenotype (33) and thus not display all the expected delta cell markers. Similarly, there was also no significant gain in the expression of the delta cell marker *HHEX*, which is required for delta cell differentiation (34). Further, *FOXO1* is a key player in determination of beta cell fate and function; targeted disruption of this gene in mouse embryos is known to result in cell fate changes in developing mouse pancreas (13). It is also a pivotal part of the beta cell stress response (5,35). *FOXO1* and *PDX1* have also been shown to be mutually exclusive in the nucleus (5). In recent years, a new role for FOXO1 has also been identified as a regulator of the genes involved in alternative splicing decisions in some human cell types in the context of cellular senescence (16). Targeted knockdown of the *FOXO1* gene, or the use of inhibitor SH-6 that regulates FOXO1 activity, was able to restore splicing factor expression to levels seen in younger cells and restore cells to a younger phenotype (16). Alternative splicing has been well documented as a response to cellular stress (27). FOXO1 thus forms an interface between cell stress, alternative splicing and cell fate decisions. We have demonstrated here that transcripts encoding the serine arginine rich and heterogeneous ribonucleoprotein particle (*HNRNP*) genes, which encode positive and negative regulators of alternative splicing, respectively, were dysregulated in treated cells, as were patterns of alternative splicing for a limited number of functionally relevant candidate genes tested. We also identified that at the transcriptome-wide level, 26% of all splicing events were altered in human primary islets from donors with T2D, indicating that this observation may also hold true *in vivo*. Interestingly, one of the predominant pathways demonstrating splicing dysregulation included those themselves involved in RNA metabolism and RNA processing. Although dysregulation of splicing regulators was not seen at the level of total gene expression as changes are likely cell type-specific and signal therefore diluted by the presence of other cell types, there were changes in the splicing patterns of these genes. This is particularly pertinent in that splicing factor genes are primarily themselves regulated by alternative splicing (36). Similar results have been previously reported for other diabetes-relevant tissues such as adipose tissue (37). In support of the theory that disrupted splicing may underpin the cell identity changes we see, restoration of splicing factor expression to levels consistent with control cells using the AKT inhibitor SH-6, which is known to influence splicing factor expression (16), abolished the emergence of the SST-positive cell population, even in the presence of high glucose. Although SH-6 has not previously been shown to inhibit AKT in EndoC-βH1 cells, it has been shown to do so in another beta cell line RINm5F (38).

There are several potential explanations for the emergence of an SST-positive population. Firstly, this could arise from proliferation of a population of delta-like cells or progenitor cells in the cultures, or in the primary islets. It is also possible that progenitor cells may be present in the culture but are pushing the cells to an INS-positive rather than SST-positive fate. However, the lack of SST-positive staining in the absence of cell stress in the untreated EndoC-βH1 cell population suggests that expansion of a progenitor or delta cell population is an unlikely explanation for the emergence of SST positivity. Similarly, immunofluorescence data from the islets of patients with T2D showed no increase in total cell counts, which suggests that increased numbers of SST-positive or INS-positive cells may not be the result of expansion of a small pool of progenitor cells. Another explanation is that a proportion of the beta cells may have undergone beta to delta transdifferentiation. This phenomenon has been reported previously in the mouse, where beta-to-alpha cell transitions have been documented (5,6). The factors underlying apparent species differences may arise because of differences in islet architecture, gene expression and glucose sensing between rodent and human (39), but may also reflect profound differences in mRNA processing between these species since only 30% of splicing events are conserved between mice and humans (40).

We acknowledge that our study has limitations. Firstly, the changes in cell identity were observed in only a small proportion of the total cell population. The reasons for this are unclear but appear to be unrelated to the time of incubation since the response occurred very quickly (within 24 h) and was then stable for several days in the continued presence of the stressor. One possibility is that the cultures are heterogeneous and that a small population of cells is more susceptible to stress-induced plasticity than the majority. It would be interesting to determine whether this putatively ‘plastic’ cell population shares features with cells such as those identified as ‘hubs’ in intact islets since these also display a greater degree of plasticity than other beta cell sub populations (41–44). While it may also be that the EndoC βH1 cells line is contaminated with progenitor cells, as discussed above, the data from the rescue experiments might suggest that this is not the case, given the absence of SST positivity during normal culture conditions.

We also acknowledge that we have not conducted an exhaustive survey of splicing changes in the treated cells. The heterogeneous nature of the treated cultures means that only the largest effects would have been revealed by transcriptome-wide analysis. To circumvent this, it would be necessary to sort the SST-positive cells from their unchanged neighbours. This is a technically demanding task in EndoC βH1, which does not grow well in low cell densities, and may be susceptible to shear stress (which may confound our findings). SST is also not a cell surface marker, rendering fluorescence-activated cell sorting problematic. Taking into account these challenges, we opted to query transcriptome-wide splicing changes related to the presence of T2D in primary human islets, where we demonstrate that dysregulated splicing is clearly present.

We propose that our data are consistent with a model in which the appearance of an SST-positive subpopulation can appear in response to the imposition of cellular stress both *in vitro* and *ex vivo*, and we postulate that this may reflect dysregulation of splicing factor genes with consequent changes to splicing patterns that may be mediated by altered activation of AKT signalling and changes to FOXO1 activity. This work is, to our knowledge, the first demonstration of the emergence of SST positivity in human beta cells that may be specifically associated with changes in the regulation of mRNA splicing. Without lineage tracing experiments, which require expansion in non-human lines and are not trivial when using human model systems (45), it is not possible to infer that these changes we observed are directly due to beta to delta transdifferentiation. However, the consequence of the changes we observed may, at the molecular level, result in the altered expression of key genes involved in cell fate decisions. Targeting splicing patterns for therapeutic benefit is now an enticing possibility with several approaches already in clinical trials (46). We have shown that differentiation status in beta cells can be influenced by targeting splicing decisions, and a deeper understanding of the specific points at which such interventions could be focused might confer benefits for diabetes therapy in the future.

## Materials and Methods

### Culture and treatment of EndoC-**β**H1 cells

EndoC-βH1 cells at Passage <25 were seeded in 24-well plates at a density of 6.0 × 10^5^ cells/ml and maintained according to a modified humanized culture protocol as previously described (47) for 72 h prior to treatment. For assessment of the effects of altered glycaemia, cells were treated with 2.5 mM and 25 mM D- or L-glucose for 24, 36 or 48 h. Controls were maintained in 5 mM glucose media. For assessment of the effects of oxygen levels, cells were grown in 1%, 3% or 21% O_2_ for a period of 24, 36 or 48 h. For assessment of the effects of altered lipids, cells were treated with 0.5 mM PI, 0.5 mM oleic acid or 0.5 mM PI/0.5 mM oleic acid for 12, 24 and 48 h. To determine the effects of pro-inflammatory cytokines, cells were treated with TNFα (1000 U/mL, INFγ (750 U/mL) and IL1β (75 U/mL) for 12, 24 and 36 h. For assessment of the effects of Endoplasmic reticulum (ER) stress, cells were treated with 0.5 mM Tunicamycin for 24 h. Each treatment was carried out in three biological replicates, along with vehicle-only controls. Where cells were being used for subsequent immunofluorescence experiments they were cultured on 12 mm diameter coverslips and treated according to the conditions described above before being fixed with 4% paraformaldehyde.

### Immunofluorescent characterization of EndoC-**β**H1 cells *in vitro*

The expression of mature beta cell markers, as well as islet cell hormones in EndoC-βH1 cells grown as described above, was assessed using co-immunofluorescent microscopy. Briefly, following treatment, cells were fixed with 4% paraformaldehyde for 15 min at 4°C. Primary antibodies to SST and GCG were diluted in phosphate buffered saline with 0.1 M Lysine, 10% donor calf serum, 0.02% sodium azide and 0.02% Triton X-100 (ADST), to permeabilize the cell membranes and incubated overnight at 4°C. Primary antibodies were visualized using species specific highly cross-absorbed secondary antibodies (Invitrogen, Paisley, UK) diluted in ADST at 1/400 and incubated for 1 h at room temperature. Sequential staining was then performed with an antisera raised against INS (Supplementary Material, Table S9) diluted in ADST for 1 h, followed by a goat anti guinea-pig Alexa Fluor 555 along with 4',6-diamidino-2-phenylindole (DAPI) (Sigma-Aldrich, Steinheim, Germany; 1 μg/ml) for 1 h. Slides were visualized using a Leica AF6000 microscope (Leica, Milton Keynes, UK) and processed using the standard LASX Leica software platform. Ten randomly selected images were taken for each of the three biological replicates. Details of all antibodies are provided in Supplementary Material, Table S9.

### Immunofluorescent characterization of human pancreatic tissue sections from patients with T1D or T2D

Immunofluorescence staining was used to determine the expression of islet cell hormones in formalin-fixed paraffin embedded pancreatic sections from individuals with long duration T1D, long duration T2D and individuals without diabetes from the Exeter Archival Diabetes Biobank [http://foulis.vub.ac.be/ or the network of Pancreatic Organ Donors with Diabetes (nPOD) https://www.jdrfnpod.org/] (Supplementary Material, Table S10). The majority of samples were taken from the pancreatic body, rather than the head or tail. All samples were studied with ethical approval (WoSREC4; 15/WS/0258). Following dewaxing and rehydration, pancreas sections were subjected to heat-induced epitope retrievalin 10 mmol/l citrate buffer (pH 6). The sections were then sequentially probed with antisera to SST, CGC and INS (Supplementary Material, Table S9) that were detected using species specific highly cross-absorbed secondary antibodies conjugated with fluorescent dyes (Invitrogen; Supplementary Material, Table S9). Cell nuclei were stained with DAPI. Slides were visualized using a Leica AF6000 microscope (Leica) and processed using the standard LASX Leica software platform. Quantification of pancreatic endocrine cells types within the islet was achieved firstly by assessing the SST staining positive area as a percentage of total islet area between T1D/T2D patients and controls as described by others (48). To account for differences between the total islet area of T1D or T2D cases and their respective controls, total SST-positive areas were normalized to the global geometric mean of all samples. Secondly, the effects of diabetes status on endocrine cell types were assessed by counting the number of delta cells compared to the total islet cell count. Ten randomly selected images from seven cases and controls were taken for T1D and from five cases and controls for T2D. Areas of SST, INS and CGC staining were selected and measured using Image J (49).

### RNA extraction, reverse transcription and assessment of total gene expression in EndoC-**β**H1 cells

EndoC-βH1 cells were washed in Dulbecco’s phosphate buffered saline before RNA extraction using TRI® reagent to harvest RNA (Sigma-Aldrich). RNA concentrations were adjusted to 100 ng/μL prior to reverse transcription. Synthesis of cDNA was carried out using Superscript® VILO™ cDNA synthesis kit (Thermo Fisher, Foster City, USA) according to manufacturer’s instructions. Gene expression levels were measured in treated and untreated EndoC-βH1 cells by quantitative real-time PCR (qRTPCR). A panel of 31 a priori target genes was selected on the basis that they had known roles in either beta cell fate or function, were markers of cell stress or were markers of other pancreatic endocrine cell types (Supplementary Material, Table S11). Endogenous control genes (*PPIA*, *UBC*, *HPRT1*, *GUSB*, *B2M* and *IDH3B*) were empirically selected for stability in response to treatment. qRTPCR reaction mixes included 2.5 μL Taqman® Universal PCR mastermix II (no AmpErase® UNG) (Thermo Fisher, Waltham, MA, USA), 1.75 μL dH2O, 0.5 μL cDNA and 0.25 μL Taqman® gene assay (Thermo Fisher) in a 5 μL reaction volume. Cycling conditions were 50°C for 2 min, 95°C for 10 min and 50 cycles of 15 s at 95°C for 30 s and 1 min at 60°C. Reactions were carried out in three biological replicates and three technical replicates. Assay identifiers are given in Supplementary Material, Table S11. The relative expression of each test transcript was determined by the comparative Ct approach. Expression levels were calculated relative to the geometric mean of the endogenous controls, but also to the global mean of expression across all transcripts tested (as one would with an array), which was empirically determined not to vary across test conditions to provide a robust baseline. Expression levels were normalized to the median level of expression seen in untreated EndoC-βH1 cell controls. Differences in gene expression levels between mock-treated and treated EndoC-βH1 cells were compared to the same time point controls for each condition and investigated for statistical significance by *t*-test carried out using SPSS version 23 (IBM, North Castle, NY, USA). Data were presented as means ±  standard error of mean differences (S.E.D.).

### Determination of splicing factor expression in EndoC-**β**H1 cells

Splice site usage and thus patterns of alternative splicing are determined by the relative balance of SRSFs (splice site activators) and heterogeneous nuclear ribonucleoprotein particles (splice site inhibitors) at each splice site (50). Splicing factor assay IDs are given in Supplementary Material, Table S12. The expression levels of 20 splicing activator or splicing inhibitor genes was quantified in EndoC-βH1 cells using Taqman® Low Density Array (TLDA) on the Quantstudio 12 K Flex system. Cycling conditions were 1 cycle each of 50°C for 2 min, 94.5°C for 10 min and then 40 cycles of 97°C for 30 s and 57.9°C for 1 min. The reaction mixes included 50 μL Taqman® Fast Universal PCR Mastermix (Life Technologies, Foster City, USA), 30 μL dH_2_O and 20 μL cDNA template. The 100 μL reaction mix was dispensed into the TLDA card chamber and centrifuged twice for 1 min at 1000 rpm to ensure the correct distribution of solution to each well and removal of bubbles. Transcript expression was assessed the by the comparative Ct approach relative to the endogenous control genes *IDH3B*, *GUSB* and *PPIA*, which were selected based upon empirical evidence for stability in response to treatment and normalized to transcript expression in the untreated cells.

### Assessment of alternative splicing in EndoC-**β**H1 cells

Of the panel of 31 genes selected for total gene expression, 17 were known to exhibit alternative splicing. Of these, 5 were chosen for further analysis in EndoC-βH1 cells according to three criteria: (i) unique, isoform-specific exon boundaries, (ii) known functional differences between the isoforms and (iii) relevance of isoform differences to beta cell differentiation status or islet function. Isoforms of the *INS*, *PAX6*, *POU5F1* (*OCT4*), *NANOG* and *PTPN1* genes fulfilled these criteria. Isoform-specific Taqman custom assays (Thermo Fisher) were designed across unique splice boundaries that distinguish between the functionally distinct isoforms. Probe and primer sequences are given in Supplementary Material, Table S13.

### Reversal of delta cell phenotype in EndoC-**β**H1 cells by restoration of homeostasis

To test for reversibility of effect in our system, cells were first treated with 25 mM glucose or 0.5 mM Tunicamycin for 24 h as described above. Immediately following this, cells were restored to normal culture conditions for 72 h prior to assessment of cell identity, total gene expression and splicing patterns.

### Reversal of delta cell phenotype in EndoC-**β**H1 cells by restoration of homeostasis or by small molecule inhibition of AKT pathway

Previous reports suggest that changes to beta cell identity can be reversed upon removal of cellular stress (15). Reports have suggested that the AKT pathway, one of the signalling mechanisms whereby cells can communicate signals from stimuli such as cellular stress stimuli from the exterior of the cell, is involved with regulation of splicing decisions and has also been linked with changes in beta cell plasticity (17,25,26). To test for reversibility of effect in our system, cells were first treated with 25 mM glucose or 0.5 mM Tunicamycin for 24 h as described above. Immediately following this, cells were restored to normal culture conditions for 72 h prior to assessment of cell identity, total gene expression and splicing patterns. To assess the effect of AKT inhibition on response to hyperglycaemia, cells were treated with 25 mM glucose alone, 1 μM of the AKT inhibitor SH-6 alone or 25 mM glucose in combination with 1 μM SH-6 for 24 h prior to assessment of splicing patterns and cell identity as described above.

### Whole transcriptome analysis of patterns of alternative splicing in islets from human donors with T2D compared with non-diabetic human islets

We assessed patterns of total gene expression and transcriptome-wide patterns of alternative splicing in an initial discovery set of six islets preparations from donors with T2D versus six non-diabetic samples from the integrated islet distribution programme using the ClariomD pico system (Thermo Fisher). A subsequent validation set of six islets preparations from donors with T2D versus six non-diabetic samples were also assessed to validate results from the first islets set. All islets were matched for age, gender and Body mass index (BMI). Sample preparation and gene chip cartridge arrays were performed by UK Bioinformatics Ltd (Woldingham, Caterham, Surrey). Transcriptome data for total gene and splicing expression were analysed using TAC 4.0.2.10 (Applied Biosystems, Thermo Fisher). For both total expression and splicing genes were filtered to remove non-coding RNAs, pseudogenes and incompletely annotated genes. Differentially expressed genes and dysregulated splicing events with a *P*-value of < 0.05 in both islets sets were taken forward into pathways and GO analysis carried out using Enrichr (51,52).

## Supplementary Material

Jeffery_et_al_2019_R1-clean_copy_ddz094

Supplemental_tables_ddz094

## References

[1] Butler P.C., Meier J.J., Butler A.E. and Bhushan A. (2007) The replication of beta cells in normal physiology, in disease and for therapy. Nat. Clin. Pract. Endocrinol. Metab., 3, 758–768. 17955017 10.1038/ncpendmet0647

[2] Marchetti P., Del Guerra S., Marselli L., Lupi R., Masini M., Pollera M., Bugliani M., Boggi U., Vistoli F., Mosca F. et al. (2004) Pancreatic islets from type 2 diabetic patients have functional defects and increased apoptosis that are ameliorated by metformin. J. Clin. Endocrinol. Metab., 89, 5535–5541. 15531508 10.1210/jc.2004-0150

[3] Talchai C., Xuan S., Lin H.V., Sussel L. and Accili D. (2012) Pancreatic β-cell dedifferentiation as mechanism of diabetic β-cell failure. Cell, 150, 1223–1234. 22980982 10.1016/j.cell.2012.07.029 PMC3445031

[4] van der Meulen T. and Huising M.O. (2015) The role of transcription factors in the transdifferentiation of pancreatic islet cells. J. Mol. Endocrinol., 54, R103–R117. 25791577 10.1530/JME-14-0290 PMC4373662

[5] Kitamura T. (2013) The role of FOXO1 in β-cell failure and type 2 diabetes mellitus. Nat. Rev. Endocrinol., 9, 615–623. 23959366 10.1038/nrendo.2013.157

[6] Gao T., McKenna B., Li C., Reichert M., Nguyen J., Singh T., Yang C., Pannikar A., Doliba N., Zhang T. et al. (2014) Pdx1 maintains β cell identity and function by repressing an α cell program. Cell Metab., 19, 259–271. 24506867 10.1016/j.cmet.2013.12.002 PMC3950964

[7] Puri S. and Hebrok M. (2012) Diabetic β cells: to be or not to be? Cell, 150, 1103–1104. 22980973 10.1016/j.cell.2012.08.021 PMC4692270

[8] Piran R., Lee S.H., Li C.R., Charbono A., Bradley L.M. and Levine F. (2014) Pharmacological induction of pancreatic islet cell transdifferentiation: relevance to type I diabetes. Cell Death Dis., 5, e1357. 25077543 10.1038/cddis.2014.311 PMC4123101

[9] White M.G., Marshall H.L., Rigby R., Huang G.C., Amer A., Booth T., White S. and Shaw J.A.M. (2013) Expression of mesenchymal and α-cell phenotypic markers in islet β-cells in recently diagnosed diabetes. Diabetes Care, 36, 3818–3820. 24062329 10.2337/dc13-0705 PMC3816907

[10] Spijker H.S., Ravelli R.B.G., Mommaas-Kienhuis A.M., van Apeldoorn A.A., Engelse M.A., Zaldumbide A., Bonner-Weir S., Rabelink T.J., Hoeben R.C., Clevers H. et al. (2013) Conversion of mature human β-cells into glucagon-producing α-cells. Diabetes, 62, 2471–2480. 23569174 10.2337/db12-1001 PMC3712074

[11] Schaffer A.E., Taylor B.L., Benthuysen J.R., Liu J., Thorel F., Yuan W., Jiao Y., Kaestner K.H., Herrera P.L., Magnuson M.A. et al. (2013) Nkx6.1 controls a gene regulatory network required for establishing and maintaining pancreatic Beta cell identity. PLoS Genet., 9, e1003274. 23382704 10.1371/journal.pgen.1003274 PMC3561089

[12] Guo S., Dai C., Guo M., Taylor B., Harmon J.S., Sander M., Robertson R.P., Powers A.C. and Stein R. (2013) Inactivation of specific beta cell transcription factors in type 2 diabetes. J. Clin. Invest., 123, 3305–3316. 23863625 10.1172/JCI65390 PMC3726150

[13] Al-Masri M., Krishnamurthy M., Li J., Fellows G.F., Dong H.H., Goodyer C.G. and Wang R. (2010) Effect of forkhead box O1 (FOXO1) on beta cell development in the human fetal pancreas. Diabetologia, 53, 699–711. 20033803 10.1007/s00125-009-1632-0

[14] Elghazi L., Weiss A.J., Barker D.J., Callaghan J., Staloch L., Sandgren E.P., Gannon M., Adsay V.N. and Bernal-Mizrachi E. (2009) Regulation of pancreas plasticity and malignant transformation by Akt Signaling. Gastroenterology, 136, 1091–1103 e1098. 19121634 10.1053/j.gastro.2008.11.043 PMC2739876

[15] Ponugoti B., Dong G. and Graves D.T. (2012) Role of forkhead transcription factors in diabetes-induced oxidative stress. Exp. Diabetes Res., 2012, 939751. 22454632 10.1155/2012/939751 PMC3290826

[16] Latorre E., Ostler E.O., Faragher R.G.A. and Harries L.W. (2018) FOXO1 and ETV6 genes may represent novel regulators of splicing factor expression in cellular senescence. FASEB J., 33, 1086–1097. 30088951 10.1096/fj.201801154R

[17] Li N., Frigerio F. and Maechler P. (2008) The sensitivity of pancreatic beta-cells to mitochondrial injuries triggered by lipotoxicity and oxidative stress. Biochem. Soc. Trans., 36, 930–934. 18793163 10.1042/BST0360930

[18] Swisa A., Avrahami D., Eden N., Zhang J., Feleke E., Dahan T., Cohen-Tayar Y., Stolovich-Rain M., Kaestner K.H., Glaser B. et al. (2017) PAX6 maintains β cell identity by repressing genes of alternative islet cell types. J. Clin. Invest., 127, 230–243. 27941241 10.1172/JCI88015 PMC5199694

[19] Brereton M.F., Iberl M., Shimomura K., Zhang Q., Adriaenssens A.E., Proks P., Spiliotis I.I., Dace W., Mattis K.K., Ramracheya R. et al. (2014) Reversible changes in pancreatic islet structure and function produced by elevated blood glucose. Nat. Commun., 5, 4639. 25145789 10.1038/ncomms5639 PMC4143961

[20] Cnop M., Abdulkarim B., Bottu G., Cunha D.A., Igoillo-Esteve M., Masini M., Turatsinze J.V., Griebel T., Villate O., Santin I. et al. (2014) RNA sequencing identifies dysregulation of the human pancreatic islet transcriptome by the saturated fatty acid palmitate. Diabetes, 63, 1978–1993. 24379348 10.2337/db13-1383

[21] Ortis F., Naamane N., Flamez D., Ladrière L., Moore F., Cunha D.A., Colli M.L., Thykjaer T., Thorsen K., Ørntoft T.F. et al. (2010) Cytokines interleukin-1β and tumor necrosis factor-α regulate different transcriptional and alternative splicing networks in primary β-cells. Diabetes, 59, 358–374. 19934004 10.2337/db09-1159 PMC2809955

[22] Dorrell C., Schug J., Canaday P.S., Russ H.A., Tarlow B.D., Grompe M.T., Horton T., Hebrok M., Streeter P.R., Kaestner K.H. et al. (2016) Human islets contain four distinct subtypes of β cells. Nat. Commun., 7, 11756. 27399229 10.1038/ncomms11756 PMC4942571

[23] Johnston N.R., Mitchell R.K., Haythorne E., Pessoa M.P., Semplici F., Ferrer J., Piemonti L., Marchetti P., Bugliani M., Bosco D. et al. Beta cell hubs dictate pancreatic islet responses to glucose. Cell Metab., 24, 389–401. 27452146 10.1016/j.cmet.2016.06.020 PMC5031557

[24] Xin Y., Dominguez Gutierrez G., Okamoto H., Kim J., Lee A.-H., Adler C., Ni M., Yancopoulos G.D., Murphy A.J. and Gromada J. (2018) Pseudotime ordering of single human β-cells reveals states of insulin production and unfolded protein response. Diabetes, 67, 1783–1794. 29950394 10.2337/db18-0365

[25] Biamonti G. and Caceres J.F. (2009) Cellular stress and RNA splicing. Trends Biochem. Sci., 34, 146–153. 19208481 10.1016/j.tibs.2008.11.004

[26] Long J.C. and Caceres J.F. (2009) The SR protein family of splicing factors: master regulators of gene expression. Biochem. J., 417, 15–27. 19061484 10.1042/BJ20081501

[27] Mastrangelo A.M., Marone D., Laido G., De Leonardis A.M. and De Vita P. (2012) Alternative splicing: enhancing ability to cope with stress via transcriptome plasticity. Plant Sci., 185–186, 40–49. 10.1016/j.plantsci.2011.09.006 22325865

[28] Wang Z., York N.W., Nichols C.G. and Remedi M.S. (2014) Pancreatic beta cell dedifferentiation in diabetes and redifferentiation following insulin therapy. Cell Metab., 19, 872–882. 24746806 10.1016/j.cmet.2014.03.010 PMC4067979

[29] Fujimoto K. and Polonsky K.S. (2009) Pdx1 and other factors that regulate pancreatic beta-cell survival. Diabetes Obes. Metab., 11(Suppl. 4), 30–37. 19817786 10.1111/j.1463-1326.2009.01121.x PMC2802270

[30] Welters H.J., Tadayyon M., Scarpello J.H., Smith S.A. and Morgan N.G. (2004) Mono-unsaturated fatty acids protect against beta-cell apoptosis induced by saturated fatty acids, serum withdrawal or cytokine exposure. FEBS Lett., 560, 103–108. 14988006 10.1016/S0014-5793(04)00079-1

[31] Gosmain Y., Katz L.S., Masson M.H., Cheyssac C., Poisson C. and Philippe J. (2012) Pax6 is crucial for β-cell function, insulin biosynthesis, and glucose-induced insulin secretion. Mol. Endocrinol., 26, 696–709. 22403172 10.1210/me.2011-1256 PMC5417143

[32] Mitchell R.K., Nguyen-Tu M.-S., Chabosseau P., Callingham R.M., Pullen T.J., Cheung R., Leclerc I., Hodson D.J. and Rutter G.A. (2017) The transcription factor Pax6 is required for pancreatic β cell identity, glucose-regulated ATP synthesis, and Ca2+ dynamics in adult mice. J. Biol. Chem., 292, 8892–8906. 28377501 10.1074/jbc.M117.784629 PMC5448123

[33] DiGruccio M.R., Mawla A.M., Donaldson C.J., Noguchi G.M., Vaughan J., Cowing-Zitron C., Meulen T. and Huising M.O. (2016) Comprehensive alpha, beta and delta cell transcriptomes reveal that ghrelin selectively activates delta cells and promotes somatostatin release from pancreatic islets. Mol Metab., 5, 449–458. 27408771 10.1016/j.molmet.2016.04.007 PMC4921781

[34] Zhang J., McKenna L.B., Bogue C.W. and Kaestner K.H. (2014) The diabetes gene Hhex maintains delta-cell differentiation and islet function. Genes Dev., 28, 829–834. 24736842 10.1101/gad.235499.113 PMC4003275

[35] Kobayashi M., Kikuchi O., Sasaki T., Kim H.-J., Yokota-Hashimoto H., Lee Y.-S., Amano K., Kitazumi T., Susanti V.Y., Kitamura Y.I. et al. (2012) FoxO1 as a double-edged sword in the pancreas: analysis of pancreas- and β-cell-specific FoxO1 knockout mice. Am. J. Physiol. Endocrinol. Metab., 302, E603–E613. 22215655 10.1152/ajpendo.00469.2011

[36] Lareau L. and E Brenner S. (2015) Regulation of splicing factors by alternative splicing and NMD is conserved between kingdoms yet evolutionarily flexible. Mol Biol Evol., 32, 1072–1079. 25576366 10.1093/molbev/msv002 PMC4379411

[37] Kaminska D., Kakela P., Nikkola E., Venesmaa S., Ilves I., Herzig K.H., Kolehmainen M., Karhunen L., Kuusisto J., Gylling H. et al. (2016) Regulation of alternative splicing in human obesity loci. Obesity (Silver Spring), 24, 2033–2037. 27515906 10.1002/oby.21587 PMC5215786

[38] Tejedo J.R., Cahuana G.M., Ramirez R., Esbert M., Jimenez J., Sobrino F. and Bedoya F.J. (2004) Nitric oxide triggers the phosphatidylinositol 3-kinase/Akt survival pathway in insulin-producing RINm5F cells by arousing Src to activate insulin receptor substrate-1. Endocrinology, 145, 2319–2327. 14764634 10.1210/en.2003-1489

[39] Benner C., Meulen T., Cacéres E., Tigyi K., Donaldson C.J., Huising M.O., Caceres E., Tigyi K., Donaldson C.J. and Huising M.O. (2014) The transcriptional landscape of mouse beta cells compared to human beta cells reveals notable species differences in long non-coding RNA and protein-coding gene expression. BMC Genomics, 15, 620. 25051960 10.1186/1471-2164-15-620 PMC4124169

[40] Barbosa-Morais N.L., Irimia M., Pan Q., Xiong H.Y., Gueroussov S., Lee L.J., Slobodeniuc V., Kutter C., Watt S., Colak R. et al. (2012) The evolutionary landscape of alternative splicing in vertebrate species. Science, 338, 1587–1593. 23258890 10.1126/science.1230612

[41] Szabat M., Lynn F.C., Hoffman B.G., Kieffer T.J., Allan D.W. and Johnson J.D. (2012) Maintenance of β-Cell Maturity and Plasticity in the Adult Pancreas. Dev. Biol. Concepts Adult Physiol., 61, 1365–1371. 10.2337/db11-1361 PMC3357305 22618775

[42] Szabat M., Piret J.M., Luciani D.S. and Johnson J.D. (2009) Maturation of adult β-cells revealed using a Pdx1/insulin dual-reporter lentivirus. Endocrinology, 150, 1627–1635. 19095744 10.1210/en.2008-1224

[43] Assouline-Thomas B.A., Austin E., Blaichman J., Kapeluto J., Moosavi M., Petropavlovskaia M., Hanley S.C. and Rosenberg L. (2010) β-cell mass dynamics and islet cell plasticity in human type 2 diabetes. Endocrinology, 151, 1462–1472. 20176718 10.1210/en.2009-1277

[44] Avrahami D., Klochendler A., Dor Y. and Glaser B. (2017) Beta cell heterogeneity: an evolving concept. Diabetologia, 60, 1363–1369. 28597073 10.1007/s00125-017-4326-z PMC5554543

[45] Furuyama K., Chera S., van Gurp L., Oropeza D., Ghila L., Damond N., Vethe H., Paulo J.A., Joosten A.M., Berney T. et al. (2019) Diabetes relief in mice by glucose-sensing insulin-secreting human α-cells. Nature, 567, 43–48. 30760930 10.1038/s41586-019-0942-8 PMC6624841

[46] Havens M.A., Duelli D.M. and Hastings M.L. (2013) Targeting RNA splicing for disease therapy. Wiley Interdiscip. Rev. RNA, 4, 247–266. 23512601 10.1002/wrna.1158 PMC3631270

[47] Jeffery N., Richardson S., Beall C. and Harries L.W. (2017) The species origin of the cellular microenvironment influences markers of beta cell fate and function in EndoC-betaH1 cells. Exp. Cell Res., 361, 284–291. 29107069 10.1016/j.yexcr.2017.10.028

[48] Plesner A., Ten Holder J.T. and Verchere C.B. (2014) Islet remodeling in female mice with spontaneous autoimmune and streptozotocin-induced diabetes. PLoS One, 9, e102843. 25101835 10.1371/journal.pone.0102843 PMC4125302

[49] Schindelin J., Arganda-Carreras I., Frise E., Kaynig V., Longair M., Pietzsch T., Preibisch S., Rueden C., Saalfeld S., Schmid B. et al. (2012) Fiji: an open-source platform for biological-image analysis. Nat. Methods, 9, 676–682. 22743772 10.1038/nmeth.2019 PMC3855844

[50] Cartegni L., Chew S.L. and Krainer A.R. (2002) Listening to silence and understanding nonsense: exonic mutations that affect splicing. Nat. Rev. Genet., 3, 285–298. 11967553 10.1038/nrg775

[51] Kuleshov M.V., Jones M.R., Rouillard A.D., Fernandez N.F., Duan Q., Wang Z., Koplev S., Jenkins S.L., Jagodnik K.M., Lachmann A. et al. (2016) Enrichr: a comprehensive gene set enrichment analysis web server 2016 update. Nucleic Acids Res., 44, W90–W97. 27141961 10.1093/nar/gkw377 PMC4987924

[52] Chen E.Y., Tan C.M., Kou Y., Duan Q., Wang Z., Meirelles G.V., Clark N.R. and Ma’ayan A. (2013) Enrichr: interactive and collaborative HTML5 gene list enrichment analysis tool. BMC Bioinformatics, 14, 128. 23586463 10.1186/1471-2105-14-128 PMC3637064

