# Supplementary material for: Cellular stressors may alter islet hormone cell proportions by moderation of alternative splicing patterns
Source: Hum Mol Genet. 2019 May 17;28(16):2763–74. doi: 10.1093/hmg/ddz094 (PMC6687954; doi:10.1093/hmg/ddz094)
Supplement: Jeffery_et_al_2019_R1-clean_copy_ddz094 [file jeffery_et_al_2019_r1-clean_copy_ddz094.doc]

# Cellular stressors may alter islet hormone cell proportions by moderation of alternative splicing patterns.

Nicola Jeffery^1^, Sarah Richardson^1^, David Chambers^2^, Noel G. Morgan^1^, Lorna W. Harries^1*^.

^1^*Institute of Biomedical and Clinical Sciences, University of Exeter Medical School, Barrack Road, Exeter, UK, EX2 5DW*

*^2^Wolfson Centre for Age Related Diseases, Kings College London, London, UK, WC2R 2LS*

***Corresponding author:***

*Professor Lorna W. Harries,*

*University of Exeter Medical School*

*Barrack Road,*

*Exeter, EX2 5DW.*

*Tel: 44-1392-406773*

*Email: L.W.Harries@exeter.ac.uk*

**Abstract**

Changes to islet cell identity in response to type 2 diabetes have been reported in rodent models, but are less well characterised in humans. We assessed the effects of aspects of the diabetic microenvironment on hormone staining, total gene expression, splicing regulation and the alternative splicing patterns of key genes in EndoC-βH1 human beta cells. Genes encoding islet hormones (*SST, INS, GCG*), differentiation markers (*FOXO1, PAX6, SOX9, NKX6.1, NKX6.2*) and cell stress markers (*DDIT3, FOXO1*) were dysregulated in stressed EndoC-βH1 cells, as were some *SRSF* splicing activator and *HNRNP* inhibitor genes. Whole transcriptome analysis of primary T2D islets and matched controls demonstrated dysregulated splicing for approximately 25% of splicing events, of which genes themselves involved in mRNA processing and regulation of gene expression comprised the largest group. Approximately 5% of EndoC-βH1 cells exposed to these factors gained somatostatin positivity in vitro. An increased area of somatostatin staining was also observed ex vivo in pancreas sections recovered at autopsy from donors with type 1 or type 2 diabetes (9.3% for T1D and 3% for T2D respectively compared with 1% in controls). Removal of the stressful stimulus or treatment with the AKT inhibitor SH-6 restored splicing factor expression and reversed both hormone staining effects and patterns of gene expression. This suggests that reversible changes in hormone expression may occur during exposure to diabetomimetic cellular stressors, which may be mediated by changes in splicing regulation.

**Introduction**

A reduction in beta cell mass occurs during the progression of Type 2 diabetes (T2D) and has been attributed to net enhancement of the rate of beta cell death (1, 2). It is increasingly apparent however that changes in the differentiation status of beta cells may also be a contributory factor (3, 4). Studies in mouse models of diabetes have described a gradual process of trans-differentiation from beta cells to alpha (5, 6), and dedifferentiation to earlier progenitor cell types has also been reported (4, 7). Beta to delta cell transdifferentiation has also been reported by lineage tracing in mouse islets in response to immunological stimuli (8). Data from human pancreas are scarce, but the limited information available suggests that similar changes in differentiation status may also occur in humans (9, 10).

Maintenance of beta-cell identity is determined by a tightly-regulated transcriptional network, consisting of proteins encoded by the Pancreatic and Duodenal Homeobox 1 (*PDX1*), Forkhead Box O1 (*FOXO1*), NK2 Homeobox 2 (*NKX2-2*), MAF BZIP Transcription Factor A (*MAFA*), NK6 Homeobox 1 (*NKX6-1*) and Paired Box 6 (*PAX6*) genes amongst others. Most of these do not display specific expression in beta cells but are also expressed in other pancreatic islet cell types; their role in cell identity definition is likely to involve the relative balance of expression (5, 11, 12). Among these, the *FOXO1* gene, a downstream effector of AKT signalling in beta cells (13), has been demonstrated to play a specific role in the maintenance of beta cell differentiation status in mice (3). In addition to its role in regulation of beta cell plasticity (14) and in stress responses (15), FOXO1 has also been demonstrated to regulate alternative splicing (a powerful interface between cell identity and cell stress) by moderation of splicing factor expression in human primary fibroblasts (16).

The cellular microenvironment created by diabetes is stressful for beta cells (17) and elevated glucose levels have been linked to reduced expression of nodal genes within the transcriptional network that controls beta cell identity at the level of total gene expression (18). Changes to beta cell differentiation status also occur in response to chronic hyperglycaemia (7, 19). Exposure of beta cells to the saturated fatty acid, palmitate or to pro-inflammatory cytokines, has also been shown to induce widespread changes to the beta cell transcriptome (20, 21). Altered beta cell identity may occur as a protective mechanism in response to a stressful extracellular milieu, with cellular plasticity serving to protect beta cells which might, otherwise, be lost via apoptosis. As such, this reversible plasticity may allow for later re-differentiation should the extracellular environment become more conducive (7). Such effects could be relevant to all beta cells but they may be particularly important for “hub cells” within islets, which are known to be more sensitive to insult than other beta cell subsets, resulting in beta cell failure (22, 23). A recent study has also shown that beta cells are heterogeneous and can be ordered into 3 major states characterised by relative insulin expression and ER stress levels. High ER stress and low insulin gene expression levels relate to a more immature beta cell state which doesn’t itself drive dedifferentiation but which may render them vulnerable to further insult (24).

We hypothesised that exposure to the cellular stressors that accompany the development of diabetes may cause disrupted regulation of key genes involved in the maintenance of beta cell identity, leading to changes in beta cell fate. We exposed human EndoC-βH1 beta cells in culture to a variety of diabetes-relevant cellular stressors and demonstrated alterations in the expression patterns of several key beta cell genes involved in the control of cell fate and cell identity and also in those controlling alternative splicing. Changes to the splicing patterns of 26% of genes were also apparent in human islets from donors with diabetes compared with non-diabetic controls. These changes were accompanied by alterations in hormone staining both *in vitro* and in *ex vivo*. These changes were reversible upon removal of the stressful stimulus or by moderation of splicing factor expression. Our observations suggest that altered splicing regulation and dysregulated splicing induced by cellular stress may influence the identity and proportions of hormone-expressing cells in human islets. Targeting splicing regulation may prove a fruitful avenue of investigation for future interventions to maintain beta cell differentiation in diabetes.

**Results**

*Key beta cell fate and function genes show disrupted expression in response to cell stress stimuli in EndoC-βH1 cells*

We tested the total expression levels of a panel of 31 preselected genes encoding hormones, determinants of beta cell identity or function and markers of cell stress in response to treatments designed to mimic aspects of diabetic pathophysiology. Large-scale changes in the expression of the majority of these genes were noted in response to all treatments across all time points (supplementary table S1; figure 1A-F). Notable amongst these were *MAFA, PAX6, NEUROD1, NKX2-2, PDX1, NKX6-1* and *PAX4* which are known to form an interacting network. All of these were responsive to either 25mM D-glucose or 0.5 mM palmitic acid. *PAX6, NKX2-2, PDX1, NKX6-1* and *PAX4* were similarly responsive to hypoxia. Differences in expression were also evident for *HES1, SOX9, NGN3 NANOG* and *POU5F1* genes which are markers for cellular plasticity. Markers of cellular stress including *DDIT3, ARNT,* and *MYC* also showed altered expression patterns under all conditions tested. Similar results were achieved following treatment with the generalised cellular stressor tunicamycin (supplementary table S2).

### Changes to the expression of splicing factors and beta cell splicing patterns in response to cell stress stimuli in EndoC-βH1 cells

We assessed the responses of genes encoding splicing factor genes, given their role in mediating the relationships between cell and environment (25, 26). Several splicing factors, notably the genes encoding *HNRNPA2B1, AKAP17A, PNSIR, SRSF1, SRSF2, SRSF3, SRSF6* and *LSM14A* demonstrated dysregulated expression in response to treatment with cytokines, hypoxia, high/low glucose or altered lipids (figure 2A; supplementary table S3). To determine whether these changes had functional significance, we also assessed the splicing patterns of key beta cell genes. Genes were included for analysis where they were known to produce alternatively-spliced isoforms with functionally different roles in beta cell function or differentiation status that we could identify uniquely. 3/5 genes fitting these criteria (*PAX6, PTPN1* and *POU5F1/OCT4)* demonstrated changes in the ratio of alternatively-expressed isoforms in response to treatment (figure 2B; supplementary table S4).

*26% of alternative splicing events demonstrate dysregulation in human islets form donors with T2D*

Assessment of transcriptome-wide patterns of alternative splicing demonstrated that of 47,944 splicing events detected in coding genes, 12,438 (26%) of splice sites in 6209 genes demonstrated altered usage (gene list available on request). Pathways analysis revealed that 6,209 genes demonstrated altered splicing events in T2D islets, and these were clustered in 196 pathways that grouped into 11 cellular functions. Of these, gene regulation amounted to the largest share at 17.65% of all pathways (figure 2 C1 and C2). GO analysis for biological processes and molecular function showed the top results were RNA metabolic process and RNA binding respectively (Supplementary table S5).

*Metabolic and ER stress is associated with gain of somatostatin positivity in EndoC-βH1 cells*

We quantified the effect of treatments designed to mimic the islet milieu in diabetes on cell identity in EndoC-βH1 cells cultured in vitro. A small (~5%) but consistent and statistically robust increase in the number of somatostatin-positive cells was noted in EndoC-βH1 cell cultures in response to incubation with elevated glucose levels (both D- and L-glucose) for 24h. Control cells, incubated in 5.5mM D-glucose showed no evidence of somatostatin immunopositivity (*p*=9.1 x 10^-5^, figure 3A). The proportion of somatostatin positive cells remained stable over longer periods of culture and no evidence of increased rates of cell death was seen in cells incubated under hyperglycaemic conditions (figure 3B). An increase in somatostatin positive cells was also noted in response to palmitic acid, hypoxia, pro-inflammatory cytokines, and tunicamycin (*p* = 5.2 x 10^-5^, *p*= 4.2 x 10^-5^, *p*=4.0 x 10^-3^, and *p* = 5.0 x 10^-3^ respectively; figure 3C). Conversely, no such increase was seen in response to oleic acid (*p*=0.343), although cells exposed to both palmitic acid and oleic acids concurrently responded in a similar manner to those treated with palmitate alone (*p*=0.003). No evidence of increased glucagon expression was noted in control or treated EndoC-βH1 cells at the protein level although a rise in *GCG* mRNA (and that encoding the transcription factor ARX) was evident in response to some treatment conditions. No change in the expression of *HHEX* mRNA transcripts was noted.

*Pancreatic sections from donors with T1D or T2D demonstrate increased numbers of somatostatin-positive cells*

To determine if the effects we had noted in EndoC-βH1 cells in vitro were also recapitulated in human pancreas, we determined the proportion of different endocrine cell types in islet preparations from donors with long duration T1D or T2D, and in control islets. We identified a significant increase in somatostatin-positive (delta) cell area in tissue sections from patients with either long duration T1D or T2D compared to control islets. The total somatostatin positive area in *in situ* islets from T1D or T2D donors compared to control islets was 17069.9 μM (3%) v/s 6166.24 μM (1%) for T2D (*p*= 2.47 x 10^-9^) and 31713 μM (9.3%) v/s 7360 μM (1% of islet area) for T1D (*p*= 2.1 x 10^-6^) (figure 4A and 4B; n=10 images for 5 cases and 5 controls). An assessment using direct counting of cells rather than staining area also showed significant increases in the number of delta cells (figure 4C). There was no increase in the total islet cell count. Pancreatic islets from donors with long duration T1D contained a median number of 8 delta cells out of a total cell count of 62 (IQR = 6) compared with 4.5 delta cells (IQR = 3) out of a total cell count of 67 for age and sex matched controls (p = 3.0x 10^-6^). Similarly, pancreatic islets from donors with long duration T2D contained a median number of 8.5 delta cells (IQR = 6) out of a total cell count of 77 (IQR = 6) compared with 5 delta cells (IQR = 3) out of a total cell count of 71 for age and sex matched controls (p = 2.2 x 10^-4^; supplementary table S6).

*Changes in hormone staining and beta cell expression patterns are reversible upon restoration of homeostatic conditions in EndoC-βH1 cells*

To determine whether the changes in cell identity and gene expression patterns we have observed were permanent or reversible, we exposed EndoC-βH1 cells to cellular stressors for 24 hours but then returned the cells to control conditions for 72 hrs prior to assessment. Following treatment with either high glucose or tunicamycin, we noted an increase in the number of somatostatin positive cells compared to controls (figure 5A*; p*=3.69 x 10^-9^ and *p*=1.3 x 10^-4^), and dysregulation of splicing ratios of candidate alternatively-spliced genes (figure 5B and 5C; supplementary table S7). Following return to control conditions, the number of somatostatin positive cells was indistinguishable from zero time point controls (figure 5A; *p*=0.568 and *p*=0.643), and patterns of alternative splicing were restored (figure 5B and 5C; supplementary table S7) indicating that the treatment induced changes we noted were reversible.

*The AKT inhibitor SH-6 restores splicing factor expression and abolishes somatostatin positivity in EndoC BH1 cells.*

The AKT pathway has previously been suggested to have a role in regulation of splicing and splicing factor activity (26). There was no emergence of somatostatin positive staining in cells treated with low concentration of the AKT inhibitor SH-6 alone, whereas cells treated with 25 mM glucose demonstrated an increase in SST positivity similar to that we observed previously (*p* = 0.0004). EndoC-βH1 cells treated with SH-6 and subsequently exposed to 25 mM glucose for 24 hrs however showed no increase in the number of somatostatin positive cells compared to 5 mM glucose controls; figure 6A and 6B). This effect was also noted at the level of splicing factor expression and alternative splicing (supplementary table S8).

## Discussion

In other cell types, alternative splicing forms a key part of the cellular defence against stress by adjusting the transcriptomic output of the cell to meet its new requirements (27). The cellular microenvironment induced by diabetes is stressful to beta cells, which may respond by either undergoing apoptosis (1, 2) or by altering their differentiation state (10, 28, 29) resulting in an overall loss of beta cell mass. We hypothesised that exposure to diabetes-related cellular stresses may disrupt the normal patterns of splicing occurring in beta cells, potentially leading to changes in beta cell fate or function. We report here that human EndoC-βH1 beta cells treated with diabetes-related cellular stressors such as altered glucose, fatty acids, cytokines or hypoxia, or with the ER stress inducer tunicamycin, displayed altered expression of genes involved in splicing regulation, as well as altered expression of important genes for cell fate or function in vitro. Furthermore, islet samples from patients with T2D diabetes displayed large-scale disruption to transcriptome-wide splicing patterns, with over a quarter of genes demonstrating splicing alterations. The main class of genes with altered splicing comprised genes themselves involved in mRNA processing and regulation of gene expression. These transcriptomic changes were accompanied by an increase in the number of somatostatin-positive cells in cultured EndoC-βH1 cells, an observation which was also evident in islet sections from human donors with either T1D or T2D. Similar effects were also noted when cells were exposed to L-glucose, suggesting that the response may occur in response to an osmotic stress and also that changes to beta cell differentiation status were not restricted to metabolic stress. The monounsaturated fatty acid oleate, a fatty acid that is not known to induce metabolic stress in human beta cells (30) elicited no response. The changes in cell hormone staining were reversible by either removal of the cellular stress, or by resetting splicing factor expression to their previous level using small molecule moderators of splicing factor expression. These observations are consistent with previous reports that stress-related changes in beta cell differentiation status are modifiable once the noxious stimuli is removed (18).

Beta cell differentiation status is influenced by a transcriptional network of genes including *PAX6, PDX1, NEUROD1, NKX2-2, NKX6-1, MAFA, PAX4* and *FOXO1* (31, 32) which demonstrated altered expression in our work. As the majority of these genes are also expressed in the other islet cell types, it is likely that beta cell maturity and identity is probably conferred by the subtle balance of these regulators. *PDX1* typically exhibits shared expression patterns across beta and delta cells, however here we found reduced expression of *PDX1* with concomitant gain of *SST* gene and protein expression, which suggests they may not be fully committed to a delta cell phenotype (33) and thus not display all the expected delta cell markers. Similarly, there was also no significant gain in the expression of the delta cell marker *HHEX,* which is required for delta cell differentiation (34)*.*  Further, *FOXO1* is a key player in determination of beta cell fate and function; targeted disruption of this gene in mouse embryos is known to result in cell fate changes in developing mouse pancreas (13). It is also a pivotal part of the beta cell stress response (5, 35). *FOXO1* and *PDX1* have also been shown to be mutually exclusive in the nucleus (5). In recent years, a new role for FOXO1 has also been identified as a regulator of the genes involved in alternative splicing decisions in some human cell types in the context of cellular senescence (16). Targeted knockdown of the *FOXO1* gene, or the use of inhibitor SH-6 that regulates FOXO1 activity, was able to restore splicing factor expression to levels seen in younger cells and restore cells to a younger phenotype (16). Alternative splicing has been well documented as a response to cellular stress (27). FOXO1 thus forms an interface between cell stress, alternative splicing and cell fate decisions. We have demonstrated here that transcripts encoding the Serine arginine rich (*SR*) and heterogeneous ribonucleoprotein particle (*HNRNP*) genes, which encode positive and negative regulators of alternative splicing respectively, were dysregulated in treated cells, as were patterns of alternative splicing for a limited number of functionally-relevant candidate genes tested. We also identified that at the transcriptome-wide level, 26% of all splicing events were altered in human primary islets from donors with T2D, indicating that this observation may also hold true in vivo. Interestingly, one of the predominant pathways demonstrating splicing dysregulation included those themselves involved in RNA metabolism and RNA processing. Although dysregulation of splicing regulators was not seen at the level of total gene expression as changes are likely cell type-specific and signal therefore diluted by the presence of other cell types, there were changes in the splicing patterns of these genes. This is particularly pertinent in that splicing factor genes are primarily themselves regulated by alternative splicing (36). Similar results have been previously reported for other diabetes-relevant tissues such as adipose tissue (37). In support of the theory that disrupted splicing may underpin the cell identity changes we see, restoration of splicing factor expression to levels consistent with control cells using the AKT inhibitor SH-6 which is known to influence splicing factor expression (16) abolished the emergence of the somatostatin-positive cell population, even in the presence of high glucose. Although SH-6 has not previously been shown to inhibit AKT in EndoC-βH1 cells, it has been shown to do so in another beta cell line RINm5F (38).

There are several potential explanations for the emergence of a somatostatin-positive population. Firstly, this could arise from proliferation of a population of delta-like cells or progenitor cells in the cultures, or in the primary islets. It is also possible that progenitor cells may be present in the culture but are pushing the cells to an insulin positive rather than somatostatin positive fate. However, the lack of somatostatin-positive staining in the absence of cell stress in the untreated EndoC-βH1 cell population suggests that expansion of a progenitor or delta cell population is an unlikely explanation for the emergence of somatostatin positivity. Similarly, immunofluorescence data from the islets of patients with T2D showed no increase in total cell counts which suggests that increased numbers of somatostatin-positive or insulin positive cells may not be the result of expansion of a small pool of progenitor cells. Another explanation is that a proportion of the beta cells may have undergone beta to delta transdifferentiation. This phenomenon has been reported previously in the mouse, where beta-to-alpha cell transitions have been documented (5, 6). The factors underlying apparent species differences may arise because of differences in islet architecture, gene expression and glucose sensing between rodent and human (39), but may also reflect profound differences in mRNA processing between these species since only 30% of splicing events are conserved between mice and humans (40).

We acknowledge that our study has limitations. Firstly, the changes in cell identity were observed in only a small proportion of the total cell population. The reasons for this are unclear but appear to be unrelated to the time of incubation since the response occurred very quickly (within 24h) and was then stable for several days in the continued presence of the stressor. One possibility is that the cultures are heterogeneous and that a small population of cells are more susceptible to stress-induced plasticity than the majority. It would be interesting to determine whether this putatively ‘plastic’ cell population shares features with cells such as those identified as ‘hubs’ in intact islets since these also display a greater degree of plasticity than other beta cell sub populations (41-44). While it may also be that the EndoC βH1 cells line is contaminated with progenitor cells, as discussed above, the data from the rescue experiments might suggest that this is not the case, given the absence of somatostatin positivity during normal culture conditions.

We also acknowledge that we have not conducted an exhaustive survey of splicing changes in the treated cells. The heterogeneous nature of the treated cultures means that only the largest effects would have been revealed by transcriptome-wide analysis. To circumvent this, it would be necessary to sort the somatostatin-positive cells from their unchanged neighbours. This is a technically demanding task in EndoC βH1, which does not grow well in low cell densities, and may be susceptible to shear stress (which may confound our findings). Somatostatin is also not a cell surface marker, rendering FACS sorting problematic. Taking into account these challenges, we opted to query transcriptome-wide splicing changes related to the presence of T2D in primary human islets, where we demonstrate that dysregulated splicing is clearly present.

We propose that our data are consistent with a model in which the appearance of a somatostatin-positive subpopulation can appear in response to the imposition of cellular stress both *in vitro* and *ex vivo*, and we postulate that this may reflect dysregulation of splicing factor genes with consequent changes to splicing patterns which may be mediated by altered activation of AKT signalling and changes to FOXO1 activity. This work is, to our knowledge, the first demonstration of the emergence of somatostatin positivity in human beta cells that may be specifically associated with changes in the regulation of mRNA splicing. Without lineage tracing experiments, which require expansion in non-human lines and are not trivial when using human model systems (45), it is not possible to infer that these changes we observed are directly due to beta to delta transdifferentiation. However, the consequence of the changes we observed may, at the molecular level, result in the altered expression of key genes involved in cell fate decisions. Targeting splicing patterns for therapeutic benefit is now an enticing possibility with several approaches already in clinical trials (46). We have shown that differentiation status in beta cells can be influenced by targeting splicing decisions, and a deeper understanding of the specific points at which such interventions could be focused might confer benefits for diabetes therapy in the future.

**Materials and Methods**

### Culture and treatment of EndoC-βH1 cells

EndoC-βH1 cells at Passage <25 were seeded in 24-well plates at a density of 6.0 x 10^5^ cells/ ml and maintained according to a modified humanised culture protocol as previously described (47) for 72 hrs prior to treatment. For assessment of the effects of altered glycaemia, cells were treated with 2.5 mM and 25 mM D- or L-glucose for 24, 36 or 48 hrs. Controls were maintained in 5 mM glucose media. For assessment of the effects of oxygen levels, cells were grown in 1%, 3% or 21% O_2_ for a period of 24, 36 or 48 hrs. For assessment of the effects of altered lipids, cells were treated with 0.5 mM palmitic acid, 0.5mM oleic acid or 0.5mM palmitic acid/0.5mM oleic acid for 12, 24 and 48 hrs. To determine the effects of pro-inflammatory cytokines, cells were treated with TNF
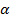
 (1000 U/ mL, INF
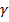
 (750 U/ mL) and IL1
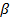
 (75 U/ mL) for 12, 24 and 36 hrs. For assessment of the effects of ER stress, cells were treated with 0.5 mM Tunicamycin for 24 hrs. Each treatment was carried out in 3 biological replicates, along with vehicle-only controls. Where cells were being used for subsequent immunofluorescence experiments they were cultured on 12 mm diameter coverslips, and treated according to the conditions described above before being fixed with 4% paraformaldehyde.

*Immunofluorescent characterisation of EndoC-βH1 cells in vitro*

The expression of mature beta cell markers, as well as islet cell hormones in EndoC-βH1 cells grown as described above was assessed using co-immunofluorescent microscopy. Briefly, following treatment, cells were fixed with 4% paraformaldehyde for 15 minutes at 4^o^C. Primary antibodies to SST, and GCG were diluted in phosphate buffered saline (PBS) with 0.1M Lysine, 10% donor calf serum, 0.02% sodium azide and 0.02% Triton X100 (ADST), to permeabilise the cell membranes and incubated overnight at 4^o^C. Primary antibodies were visualised using species specific highly cross-absorbed secondary antibodies (Invitrogen, Paisley) diluted in ADST at 1/400 and incubated for 1 hour at room temperature. Sequential staining was then performed with an antisera raised against insulin (Supplementary Table S9) diluted in ADST for 1 hour, followed by a goat anti guinea-pig Alexa Fluor 555 along with DAPI (Sigma-Aldrich; 1μg/ml) for 1 hour. Slides were visualised using a Leica AF6000 microscope (Leica, Milton Keynes, UK) and processed using the standard LASX Leica software platform. Ten randomly selected images were taken for each of the three biological replicates. Details of all antibodies are provided in Supplementary table S9.

*Immunofluorescent characterisation of human pancreatic tissue sections from patients with Type 1 or Type 2 Diabetes*

Immunofluorescence staining was used to determine the expression of islet cell hormones in formalin-fixed paraffin embedded (FFPE) pancreatic sections from individuals with long duration Type 1 diabetes (T1D), long duration Type 2 Diabetes (T2D) and individuals without diabetes from the Exeter Archival Diabetes Biobank (EADB; <http://foulis.vub.ac.be/> or the network of Pancreatic Organ Donors with Diabetes (nPOD) https://www.jdrfnpod.org/) (Supplemental Table S10). The majority of samples were taken from the pancreatic body, rather than the head or tail. All samples were studied with ethical approval (WoSREC4; 15/WS/0258). Following dewaxing and rehydration, pancreas sections were subjected to heat–induced epitope retrieval (HIER) in 10mmol/l citrate buffer (pH 6). The sections were then sequentially probed with antisera to somatostatin, glucagon and insulin (Supplemental Table S9) which were detected using species specific highly cross-absorbed secondary antibodies conjugated with fluorescent dyes (Invitrogen, Paisley, UK; Supplementary Table S9). Cell nuclei were stained with DAPI. Slides were visualised using a Leica AF6000 microscope (Leica, Milton Keynes, UK) and processed using the standard LASX Leica software platform. Quantification of pancreatic endocrine cells types within the islet was achieved firstly by assessing the somatostatin staining positive area as a percentage of total islet area between T1D/T2D patients and controls as described by others (48). To account for differences between the total islet area of T1D or T2D cases and their respective controls, total somatostatin positive areas were normalised to the global geometric mean of all samples. Secondly, effects of diabetes status on endocrine cell types was assessed by counting the number of delta cells compared to the total islet cell count. Ten randomly selected images from 7 cases and controls were taken for T1D and from 5 cases and controls for T2D. Areas of somatostatin, insulin and glucagon staining were selected and measured using Image J (49).

### RNA extraction, reverse transcription and assessment of total gene expression in EndoC-βH1 cells

EndoC-βH1 cells were washed in Dulbecco’s phosphate buffered saline (D-PBS) before RNA extraction using TRI® reagent to harvest RNA (Sigma-Aldrich, Steinheim, Germany). RNA concentrations were adjusted to 100 ng/ μL prior to reverse transcription. Synthesis of cDNA was carried out using Superscript® VILO™ cDNA synthesis kit (Thermo Fisher, Foster City USA) according to manufacturer’s instructions. Gene expression levels were measured in treated and untreated EndoC-βH1 cells by quantitative real-time PCR. A panel of 31 á priori target genes was selected on the basis that they had known roles in either beta cell fate or function, were markers of cell stress or were markers of other pancreatic endocrine cell types (supplementary table S11). Endogenous control genes (*PPIA, UBC, HPRT1, GUSB, B2M* and *IDH3B*) were empirically selected for stability in response to treatment*.* qRTPCR reaction mixes included 2.5 μL Taqman® Universal PCR mastermix II (no AmpErase® UNG) (Thermo Fisher, Waltham, MA, USA), 1.75 μL dH2O, 0.5 μL cDNA and 0.25 μL Taqman® gene assay (Thermo Fisher, Foster City USA) in a 5 μL reaction volume. Cycling conditions were: 50°C for 2 min, 95°C for 10 min and 50 cycles of 15 seconds at 95°C for 30 s and 1 minute at 60°C. Reactions were carried out in 3 biological replicates and 3 technical replicates. Assay Identifiers are given in Supplementary table S11. The relative expression of each test transcript was determined by the comparative Ct approach. Expression levels were calculated relative to the geometric mean of the endogenous controls, but also to the global mean of expression across all transcripts tested, (as one would with an array) which was empirically determined not to vary across test conditions to provide a robust baseline. Expression levels were normalised to the median level of expression seen in untreated EndoC-βH1 cell controls. Differences in gene expression levels between mock-treated and treated EndoC-βH1 cells were compared to the same time point controls for each condition and investigated for statistical significance by t-test carried out using SPSS version 23 (IBM, North Castle, NY, USA). Data were presented as means ± S.E.D.

### Determination of Splicing Factor expression in EndoC-βH1 cells

Splice site usage and thus patterns of alternative splicing are determined by the relative balance of serine-arginine rich RNA binding proteins (SRSFs; splice site activators) and heterogeneous nuclear ribonucleoprotein particles (HNNRPs; splice site inhibitors) at each splice site (50). Splicing factor Assay Ids are given in Supplementary Table S12. The expression levels of 20 splicing activator or splicing inhibitor genes was quantified in EndoC-βH1 cells using Taqman® Low Density Array (TLDA) on the Quantstudio 12K Flex system. Cycling conditions were 1 cycle each of 50^o^C for 2 minutes, 94.5^o^C for 10 minutes and then 40 cycles of 97^o^C for 30 seconds and 57.9^o^C for 1 minute. The reaction mixes included 50 μL Taqman® Fast Universal PCR Mastermix (Life Technologies, Foster City, USA), 30 μL dH_2_O and 20 μL cDNA template. The 100 μL reaction mix was dispensed into the TLDA card chamber and centrifuged twice for 1 minute at 1000 rpm to ensure the correct distribution of solution to each well and removal of bubbles. Transcript expression was assessed the by the comparative Ct approach relative to the endogenous control genes *IDH3B, GUSB* and *PPIA* which were selected based upon empirical evidence for stability in response to treatment and normalised to transcript expression in the untreated cells.

### Assessment of alternative splicing in EndoC-βH1 cells

Of the panel of 31 genes selected for total gene expression, 17 were known to exhibit alternative splicing. Of these, 5 were chosen for further analysis in EndoC-βH1 cells according to three criteria: i) unique, isoform-specific exon boundaries, ii) known functional differences between the isoforms, and iii) relevance of isoform differences to beta cell differentiation status or islet function. Isoforms of the *INS*, *PAX6, POU5F1 (OCT4), NANOG* and *PTPN1* genes fulfilled these criteria. Isoform-specific Taqman custom assays (Thermo Fisher, Waltham, MA, USA) were designed across unique splice boundaries that distinguish between the functionally distinct isoforms. Probe and primer sequences are given in Supplementary Table S13.

### Reversal of delta cell phenotype in EndoC-βH1 cells by restoration of homeostasis

To test for reversibility of effect in our system, cells were first treated with 25 mM glucose or 0.5 mM Tunicamycin for 24 hours as described above. Immediately following this, cells were restored to normal culture conditions for 72 hours prior to assessment of cell identity, total gene expression and splicing patterns.

*Reversal of delta cell phenotype in EndoC-βH1 cells by restoration of homeostasis or by small molecule inhibition of AKT pathway*

Previous reports suggest that changes to beta cell identity can be reversed upon removal of cellular stress (15). Reports have suggested that the AKT pathway, one of the signalling mechanisms whereby cells can communicate signals from stimuli such as cellular stress stimuli from the exterior of the cell is involved with regulation of splicing decisions and has also been linked with changes in beta cell plasticity (17, 25, 26). To test for reversibility of effect in our system, cells were first treated with 25 mM glucose or 0.5 mM Tunicamycin for 24 hrs as described above. Immediately following this, cells were restored to normal culture conditions for 72 hrs prior to assessment of cell identity, total gene expression and splicing patterns. To assess the effect of AKT inhibition on response to hyperglycaemia, cells were treated with 25 mM glucose alone, 1 μM of the AKT inhibitor SH-6 alone, or 25 mM glucose in combination with 1 μM SH-6 for 24 hrs prior to assessment of splicing patterns and cell identity as described above.

*Whole transcriptome analysis of patterns of alternative splicing in islets from human donors with T2D compared with non-diabetic human islets*

We assessed patterns of total gene expression and transcriptome wide patterns of alternative splicing in an initial discovery set of 6 islets preparations from donors with T2D versus 6 non-diabetic samples from the integrated islet distribution programme using the ClariomD pico system (Thermo Fisher, Waltham, MA, USA). A subsequent validation set of 6 islets preparations from donors with T2D versus 6 non-diabetic samples were also assessed to validate results from the first islets set. All islets were matched for age, gender and BMI. Sample preparation and gene chip cartridge arrays was performed by UK Bioinformatics Ltd (Woldingham, Caterham, Surrey). Transcriptome data for total gene and splicing expression was analysed using TAC 4.0.2.10 (Applied Biosystems, Thermo Fisher, Waltham, MA, USA). For both total expression and splicing genes were filtered to remove non-coding RNAs, pseudogenes and incompletely annotated genes. Differentially expressed genes and dysregulated splicing events with a *p* value of <0.05 in both islets sets were taken forward into pathways and gene ontology analysis carried out using Enrichr (51, 52).

**Acknowledgements**

The authors gratefully acknowledge Animal Free Research UK for funding (Postgraduate studentship to LWH). Additional support was from a JDRF Career Development Award (5-CDA-2014-221-A-N) to SJR. This research was performed with the support of the Network for Pancreatic Organ donors with Diabetes (nPOD; RRID:SCR_014641), a collaborative type 1 diabetes research project sponsored by JDRF (nPOD: 5-SRA-2018-557-Q-R) and The Leona M. & Harry B. Helmsley Charitable Trust (Grant#2018PG-T1D053). Organ Procurement Organizations (OPO) partnering with nPOD to provide research resources are listed at http://www.jdrfnpod.org//for-partners/npod-partners/. We also thank Mr. Ben Lee, Dr. Pia Leete and Dr. Mark Russell for technical assistance.

**Conflict of interest statement**

The authors declare no competing financial interests.

**References**

1 Butler, P.C., Meier, J.J., Butler, A.E. and Bhushan, A. (2007) The replication of [beta] cells in normal physiology, in disease and for therapy. *Nat Clin Pract End Met*, **3**, 758-768.

2 Marchetti, P., Del Guerra, S., Marselli, L., Lupi, R., Masini, M., Pollera, M., Bugliani, M., Boggi, U., Vistoli, F., Mosca, F. *et al.* (2004) Pancreatic islets from type 2 diabetic patients have functional defects and increased apoptosis that are ameliorated by metformin. *The Journal of clinical endocrinology and metabolism*, **89**, 5535-5541.

3 Talchai, C., Xuan, S., Lin, H.V., Sussel, L. and Accili, D. (2012) Pancreatic β-Cell Dedifferentiation As Mechanism Of Diabetic β-Cell Failure. *Cell*, **150**, 1223-1234.

4 van der Meulen, T. and Huising, M.O. (2015) The role of transcription factors in the transdifferentiation of pancreatic islet cells. *Journal of molecular endocrinology*, **54**, R103-R117.

5 Kitamura, T. (2013) The role of FOXO1 in [beta]-cell failure and type 2 diabetes mellitus. *Nat Rev Endocrinol*, **9**, 615-623.

6 Gao, T., McKenna, B., Li, C., Reichert, M., Nguyen, J., Singh, T., Yang, C., Pannikar, A., Doliba, N., Zhang, T. *et al.* (2014) Pdx1 Maintains β Cell Identity and Function by Repressing an α Cell Program. *Cell Metabolism*, **19**, 259-271.

7 Puri, S. and Hebrok, M. (2012) Diabetic β Cells: To Be or Not To Be? *Cell*, **150**, 1103-1104.

8 Piran, R., Lee, S.H., Li, C.R., Charbono, A., Bradley, L.M. and Levine, F. (2014) Pharmacological induction of pancreatic islet cell transdifferentiation: relevance to type I diabetes. *Cell Death Dis*, **5**, e1357.

9 White, M.G., Marshall, H.L., Rigby, R., Huang, G.C., Amer, A., Booth, T., White, S. and Shaw, J.A.M. (2013) Expression of Mesenchymal and α-Cell Phenotypic Markers in Islet β-Cells in Recently Diagnosed Diabetes. *Diabetes Care*, **36**, 3818-3820.

10 Spijker, H.S., Ravelli, R.B.G., Mommaas-Kienhuis, A.M., van Apeldoorn, A.A., Engelse, M.A., Zaldumbide, A., Bonner-Weir, S., Rabelink, T.J., Hoeben, R.C., Clevers, H. *et al.* (2013) Conversion of Mature Human β-Cells Into Glucagon-Producing α-Cells. *Diabetes*, **62**, 2471-2480.

11 Schaffer, A.E., Taylor, B.L., Benthuysen, J.R., Liu, J., Thorel, F., Yuan, W., Jiao, Y., Kaestner, K.H., Herrera, P.L., Magnuson, M.A. *et al.* (2013) Nkx6.1 controls a gene regulatory network required for establishing and maintaining pancreatic Beta cell identity. *PLoS Genet*, **9**, e1003274.

12 Guo, S., Dai, C., Guo, M., Taylor, B., Harmon, J.S., Sander, M., Robertson, R.P., Powers, A.C. and Stein, R. (2013) Inactivation of specific beta cell transcription factors in type 2 diabetes. *J Clin Invest*, **123**, 3305-3316.

13 Al-Masri, M., Krishnamurthy, M., Li, J., Fellows, G.F., Dong, H.H., Goodyer, C.G. and Wang, R. (2010) Effect of forkhead box O1 (FOXO1) on beta cell development in the human fetal pancreas. *Diabetologia*, **53**, 699-711.

14 Elghazi, L., Weiss, A.J., Barker, D.J., Callaghan, J., Staloch, L., Sandgren, E.P., Gannon, M., Adsay, V.N. and Bernal–Mizrachi, E. (2009) Regulation of Pancreas Plasticity and Malignant Transformation by Akt Signaling. *Gastroenterology*, **136**, 1091-1103.e1098.

15 Ponugoti, B., Dong, G. and Graves, D.T. (2012) Role of forkhead transcription factors in diabetes-induced oxidative stress. *Exp Diabetes Res*, **2012**, 939751.

16 Latorre, E., Ostler, E.O., Faragher, R.G.A. and Harries, L.W. (2018) FOXO1 and ETV6 genes may represent novel regulators of splicing factor expression in cellular senescence *FASEB Journal*, **33,** 1086-1097.

17 Li, N., Frigerio, F. and Maechler, P. (2008) The sensitivity of pancreatic beta-cells to mitochondrial injuries triggered by lipotoxicity and oxidative stress. *Biochem Soc Trans*, **36**, 930-934.

18 Swisa, A., Avrahami, D., Eden, N., Zhang, J., Feleke, E., Dahan, T., Cohen-Tayar, Y., Stolovich-Rain, M., Kaestner, K.H., Glaser, B. *et al.* (2017) PAX6 maintains β cell identity by repressing genes of alternative islet cell types. *The Journal of Clinical Investigation*, **127**, 230-243.

19 Brereton, M.F., Iberl, M., Shimomura, K., Zhang, Q., Adriaenssens, A.E., Proks, P., Spiliotis, I.I., Dace, W., Mattis, K.K., Ramracheya, R. *et al.* (2014) Reversible changes in pancreatic islet structure and function produced by elevated blood glucose. *Nature Communications*, **5**, 4639.

20 Cnop, M., Abdulkarim, B., Bottu, G., Cunha, D.A., Igoillo-Esteve, M., Masini, M., Turatsinze, J.V., Griebel, T., Villate, O., Santin, I. *et al.* (2014) RNA sequencing identifies dysregulation of the human pancreatic islet transcriptome by the saturated fatty acid palmitate. *Diabetes*, **63**, 1978-1993.

21 Ortis, F., Naamane, N., Flamez, D., Ladrière, L., Moore, F., Cunha, D.A., Colli, M.L., Thykjaer, T., Thorsen, K., Ørntoft, T.F. *et al.* (2010) Cytokines Interleukin-1β and Tumor Necrosis Factor-α Regulate Different Transcriptional and Alternative Splicing Networks in Primary β-Cells. *Diabetes*, **59**, 358-374.

22 Dorrell, C., Schug, J., Canaday, P.S., Russ, H.A., Tarlow, B.D., Grompe, M.T., Horton, T., Hebrok, M., Streeter, P.R., Kaestner, K.H. *et al.* (2016) Human islets contain four distinct subtypes of β cells. *Nature Communications*, **7**, 11756.

23 Johnston, Natalie R., Mitchell, Ryan K., Haythorne, E., Pessoa, Maria P., Semplici, F., Ferrer, J., Piemonti, L., Marchetti, P., Bugliani, M., Bosco, D. *et al.* Beta Cell Hubs Dictate Pancreatic Islet Responses to&#xa0;Glucose. *Cell Metabolism*, **24**, 389-401.

24 Xin, Y., Dominguez Gutierrez, G., Okamoto, H., Kim, J., Lee, A.-H., Adler, C., Ni, M., Yancopoulos, G.D., Murphy, A.J. and Gromada, J. (2018) Pseudotime Ordering of Single Human β-Cells Reveals States of Insulin Production and Unfolded Protein Response. *Diabetes*, **67**, 1783-1794.

25 Biamonti, G. and Caceres, J.F. (2009) Cellular stress and RNA splicing. *Trends Biochem Sci*, **34**, 146-153.

26 Long, Jennifer C. and Caceres, Javier F. (2009) The SR protein family of splicing factors: master regulators of gene expression. *Biochemical Journal*, **417**, 15-27.

27 Mastrangelo, A.M., Marone, D., Laido, G., De Leonardis, A.M. and De Vita, P. (2012) Alternative splicing: enhancing ability to cope with stress via transcriptome plasticity. *Plant Sci*, **185-186**, 40-49.

28 Wang, Z., York, N.W., Nichols, C.G. and Remedi, M.S. (2014) Pancreatic beta cell dedifferentiation in diabetes and redifferentiation following insulin therapy. *Cell Metab*, **19**, 872-882.

29 Fujimoto, K. and Polonsky, K.S. (2009) Pdx1 and other factors that regulate pancreatic beta-cell survival. *Diabetes, obesity & metabolism*, **11 Suppl 4**, 30-37.

30 Welters, H.J., Tadayyon, M., Scarpello, J.H., Smith, S.A. and Morgan, N.G. (2004) Mono-unsaturated fatty acids protect against beta-cell apoptosis induced by saturated fatty acids, serum withdrawal or cytokine exposure. *FEBS Lett*, **560**, 103-108.

31 Gosmain, Y., Katz, L.S., Masson, M.H., Cheyssac, C., Poisson, C. and Philippe, J. (2012) Pax6 Is Crucial for β-Cell Function, Insulin Biosynthesis, and Glucose-Induced Insulin Secretion. *Molecular Endocrinology*, **26**, 696-709.

32 Mitchell, R.K., Nguyen-Tu, M.-S., Chabosseau, P., Callingham, R.M., Pullen, T.J., Cheung, R., Leclerc, I., Hodson, D.J. and Rutter, G.A. (2017) The transcription factor Pax6 is required for pancreatic β cell identity, glucose-regulated ATP synthesis, and Ca2+ dynamics in adult mice. *Journal of Biological Chemistry*, **292**, 8892-8906.

33 DiGruccio, M.R., Mawla, A.M., Donaldson, C.J., Noguchi, G.M., Vaughan, J., Cowing-Zitron, C., Meulen, T. and Huising, M.O. (2016) Comprehensive alpha, beta and delta cell transcriptomes reveal that ghrelin selectively activates delta cells and promotes somatostatin release from pancreatic islets. *Mol Metab.*, **5**.

34 Zhang, J., McKenna, L.B., Bogue, C.W. and Kaestner, K.H. (2014) The diabetes gene Hhex maintains delta-cell differentiation and islet function. *Genes Dev*, **28**, 829-834.

35 Kobayashi, M., Kikuchi, O., Sasaki, T., Kim, H.-J., Yokota-Hashimoto, H., Lee, Y.-S., Amano, K., Kitazumi, T., Susanti, V.Y., Kitamura, Y.I. *et al.* (2012) FoxO1 as a double-edged sword in the pancreas: analysis of pancreas- and β-cell-specific FoxO1 knockout mice. *American Journal of Physiology - Endocrinology and Metabolism*, **302**, E603-E613.

36 Lareau, L. and E Brenner, S. (2015) *Regulation of Splicing Factors by Alternative Splicing and NMD Is Conserved between Kingdoms Yet Evolutionarily Flexible*.

37 Kaminska, D., Kakela, P., Nikkola, E., Venesmaa, S., Ilves, I., Herzig, K.H., Kolehmainen, M., Karhunen, L., Kuusisto, J., Gylling, H. *et al.* (2016) Regulation of alternative splicing in human obesity loci. *Obesity (Silver Spring)*, **24**, 2033-2037.

38 Tejedo, J.R., Cahuana, G.M., Ramirez, R., Esbert, M., Jimenez, J., Sobrino, F. and Bedoya, F.J. (2004) nitric oxide triggers the phosphatidylinositol 3-kinase/Akt survival pathway in insulin-producing RINm5F cells by arousing Src to activate insulin receptor substrate-1. *Endocrinology*, **145**, 2319-2327.

39 Benner, C., Meulen, T., Cacéres, E., Tigyi, K., Donaldson, C.J., Huising, M.O., Caceres, E., Tigyi, K., Donaldson, C.J. and Huising, M.O. (2014) The transcriptional landscape of mouse beta cells compared to human beta cells reveals notable species differences in long non-coding RNA and protein-coding gene expression. *BMC Genomics.*, **15**.

40 Barbosa-Morais, N.L., Irimia, M., Pan, Q., Xiong, H.Y., Gueroussov, S., Lee, L.J., Slobodeniuc, V., Kutter, C., Watt, S., Colak, R. *et al.* (2012) The evolutionary landscape of alternative splicing in vertebrate species. *Science*, **338**, 1587-1593.

41 Szabat, M., Lynn, F.C., Hoffman, B.G., Kieffer, T.J., Allan, D.W. and Johnson, J.D. (2012) Maintenance of β-Cell Maturity and Plasticity in the Adult Pancreas. *Developmental Biology Concepts in Adult Physiology*, **61**, 1365-1371.

42 Szabat, M., Piret, J.M., Luciani, D.S. and Johnson, J.D. (2009) Maturation of Adult β-Cells Revealed Using a Pdx1/Insulin Dual-Reporter Lentivirus. *Endocrinology*, **150**, 1627-1635.

43 Assouline-Thomas, B.a., Austin, E., Blaichman, J., Kapeluto, J., Moosavi, M., Petropavlovskaia, M., Hanley, S.C. and Rosenberg, L. (2010) β-Cell Mass Dynamics and Islet Cell Plasticity in Human Type 2 Diabetes. *Endocrinology*, **151**, 1462-1472.

44 Avrahami, D., Klochendler, A., Dor, Y. and Glaser, B. (2017) Beta cell heterogeneity: an evolving concept. *Diabetologia*, **60**, 1363-1369.

45 Furuyama, K., Chera, S., van Gurp, L., Oropeza, D., Ghila, L., Damond, N., Vethe, H., Paulo, J.A., Joosten, A.M., Berney, T. *et al.* (2019) Diabetes relief in mice by glucose-sensing insulin-secreting human α-cells. *Nature*, **567**, 43-48.

46 Havens, M.A., Duelli, D.M. and Hastings, M.L. (2013) Targeting RNA splicing for disease therapy. *Wiley Interdiscip Rev RNA*, **4**, 247-266.

47 Jeffery, N., Richardson, S., Beall, C. and Harries, L.W. (2017) The species origin of the cellular microenvironment influences markers of beta cell fate and function in EndoC-betaH1 cells. *Experimental cell research*, **361**, 284-291.

48 Plesner, A., Ten Holder, J.T. and Verchere, C.B. (2014) Islet remodeling in female mice with spontaneous autoimmune and streptozotocin-induced diabetes. *PLoS One*, **9**, e102843.

49 Schindelin, J., Arganda-Carreras, I., Frise, E., Kaynig, V., Longair, M., Pietzsch, T., Preibisch, S., Rueden, C., Saalfeld, S., Schmid, B. *et al.* (2012) Fiji: an open-source platform for biological-image analysis. *Nature Methods*, **9**, 676-682.

50 Cartegni, L., Chew, S.L. and Krainer, A.R. (2002) Listening to silence and understanding nonsense: exonic mutations that affect splicing. *Nat Rev Genet*, **3**, 285-298.

51 Kuleshov, M.V., Jones, M.R., Rouillard, A.D., Fernandez, N.F., Duan, Q., Wang, Z., Koplev, S., Jenkins, S.L., Jagodnik, K.M., Lachmann, A. *et al.* (2016) Enrichr: a comprehensive gene set enrichment analysis web server 2016 update. *Nucleic Acids Res*, **44**, W90-97.

52 Chen, E.Y., Tan, C.M., Kou, Y., Duan, Q., Wang, Z., Meirelles, G.V., Clark, N.R. and Ma'ayan, A. (2013) Enrichr: interactive and collaborative HTML5 gene list enrichment analysis tool. *BMC bioinformatics*, **14**, 128.

**Figure Legends**

**Figure 1. Effects of cell stresses on total gene expression of genes with roles in beta cell differentiation or function**. Volcano Plots demonstrating the most dysregulated genes in response to 24hr treatment with cellular stressors associated with a type 2 diabetic microenvironment. Volcano plots are illustrative of the numbers of genes dysregulated by cell stresses from qRTPCR data. *y* axis shows -log10 of *p* value, *x* axis shows differences in mean logged RQ (relative quantification) units between treatments and controls. Bonferroni corrections were performed to take account of the number of timepoints but not for the number of genes in the target panel as these are á priori. Upper line infers the Bonferroni multiple testing limit. The lower dotted line gives nominal significance. **A)** 25 mM high glucose. **B)** 2.5 mM low glucose. **C)** Hypoxia <3% O2. **D)** 0.5 mM Palmitic Acid. **E)** Cytokines TNFα, IL1β, INFγ. **F)** 0.5 mM Tunicamycin for ER stress.

**Figure 2. A. Effects of cell stresses on splicing factor expression.** Modified Manhattan-style plot corrected for direction of effect showing the effects of cellular stressors on splicing factor expression. The *y* axis shows -log10 of p value with direction of effect from data generated by Taqman Low Density Array (TLDA) cards. Bonferroni corrections were performed to take account of the number of treatments but not for the splicing regulators as these are known to be affected by cell stresses in a number of other cell types (16). Upper line infers the Bonferroni multiple testing limit. The lower dotted line gives nominal significance. Blue circles 25 mM high glucose. Green squares: 2.5mM low glucose.  Red diamonds: <3% O2.   Purple triangles: 0.5 mM Palmitic Acid. Orange triangles: Cytokines. Upper line infers the Bonferroni multiple testing limit. The lower dotted line gives nominal significance.

**B. Effects of cell insult treatments on alternatively spliced gene expression.** Modified Manhattan-style plot corrected for direction of effect**.***x* axis refs to the ratio of alternatively expressed isoforms at each locus from data generated by qRTPCR. Ratio was calculated by dividing expression of variants by expression of canonical transcripts. *y* axis shows -log10 of p value with direction of effect. Bonferroni corrections were performed to take account of the number of timepoints but not for the genes in the target panel as these are á priori. Upper line infers the Bonferroni multiple testing limit. The lower dotted line gives nominal significance. Blue circles: 25 mM glucose. Green squares: 2.5mM glucose.  Red diamonds: <3% O2.   Purple triangles: 0.5 mM Palmitic Acid. Pink inverse triangles: Cytokines. Orange hexagons: 0.5 mM. **C. The proportion of alternatively spliced genes demonstrating dysregulation in islets from donors with T2D**. Data generated by Clariom D pico array. **C1).** Pie chart showing the cellular and molecular functions of alternatively-spliced transcripts that display dysregulated splicing patterns in islets from donors with T2D compared with matched controls. **C2)** Pie chart showing the nature of splicing events that are dysregulated in islets from donors with T2D compared with matched controls.

**Figure 3. The effects of cell stressors to hormone expression in human beta cell line EndoC-βH1.** **A)** Immunofluorescence cytochemistry showing gain of somatostatin expression in cells treated with 25 mM high glucose. Cells were treated for 24, 36 and 48 hours and showed gains of somatostatin expression at all time points. There was no increase in the proportion of cells gaining somatostatin expression at later time points.  Representative image panels from 36 hour time point.  **A)** Panels 1 and 2 refer to treatment with 5 mM glucose as a control. Panels 3 and 4 refer to treatment with 25 mM glucose to mimic hyperglycaemia. The identity of the antibody used to stain is indicated in each panel. Of those cells that gained SST positivity, 17% were dual hormone positive. There were accompanying changes in the expression of INS and *SST* (*p*= 0.035, mean difference 0.223 and *p*=0.004 mean difference 0.211 respectively). B) Timecourse of somatostatin positivity in cultures of EndoC-βH1cells treated with high glucose (25mM). **C)** The number of Somatostatin positive cells in cultures of EndoC-βH1 cells treated with high glucose (25mM), Palmitic Acid (PI) 0.5mM, Hypoxia (3% O_2_), Cytokines (TNFα [1000 U/ mL], INFγ [750 U/ mL] and IL1β [75 U/ mL]), Tunicamycin (0.5mM) or oleic acid (0.5mM).

**Figure 4. Hormone staining patterns of donor islets from controls or individuals with T2D.** **A)** Panels 1 to 4 are representative Immunofluorescence images from human donor pancreatic tissue from controls (panels 1 and 2) and from cases of T1D compared to matched controls (panels 3 and 4). The identity of the antibody used to stain is indicated in each panel. **B)** Panels 5 to 8 are representative Immunofluorescence images from human donor pancreatic tissue from controls (panels 5 and 6) and from cases of T2D compared to matched controls (panels 7 and 8). **C)** Graph showing interquartile range from delta cell counts for individuals without Diabetes and patients with either T1D or T2D. We found a significant increase in the number of somatostatin positive cells in patients with either T1D Insulin-containing islets and insulin-deficient islets (*p* = 3.0 x 10^-6^) or T2D (*p* = 2.20 x 10^-4^) compared to their respective controls.

**Figure 5. Rescue of phenotype by restoration to normal culture conditions**. This figure illustrates the rescue of beta cell phenotype in EndoC-βH1 cells treated with 25mM glucose for 24hrs followed by restoration to 5 mM glucose culture conditions for 72 hrs. **A)** Panels 1 and 2 are representative Immunofluorescence images from untreated EndoC-βH1 cells **B)** Panels 3 and 4 are representative Immunofluorescence images from EndoC-βH1 cells treated with 25 mM glucose for 24hrs. **C)** Panels 5 and 6 are representative Immunofluorescence images from EndoC-βH1 cells treated with 25 mM glucose for 24hrs but then restored to 5 mM glucose for 72hrs. The identity of the antibody used to stain is indicated in each panel. Immunofluorescence image panel. **B)** The graph gives the total gene expression levels, generated by qRTPCR, for genes previously demonstrating dysregulated expression in response to glucose under treated and rescued conditions. **C)** The graph gives the isoform expression levels for genes previously demonstrating dysregulated splicing in response to glucose under treated and rescued conditions.

**Figure 6. Hormone staining and gene expression changes following restoration of splicing factor expression using the AKT inhibitor SH-6.** This figure illustrates the rescue of beta cell phenotype in EndoC-βH1 cells following restoration of splicing factor expression to that seen in normal glucose conditions. **A)** Panels 1 to 4 are representative Immunofluorescence images from untreated EndoC-βH1 cells. Panels 5 to 8 are representative Immunofluorescence images from EndoC-βH1 cells treated with 25mM glucose for 24hrs. Panels 9 to 12 are representative Immunofluorescence images from EndoC-βH1 cells treated with SH-6 alone. Panels 13 to 16 are representative Immunofluorescence images from EndoC-βH1 cells treated with 25mM glucose and SH-6 for 24hrs. The identity of the antibody used to stain is indicated in each panel. **B)** Graph showing the number of somatostatin positive cells under different culture conditions

**Abbreviations**

T1D – Type 1 diabetes, T2D – Type 2 diabetes, GO analysis – Gene Ontology analysis, FACS – fluorescence activated cell sorting, mRNA – messenger ribonucleic acid. qRTPCR – quantitative real time PCR, SST – Somatostatin, INS – Insulin, CGC – Glucagon, FOXO1 – Forkhead box 01, PAX6 – Paired box 6, SOX9 – SRY box 9, NKX6.1 – NK6 Homeobox 1, NKX6.2 – NK6 Homeobox 2, DDIT3 - DNA damage inducible transcript 3, SRSF – Serine Arginine Rich splicing factor, HNRNP – Heterogeneous ribonucleoprotein particle, MAFA - MAF BZIP Transcription Factor A.
