# Supplementary material for: Cellular stressors may alter islet hormone cell proportions by moderation of alternative splicing patterns
Source: Hum Mol Genet. 2019 May 17;28(16):2763–74. doi: 10.1093/hmg/ddz094 (PMC6687954; doi:10.1093/hmg/ddz094)

**Supplementary table S1: Effects of cell insult treatments on total gene expression.** The p values for statistical significance as determined by STUDENT INDEPENDENT T-TEST, mean logged fold change in expression, Standard errors difference (SED) and the 95% confidence intervals are given below. Results significant below the Bonferroni-adjusted value of p<0.016 for 3 time points are indicated in bold italic underlined text. Nominally-significant results are indicated in underlined italic text. Bonferroni corrections were performed to take account of the number of timepoints but not for the number of genes in the target panel as these are á priori.

| **Hyperglycaemia 25mM glucose assay Independent t-test p = 0.016** | | | | | | | | | | | | | | | | | | | | | | | | | | | | | | | | |
| --- | --- | --- | --- | --- | --- | --- | --- | --- | --- | --- | --- | --- | --- | --- | --- | --- | --- | --- | --- | --- | --- | --- | --- | --- | --- | --- | --- | --- | --- | --- | --- | --- |
| **24 hours** | | | | | | **36 hours** | | | | | | | | | | | | **48 hours** | | | | | | | | | | | | | | |
| **Gene** | **P value** | **Mean**  **Diff.** | **SED** | **95% C I** | | **Gene** | **P value** | | **Mean diff.** | | | **SED** | | **95% C I** | | | | **Gene** | | | | **P value** | | **Mean diff.** | | | **SED** | | **95% C I** | | | |
|  |  |  |  | **Lower** | **Upper** |  |  |  |  |  |  |  |  | **Lower** | | **Upper** | |  |  |  |  |  |  |  |  |  |  |  | **Lower** | | | **Upper** |
| *I****NS*** | ***0.001*** | ***0.338*** | ***0.072*** | ***0.178*** | ***0.498*** | *INS* | 0.229 | | 0.087 | | | 0.07 | | -0.06 | | -0.24 | | *INS* | | | | *0.035* | | *-0.223* | | | *0.09* | | 0.43 | | | *0.02* |
| *PDX1* | 0.210 | 0.047 | 0.035 | -0.031 | 0.125 | *PDX1* | 0.732 | | 0.016 | | | 0.04 | | -0.08 | | -0.11 | | *PDX1* | | | | 0.277 | | -0.205 | | | 0.14 | | 0.79 | | | -0.38 |
| ***PAX6*** | ***0.002*** | ***-0.172*** | ***0.040*** | ***-0.262*** | ***-0.081*** | *PAX6* | 0.496 | | -0.034 | | | 0.05 | | -0.14 | | -0.07 | | *PAX6* | | | | 0.168 | | -0.274 | | | 0.13 | | 0.82 | | | -0.27 |
| *FOXO1* | 0.166 | -0.444 | 0.126 | -1.860 | 0.972 | *FOXO1* | 0.628 | | -0.292 | | | 0.51 | | -2.5 | | -1.92 | | *FOXO1* | | | | 0.195 | | 0.292 | | | 0.15 | | 0.35 | | | -0.94 |
| ***NEUROD1*** | ***0.000*** | ***-0.533*** | ***0.045*** | ***-0.635*** | ***-0.430*** | *NEUROD1* | 0.639 | | -0.304 | | | 0.56 | | -2.69 | | -2.08 | | *NEUROD1* | | | | 0.231 | | 0.216 | | | 0.13 | | 0.32 | | | -0.75 |
| ***NKX2-2*** | ***0.003*** | ***-0.471*** | ***0.116*** | ***-0.734*** | ***-0.207*** | *NKX2-2* | 0.487 | | -0.483 | | | 0.57 | | -2.91 | | -1.95 | | *NKX2-2* | | | | 0.255 | | 0.387 | | | 0.25 | | 0.63 | | | -1.40 |
| ***MAFA*** | ***0.004*** | ***0.109*** | ***0.030*** | ***0.043*** | ***0.175*** | *MAFA* | ***0.007*** | | ***0.120*** | | | ***0.04*** | | **-0.04** | | ***-0.20*** | | *MAFA* | | | | 0.856 | | 0.014 | | | 0.07 | | 0.25 | | | -0.28 |
| *MAFB* | 0.055 | -0.056 | 0.026 | -0.114 | 0.001 | *MAFB* | 0.619 | | -0.036 | | | 0.06 | | 0.29 | | -0.22 | | *MAFB* | | | | 0.886 | | 0.015 | | | 0.09 | | 0.38 | | | -0.41 |
| *GCK* | 0.573 | -0.030 | 0.051 | -0.144 | 0.084 | *GCK* | 0.083 | | -0.085 | | | 0.04 | | 0.18 | | -0.01 | | *GCK* | | | | 0.315 | | 0.049 | | | 0.05 | | 0.05 | | | -0.15 |
| *SYP* | 0.086 | -0.581 | 0.091 | -1.542 | 0.380 | *SYP* | 0.598 | | -0.334 | | | 0.54 | | 2.64 | | -1.97 | | *SYP* | | | | 0.305 | | 0.303 | | | 0.22 | | 0.64 | | | -1.25 |
| *SLC2A2* | 0.547 | 0.033 | 0.053 | -0.085 | 0.150 | *SLC2A2* | 0.562 | | 0.033 | | | 0.06 | | 0.09 | | -0.16 | | *SLC2A2* | | | | 0.198 | | 0.084 | | | 0.06 | | 0.05 | | | -0.22 |
| *PTPN1* | *0.026* | *-0.072* | *0.028* | *-0.133* | *-0.011* | *PTPN1* | 0.943 | | 0.005 | | | 0.06 | | 0.22 | | -0.23 | | *PTPN1* | | | | 0.385 | | -0.079 | | | 0.07 | | 0.37 | | | -0.21 |
| ***PAX4*** | 0.465 | 0.068 | 0.077 | -0.235 | 0.370 | *PAX4* | ***0.002*** | | ***0.145*** | | | ***0.03*** | | **-0.07** | | ***-0.22*** | | *PAX4* | | | | 0.243 | | -0.192 | | | 0.12 | | 0.68 | | | -0.30 |
| *STK11* | 0.198 | -0.042 | 0.031 | -0.110 | 0.026 | *STK11* | *0.025* | | *-0.107* | | | *0.04* | | 0.2 | | *0.02* | | *STK11* | | | | 0.132 | | -0.059 | | | 0.04 | | 0.14 | | | -0.02 |
| *SLC16A1* | 0.663 | -0.019 | 0.042 | -0.112 | 0.074 | *SLC16A1* | 0.120 | | -0.087 | | | 0.05 | | 0.2 | | -0.03 | | *SLC16A1* | | | | 0.308 | | -0.159 | | | 0.12 | | 0.64 | | | -0.32 |
| ***LDHA*** | **0.001** | **0.108** | **0.024** | **0.053** | **0.162** | *LDHA* | 0.175 | | 0.039 | | | 0.03 | | 0.02 | | -0.10 | | *LDHA* | | | | ***0.003*** | | ***0.125*** | | | ***0.03*** | | -0.05 | | | ***-0.20*** |
| ***GCG*** | 0.307 | -0.067 | 0.062 | -0.204 | 0.071 | GCG | 0.409 | | -0.060 | | | 0.07 | | -0.21 | | 0.09 | | GCG | | | | ***0.012*** | | ***0.224*** | | | ***0.07*** | | 0.06 | | | ***0.39*** |
| ***SST*** | 0.260 | 0.060 | 0.050 | -0.052 | 0.171 | SST | 0.269 | | 0.060 | | | 0.05 | | -0.05 | | 0.17 | | SST | | | | ***0.004*** | | ***0.211*** | | | ***0.06*** | | 0.08 | | | ***0.34*** |
| *ARX* | 0.944 | 0.006 | 0.088 | -0.189 | 0.202 | ARX | 0.309 | | 0.091 | | | 0.08 | | -0.1 | | 0.28 | | ARX | | | | 0.480 | | 0.058 | | | 0.08 | | -0.12 | | | 0.23 |
| ***ARNT*** | **0.013** | **0.107** | **0.033** | **0.030** | **0.184** | ARNT | 0.723 | | 0.022 | | | 0.06 | | -0.14 | | 0.18 | | ARNT | | | | 0.144 | | 0.148 | | | 0.08 | | -0.09 | | | 0.39 |
| *DDIT3* | 0.142 | 0.069 | 0.043 | -0.028 | 0.166 | DDIT3 | 0.386 | | 0.055 | | | 0.06 | | -0.08 | | 0.19 | | DDIT3 | | | | 0.062 | | 0.113 | | | 0.05 | | -0.01 | | | 0.23 |
| *HIF1A* | 0.416 | 0.070 | 0.082 | -0.113 | 0.253 | HIF1A | 0.701 | | 0.035 | | | 0.09 | | -0.16 | | 0.23 | | HIF1A | | | | 0.051 | | 0.208 | | | 0.09 | | 0 | | | 0.42 |
| ***MYC*** | 0.959 | -0.008 | 0.140 | -0.590 | 0.574 | MYC | ***0.001*** | | ***-0.183*** | | | ***0.04*** | | -0.27 | | ***0.10*** | | MYC | | | | ***0.000*** | | ***0.313*** | | | ***0.04*** | | 0.23 | | | ***0.40*** |
| ***SOX9*** | 0.613 | -0.053 | 0.101 | -0.277 | 0.172 | SOX9 | 0.033 | | 0.269 | | | 0.11 | | 0.03 | | 0.51 | | SOX9 | | | | ***0.001*** | | ***0.277*** | | | ***0.06*** | | 0.15 | | | ***0.41*** |
| *POU5F1* | *0.032* | *-0.072* | *0.029* | *-0.137* | *-0.008* | POU5F1 | 0.215 | | -0.057 | | | 0.04 | | -0.15 | | 0.04 | | POU5F1 | | | | 0.333 | | -0.096 | | | 0.08 | | -0.4 | | | 0.21 |
| ***NANOG*** | 0.176 | 0.067 | 0.046 | -0.036 | 0.169 | NANOG | 0.194 | | 0.076 | | | 0.05 | | -0.05 | | 0.20 | | NANOG | | | | ***0.008*** | | ***0.165*** | | | ***0.05*** | | 0.05 | | | ***0.28*** |
| ***HES1*** | ***0.002*** | ***-0.161*** | ***0.040*** | ***-0.250*** | ***-0.073*** | HES1 | ***0.000*** | | ***0.243*** | | | ***0.05*** | | 0.14 | | ***0.34*** | | HES1 | | | | ***0.003*** | | ***-0.149*** | | | ***0.04*** | | -0.24 | | | ***-0.06*** |
| *NGN3* | 0.365 | -0.045 | 0.047 | -0.151 | 0.061 | NGN3 | 0.193 | | -0.062 | | | 0.04 | | -0.16 | | 0.04 | | NGN3 | | | | 0.375 | | -0.052 | | | 0.06 | | -0.18 | | | 0.07 |
| **Hypoglycaemia 2.5mM glucose assay Independent t-test p=0016** | | | | | | | | | | | | | | | | | | | | | | | | | | | | | | | | |
| **24 hours** | | | | | | **36 hours** | | | | | | | | | | | | | **48 hours** | | | | | | | | | | | | | |
| **Gene** | **P value** | **Mean diff.** | **SED** | **95% C I** | | **Gene** | **P value** | | | **Mean diff** | | **SED** | | **95% C I** | | | | **Gene** | | | | **P value** | | | **Mean diff** | | **SED** | | **95% C I** | | | |
|  |  |  |  | **Lower** | **Upper** |  |  |  |  |  |  |  |  | **Lower** | | **Upper** | |  |  |  |  |  |  |  |  |  |  |  | **Lower** | | | **Upper** |
| ***INS*** | *0.038* | *0.183* | *0.08* | *0.01* | 0.35 | INS | 0.994 | | | 0.001 | | 0.06 | | -0.14 | | 0.14 | | INS | | | | ***0.015*** | | | ***-0.284*** | | ***0.10*** | | ***-0.5*** | | | ***-0.07*** |
| ***PDX1*** | ***0.003*** | ***-0.148*** | ***0.04*** | ***-0.23*** | ***-0.06*** | PDX1 | ***0.000*** | | | ***-0.191*** | | ***0.04*** | | ***0.27*** | | ***-0.11*** | | PDX1 | | | | ***0.001*** | | | ***-0.224*** | | ***0.05*** | | ***-0.33*** | | | ***-0.12*** |
| ***PAX6*** | 0.058 | -0.415 | 0.11 | -0.86 | 0.03 | PAX6 | ***0.000*** | | | ***0.269*** | | ***0.04*** | | ***0.35*** | | ***-0.19*** | | PAX6 | | | | ***0.000*** | | | ***-0.239*** | | ***0.04*** | | ***-0.34*** | | | ***-0.14*** |
| ***FOXO1*** | ***0.000*** | ***0.303*** | ***0.04*** | ***0.21*** | ***0.39*** | FOXO1 | ***0.000*** | | | ***0.372*** | | ***0.04*** | | ***-0.28*** | | ***0.46*** | | FOXO1 | | | | ***0.000*** | | | ***0.427*** | | ***0.05*** | | ***0.32*** | | | ***0.54*** |
| ***NEUROD1*** | ***0.000*** | ***0.301*** | ***0.03*** | ***0.22*** | ***0.38*** | NEUROD1 | ***0.000*** | | | ***0.348*** | | ***0.04*** | | ***-0.26*** | | ***0.43*** | | NEUROD1 | | | | ***0.000*** | | | ***0.299*** | | ***0.05*** | | ***0.19*** | | | ***0.41*** |
| ***NKX2-2*** | ***0.000*** | ***0.580*** | ***0.08*** | ***0.39*** | ***0.77*** | NKX2-2 | ***0.002*** | | | ***0.386*** | | ***0.09*** | | ***-0.17*** | | ***0.60*** | | NKX2-2 | | | | ***0.001*** | | | ***0.409*** | | ***0.09*** | | ***0.21*** | | | ***0.61*** |
| ***MAFA*** | 0.983 | 0.002 | 0.07 | -0.29 | 0.3 | MAFA | ***0.001*** | | | ***-0.161*** | | ***0.03*** | | ***0.24*** | | ***-0.08*** | | MAFA | | | | 0.067 | | | -0.292 | | 0.08 | | -0.63 | | | 0.05 |
| *MAFB* | 0.084 | 0.044 | 0.02 | -0.01 | 0.1 | MAFB | 0.816 | | | -0.016 | | 0.06 | | 0.25 | | 0.22 | | MAFB | | | | 0.635 | | | -0.038 | | 0.07 | | -0.32 | | | 0.25 |
| *GCK* | *0.037* | *0.121* | *0.05* | *0.01* | 0.23 | GCK | 0.063 | | | 0.102 | | 0.05 | | 0.01 | | 0.21 | | GCK | | | | 0.857 | | | 0.030 | | 0.13 | | -1.43 | | | 1.49 |
| ***SYP*** | ***0.000*** | ***0.427*** | ***0.03*** | ***0.35*** | ***0.5*** | SYP | ***0.000*** | | | ***0.420*** | | ***0.04*** | | ***-0.34*** | | ***0.50*** | | SYP | | | | ***0.000*** | | | ***0.421*** | | ***0.04*** | | ***0.33*** | | | ***0.51*** |
| ***SLC2A2*** | ***0.024*** | ***0.145*** | ***0.05*** | ***0.02*** | ***0.27*** | SLC2A2 | ***0.005*** | | | ***0.183*** | | ***0.05*** | | ***-0.07*** | | ***0.30*** | | SLC2A2 | | | | 0.757 | | | 0.019 | | 0.06 | | -0.12 | | | 0.16 |
| ***PTPN1*** | ***0.000*** | ***-0.190*** | ***0.03*** | ***0.26*** | ***-0.12*** | PTPN1 | *0.032* | | | *-0.082* | | *0.03* | | -0.16 | | *-0.01* | | PTPN1 | | | | ***0.001*** | | | ***-0.184*** | | ***0.04*** | | ***-0.27*** | | | ***-0.09*** |
| ***PAX4*** | 0.382 | -0.042 | 0.05 | 0.14 | 0.06 | PAX4 | 0.063 | | | 0.069 | | 0.03 | | 0 | | 0.14 | | PAX4 | | | | ***0.000*** | | | ***-0.211*** | | ***0.04*** | | ***-0.3*** | | | ***-0.12*** |
| ***STK11*** | ***0.001*** | ***-0.154*** | ***0.03*** | ***0.23*** | ***-0.08*** | STK11 | 0.312 | | | -0.043 | | 0.04 | | -0.13 | | 0.05 | | STK11 | | | | ***0.000*** | | | ***-0.206*** | | ***0.04*** | | ***-0.29*** | | | ***-0.12*** |
| ***SLC16A1*** | ***<0.001*** | ***-0.307*** | ***0.05*** | ***0.42*** | ***-0.19*** | SLC16A1 | ***0.000*** | | | ***-0.301*** | | ***0.04*** | | ***-0.39*** | | ***-0.21*** | | SLC16A1 | | | | ***0.000*** | | | ***-0.361*** | | ***0.05*** | | ***-0.48*** | | | ***-0.24*** |
| *LDHA* | 0.159 | 0.155 | 0.07 | -0.14 | 0.45 | LDHA | 0.485 | | | -0.057 | | 0.07 | | -0.33 | | 0.22 | | LDHA | | | | 0.249 | | | 0.189 | | 0.12 | | -0.31 | | | 0.69 |
| ***GCG*** | 0.518 | -0.036 | 0.05 | -0.16 | 0.08 | GCG | 0.468 | | | -0.053 | | 0.07 | | -0.21 | | 0.10 | | GCG | | | | ***0.001*** | | | ***0.311*** | | ***0.07*** | | ***0.16*** | | | ***0.46*** |
| ***SST*** | 0.559 | 0.030 | 0.05 | -0.08 | 0.14 | SST | 0.189 | | | 0.078 | | 0.06 | | -0.05 | | 0.20 | | SST | | | | ***0.000*** | | | ***0.259*** | | ***0.05*** | | ***0.15*** | | | ***0.36*** |
| *ARX* | 0.831 | 0.018 | 0.08 | -0.16 | 0.2 | ARX | 0.625 | | | 0.041 | | 0.08 | | -0.14 | | 0.22 | | ARX | | | | *0.020* | | | *-0.241* | | *0.09* | | -0.44 | | | *-0.05* |
| ***ARNT*** | ***0.000*** | ***0.185*** | ***0.04*** | ***0.11*** | ***0.26*** | ARNT | 0.062 | | | 0.135 | | 0.06 | | -0.01 | | 0.28 | | ARNT | | | | 0.311 | | | 0.066 | | 0.06 | | -0.07 | | | 0.20 |
| ***DDIT3*** | ***0.001*** | ***0.195*** | ***0.04*** | ***0.11*** | ***0.28*** | DDIT3 | ***0.004*** | | | ***0.165*** | | ***0.04*** | | ***0.07*** | | ***0.26*** | | DDIT3 | | | | 0.269 | | | 0.197 | | 0.13 | | -0.34 | | | 0.74 |
| *HIF1A* | 0.091 | 0.153 | 0.08 | -0.03 | 0.34 | HIF1A | 0.355 | | | 0.080 | | 0.08 | | -0.1 | | 0.26 | | HIF1A | | | | 0.501 | | | -0.804 | | 0.99 | | -5.04 | | | 3.43 |
| ***MYC*** | ***0.004*** | ***0.136*** | ***0.04*** | ***0.05*** | 0.22 | MYC | 0.262 | | | 0.238 | | 0.16 | | -0.41 | | 0.89 | | MYC | | | | 0.657 | | | 0.019 | | 0.04 | | -0.08 | | | 0.11 |
| ***SOX9*** | 0.059 | 0.221 | 0.10 | -0.01 | 0.45 | SOX9 | ***0.005*** | | | ***0.421*** | | ***0.12*** | | ***0.16*** | | ***0.68*** | | SOX9 | | | | ***0.015*** | | | ***0.371*** | | ***0.12*** | | ***0.09*** | | | ***0.65*** |
| ***POU5F1*** | ***0.000*** | ***-0.208*** | ***0.04*** | ***-0.29*** | ***-0.12*** | POU5F1 | ***0.001*** | | | ***-0.152*** | | ***0.03*** | | ***-0.22*** | | ***-0.08*** | | POU5F1 | | | | ***0.001*** | | | ***-0.220*** | | ***0.04*** | | ***-0.32*** | | | ***-0.12*** |
| *NANOG* | 0.060 | 0.106 | 0.05 | -0.01 | 0.22 | NANOG | 0.186 | | | -0.081 | | 0.06 | | -0.21 | | -0.05 | | NANOG | | | | 0.426 | | | 0.119 | | 0.12 | | -0.37 | | | 0.61 |
| *HES1* | 0.120 | -0.095 | 0.06 | -0.22 | 0.03 | HES1 | *0.024* | | | *0.119* | | *0.04* | | -0.02 | | *-0.22* | | HES1 | | | | 0.714 | | | -0.053 | | 0.13 | | -0.58 | | | 0.47 |
| **Hypoxia 3% O2 assay Independent t-test p = 0.016** | | | | | | | | | | | | | | | | | | | | | | | | | | | | | | | | |
| **4hrs** | | | | | | **12 hrs** | | | | | | | | | | | | **24hrs** | | | | | | | | | | | | | | |
| **Gene** | **P value** | **Mean diff.** | **SED** | **95% CI** | | **Gene** | **P value** | | | **Mean diff.** | | **SED** | | **95% CI** | | | | **Gene** | | | | **P value** | | | **Mean diff.** | | **SED** | | **95% CI** | | | |
|  |  |  |  | **Lower** | **Upper** |  |  |  |  |  |  |  |  | **Lower** | | **Upper** | |  |  |  |  |  |  |  |  |  |  |  | **Lower** | | | **Upper** |
| *NGN3* | 0.715 | -0.024 | 0.058 | -0.255 | 0.207 | NGN3 | 0.125 | | | -0.137 | | 0.056 | | -0.360 | | 0.086 | | NGN3 | | | | *0.031* | | | *0.082* | | *0.033* | | *0.009* | | | *0.155* |
| *NANOG* | 0.819 | 0.012 | 0.051 | -0.101 | 0.125 | NANOG | 0.130 | | | -0.085 | | 0.052 | | -0.201 | | 0.03 | | NANOG | | | | 0.763 | | | 0.013 | | 0.043 | | -0.082 | | | 0.109 |
| ***POU5F1*** | 0.261 | -0.064 | 0.053 | -0.182 | 0.055 | POU5F1 | ***0.004*** | | | ***-0.194*** | | ***0.053*** | | ***-0.312*** | | -***0.075*** | | POU5F1 | | | | ***0.015*** | | | ***-0.160*** | | ***0.054*** | | ***-0.280*** | | | ***-0.039*** |
| ***INS*** | 0.561 | -0.042 | 0.071 | -0.200 | 0.115 | INS | 0.053 | | | -0.154 | | 0.070 | | -0.309 | | 0.002 | | INS | | | | ***0.002*** | | | ***-0.296*** | | ***0.072*** | | ***-0.457*** | | | ***-0.135*** |
| ***PDX1*** | 0.134 | -0.303 | 0.128 | -0.821 | 0.216 | PDX1 | ***0.000*** | | | ***-0.332*** | | ***0.047*** | | ***-0.437*** | | ***-0.227*** | | PDX1 | | | | ***0.000*** | | | ***-0.261*** | | ***0.042*** | | ***-0.354*** | | | ***-0.168*** |
| ***PAX6*** | *0.037* | *-0.092* | *0.038* | *-0.178* | -0.007 | PAX6 | ***0.000*** | | | ***-0.328*** | | ***0.048*** | | ***-0.434*** | | ***-0.222*** | | PAX6 | | | | ***0.000*** | | | ***-0.379*** | | ***0.040*** | | ***-0.467*** | | | ***-0.291*** |
| ***SLC16A1*** | ***0.000*** | ***-0.139*** | ***0.025*** | ***-0.196*** | ***-0.083*** | SLC16A1 | ***0.005*** | | | ***-0.201*** | | ***0.057*** | | ***-0.328*** | | ***-0.075*** | | SLC16A1 | | | | *0.023* | | | *-0.114* | | *0.042* | | *-0.209* | | | -0.02 |
| *FOXO1* | 0.936 | -0.005 | 0.065 | -0.151 | 0.141 | FOXO1 | 0.281 | | | 0.075 | | 0.066 | | -0.071 | | 0.221 | | FOXO1 | | | | *0.048* | | | *-0.148* | | *0.066* | | *-0.294* | | | -0.002 |
| *NEUROD1* | 0.488 | -0.048 | 0.066 | -0.195 | 0.1 | NEUROD1 | 0.355 | | | 0.067 | | 0.069 | | -0.087 | | 0.221 | | NEUROD1 | | | | 0.479 | | | -0.049 | | 0.067 | | -0.198 | | | 0.1 |
| ***NKX2-2*** | *0.040* | *0.125* | *0.052* | *0.007* | *0.243* | NKX2-2 | ***0.015*** | | | ***0.165*** | | ***0.055*** | | ***0.040*** | | ***0.29*** | | NKX2-2 | | | | 0.192 | | | -0.075 | | 0.053 | | -0.196 | | | 0.045 |
| *SYP* | 0.231 | 0.065 | 0.050 | -0.049 | 0.179 | SYP | *0.031* | | | *0.153* | | *0.060* | | *0.017* | | 0.29 | | SYP | | | | *0.036* | | | *0.122* | | *0.050* | | *0.010* | | | 0.234 |
| ***NKX6-1*** | 0.997 | 0.000 | 0.100 | -0.223 | 0.222 | NKX6-1 | ***0.004*** | | | ***0.446*** | | ***0.118*** | | ***0.183*** | | ***0.708*** | | NKX6-1 | | | | ***0.005*** | | | ***0.369*** | | ***0.102*** | | ***0.142*** | | | ***0.596*** |
| *MAFA* | 0.891 | 0.007 | 0.052 | -0.123 | 0.109 | MAFA | 0.272 | | | 0.057 | | 0.049 | | -0.052 | | 0.166 | | MAFA | | | | 0.801 | | | 0.012 | | 0.046 | | -0.091 | | | 0.115 |
| ***LDHA*** | ***0.000*** | ***0.163*** | ***0.026*** | ***0.104*** | ***0.222*** | LDHA | ***0.000*** | | | ***0.346*** | | ***0.025*** | | ***0.289*** | | ***0.402*** | | LDHA | | | | ***0.000*** | | | ***0.412*** | | ***0.025*** | | ***0.357*** | | | ***0.467*** |
| *MAFB* | 0.711 | 0.014 | 0.037 | -0.068 | 0.096 | MAFB | 0.959 | | | -0.002 | | 0.037 | | -0.084 | | 0.08 | | MAFB | | | | 0.373 | | | -0.034 | | 0.037 | | -0.117 | | | 0.048 |
| *GCG* | 0.722 | 0.076 | 0.208 | -0.387 | 0.539 | GCG | 0.098 | | | 0.380 | | 0.208 | | -0.083 | | 0.843 | | GCG | | | | 0.653 | | | -0.096 | | 0.208 | | -0.559 | | | 0.367 |
| ***SST*** | ***0.000*** | ***0.096*** | ***0.018*** | ***0.056*** | ***0.137*** | SST | 0.249 | | | 0.025 | | 0.021 | | -0.021 | | 0.071 | | SST | | | | ***0.000*** | | | ***0.139*** | | ***0.018*** | | ***0.099*** | | | ***0.178*** |
| *ARNT* | 0.899 | 0.003 | 0.021 | -0.044 | 0.049 | ARNT | 0.246 | | | 0.027 | | 0.022 | | -0.022 | | 0.076 | | ARNT | | | | 0.844 | | | 0.004 | | 0.022 | | -0.044 | | | 0.053 |
| *DDIT3* | 0.458 | 0.029 | 0.038 | -0.055 | 0.113 | DDIT3 | 0.805 | | | -0.010 | | 0.040 | | -0.100 | | 0.08 | | DDIT3 | | | | 0.248 | | | -0.054 | | 0.044 | | -0.151 | | | 0.044 |
| *ARX* | 0.118 | 0.057 | 0.033 | -0.017 | 0.132 | ARX | *0.018* | | | *-0.102* | | *0.036* | | *-0.183* | | -0.022 | | ARX | | | | 0.383 | | | -0.035 | | 0.039 | | -0.122 | | | 0.051 |
| ***GCK*** | ***0.001*** | ***0.159*** | ***0.036*** | ***0.079*** | ***0.239*** | GCK | ***0.000*** | | | ***0.360*** | | ***0.019*** | | ***0.316*** | | ***0.405*** | | GCK | | | | ***0.000*** | | | ***0.415*** | | ***0.030*** | | ***0.349*** | | | ***0.482*** |
| ***HIF1A*** | 0.245 | 0.036 | 0.029 | -0.029 | 0.1 | HIF1A | 0.314 | | | -0.032 | | 0.030 | | -0.099 | | 0.035 | | HIF1A | | | | ***0.008*** | | | ***-0.108*** | | ***0.032*** | | ***-0.181*** | | | ***-0.036*** |
| *MYC* | ***0.004*** | ***0.170*** | ***0.044*** | ***0.071*** | ***0.268*** | MYC | 0.689 | | | 0.019 | | 0.045 | | -0.083 | | 0.121 | | MYC | | | | 0.918 | | | 0.008 | | 0.077 | | -0.163 | | | 0.18 |
| ***SLC2A2*** | ***0.002*** | ***0.088*** | ***0.022*** | ***0.040*** | ***0.137*** | SLC2A2 | ***0.001*** | | | ***0.092*** | | ***0.020*** | | ***0.048*** | | ***0.135*** | | SLC2A2 | | | | 0.063 | | | -0.159 | | 0.046 | | -0.338 | | | 0.019 |
| ***SOX9*** | 0.335 | 0.126 | 0.125 | -0.152 | 0.404 | SOX9 | 0.453 | | | 0.120 | | 0.153 | | -0.222 | | 0.462 | | SOX9 | | | | ***0.000*** | | | ***0.659*** | | ***0.123*** | | ***0.386*** | | | ***0.933*** |
| *PTPN1* | *0.049* | *-0.065* | *0.029* | *-0.130* | *0.001* | PTPN1 | 0.070 | | | -0.203 | | 0.062 | | -0.442 | | 0.036 | | PTPN1 | | | | 0.315 | | | -0.255 | | 0.193 | | -1.074 | | | 0.563 |
| ***PAX4*** | ***0.001*** | ***-0.097*** | ***0.021*** | ***-0.144*** | -***0.05*** | PAX4 | 0.151 | | | -0.106 | | 0.049 | | -0.297 | | 0.086 | | PAX4 | | | | 0.057 | | | -0.196 | | 0.053 | | -0.407 | | | 0.014 |
| ***STK11*** | 0.145 | -0.334 | 0.144 | -0.948 | 0.28 | STK11 | 0.121 | | | -0.119 | | 0.052 | | -0.336 | | 0.078 | | STK11 | | | | ***0.000*** | | | ***-0.341*** | | ***0.021*** | | ***-0.244*** | | | ***-0.149*** |
| **Hypoxia 1% O2 assay Independent Samples Test p= 0.016** | | | | | | | | | | | | | | | | | | | | | | | | | | | | | | | | |
| **4hrs** | | | | | | **12hrs** | | | | | | | | | | | | | | | | **24 hours** | | | | | | | | | | |
| **Gene** | **P value** | **Mean diff.** | **SED** | **95% CI** | | **Gene** | **P value** | | | **Mean diff.** | | **SED** | | **95% CI** | | | | **Gene** | | | | **P value** | | | **Mean diff.** | | **SED** | | **95% CI** | | | |
|  |  |  |  | **Lower** | **Upper** |  |  |  |  |  |  |  |  | **Lower** | | **Upper** | |  |  |  |  |  |  |  |  |  |  |  | **Lower** | | | **Upper** |
| *NGN3* | *0.017* | *-0.080* | *0.028* | *-0.143* | -0.017 | NGN3 | 0.555 | | | -0.080 | | 0.653 | | -2.350 | | 3.268 | | NGN3 | | | | 0.566 | | | -0.077 | | 0.096 | | -1.212 | | | 1.058 |
| ***NANOG*** | 0.145 | -0.072 | 0.046 | -0.175 | 0.03 | NANOG | ***0.001*** | | | ***-0.072*** | | ***0.051*** | | ***-0.343*** | | ***-0.115*** | | NANOG | | | | 0.799 | | | -0.015 | | 0.056 | | -0.140 | | | 0.111 |
| ***POU5F1*** | *0.044* | *-0.120* | *0.052* | *-0.237* | -0.004 | POU5F1 | ***0.001*** | | | ***-0.120*** | | ***0.066*** | | ***-0.466*** | | ***-0.174*** | | POU5F1 | | | | 0.225 | | | -0.083 | | 0.063 | | -0.226 | | | 0.061 |
| ***INS*** | ***0.007*** | ***-0.299*** | ***0.088*** | ***-0.495*** | ***-0.104*** | INS | 0.094 | | | -0.299 | | 0.087 | | -0.353 | | 0.033 | | INS | | | | *0.045* | | | *-0.202* | | *0.087* | | *-0.400* | | | -0.005 |
| ***PDX1*** | ***0.000*** | ***-0.375*** | ***0.046*** | ***-0.479*** | ***-0.272*** | PDX1 | ***0.000*** | | | ***-0.375*** | | ***0.059*** | | ***-0.464*** | | ***-0.201*** | | PDX1 | | | | ***0.001*** | | | ***-0.271*** | | ***0.051*** | | ***-0.388*** | | | ***-0.155*** |
| ***PAX6*** | ***0.001*** | ***-0.204*** | ***0.044*** | ***-0.303*** | ***-0.105*** | PAX6 | ***0.000*** | | | ***-0.204*** | | ***0.046*** | | ***-0.637*** | | ***-0.434*** | | PAX6 | | | | ***0.000*** | | | ***-0.371*** | | ***0.050*** | | ***-0.484*** | | | ***-0.258*** |
| ***SLC16A1*** | ***0.000*** | ***-0.251*** | ***0.044*** | ***-0.349*** | ***-0.152*** | SLC16A1 | ***0.000*** | | | ***-0.251*** | | ***0.045*** | | ***-0.611*** | | ***-0.41*** | | SLC16A1 | | | | ***0.007*** | | | ***-0.178*** | | ***0.052*** | | ***-0.295*** | | | -***0.061*** |
| *FOXO1* | *0.017* | *0.196* | *0.068* | *0.044* | 0.348 | FOXO1 | 0.566 | | | 0.196 | | 0.064 | | -0.181 | | 0.105 | | FOXO1 | | | | *0.041* | | | *-0.217* | | *0.091* | | *-0.424* | | | -0.011 |
| *NEUROD1* | 0.483 | 0.048 | 0.066 | -0.100 | 0.196 | NEUROD1 | 0.895 | | | 0.048 | | 0.072 | | -0.170 | | 0.151 | | NEUROD1 | | | | 0.111 | | | -0.159 | | 0.090 | | -0.363 | | | 0.044 |
| *NKX2-2* | 0.161 | 0.105 | 0.069 | -0.050 | 0.26 | NKX2-2 | 0.502 | | | 0.105 | | 0.117 | | -0.354 | | 0.539 | | NKX2-2 | | | | 0.155 | | | -0.114 | | 0.073 | | -0.282 | | | 0.053 |
| ***SYP*** | ***0.011*** | ***0.175*** | ***0.055*** | ***0.050*** | ***0.299*** | SYP | 0.831 | | | 0.175 | | 0.077 | | -0.158 | | 0.191 | | SYP | | | | 0.757 | | | 0.020 | | 0.063 | | -0.165 | | | 0.125 |
| ***NKX6-1*** | ***0.011*** | ***0.302*** | ***0.098*** | ***0.085*** | ***0.519*** | NKX6-1 | 0.440 | | | 0.302 | | 0.113 | | -0.343 | | 0.161 | | NKX6-1 | | | | 0.365 | | | -0.114 | | 0.119 | | -0.384 | | | 0.156 |
| *MAFA* | 0.347 | 0.045 | 0.046 | -0.057 | 0.147 | MAFA | 0.306 | | | 0.045 | | 0.453 | | -1.322 | | 2.557 | | MAFA | | | | 0.453 | | | 1.090 | | 0.939 | | -10.80 | | | 12.98 |
| ***LDHA*** | ***0.000*** | ***0.253*** | ***0.027*** | ***0.193*** | ***0.312*** | LDHA | ***0.000*** | | | ***0.253*** | | ***0.053*** | | ***0.335*** | | ***0.572*** | | LDHA | | | | ***0.000*** | | | ***0.539*** | | ***0.031*** | | ***0.469*** | | | ***0.609*** |
| ***MAFB*** | 0.643 | 0.021 | 0.043 | -0.117 | 0.076 | MAFB | ***0.016*** | | | ***0.021*** | | ***0.054*** | | ***-0.275*** | | ***-0.036*** | | MAFB | | | | 0.186 | | | 0.069 | | 0.049 | | -0.180 | | | 0.04 |
| *GCG* | 0.827 | 0.047 | 0.210 | -0.422 | 0.516 | GCG | 0.402 | | | 0.047 | | 0.209 | | -0.283 | | 0.648 | | GCG | | | | 0.117 | | | -0.445 | | 0.257 | | -1.027 | | | 0.136 |
| *SST* | 0.088 | 0.134 | 0.045 | -0.047 | 0.315 | SST | 0.928 | | | 0.134 | | 0.097 | | -0.398 | | 0.418 | | SST | | | | *0.029* | | | *0.057* | | *0.022* | | *0.007* | | | 0.108 |
| *ARNT* | 0.649 | 0.014 | 0.029 | -0.051 | 0.079 | ARNT | 0.535 | | | 0.014 | | 0.085 | | -0.414 | | 0.289 | | ARNT | | | | 0.856 | | | 0.021 | | 0.093 | | -1.073 | | | 1.116 |
| ***DDIT3*** | *0.029* | *0.101* | *0.040* | *0.013* | 0.189 | DDIT3 | ***0.000*** | | | ***0.101*** | | ***0.052*** | | ***0.240*** | | ***0.47*** | | DDIT3 | | | | *0.026* | | | *0.122* | | *0.046* | | *0.018* | | | 0.227 |
| ***ARX*** | 0.090 | -0.102 | 0.055 | -0.225 | 0.019 | ARX | 0.099 | | | -0.102 | | 0.081 | | -0.542 | | 0.095 | | ARX | | | | ***0.000*** | | | ***-0.270*** | | ***0.051*** | | ***-0.386*** | | | ***-0.155*** |
| ***GCK*** | ***0.000*** | ***0.272*** | ***0.029*** | ***0.208*** | ***0.338*** | GCK | *0.034* | | | *0.272* | | *0.088* | | *0.079* | | 0.792 | | GCK | | | | ***0.000*** | | | ***0.337*** | | ***0.017*** | | ***0.300*** | | | ***0.375*** |
| ***HIF1A*** | 0.311 | 0.046 | 0.043 | -0.050 | 0.143 | HIF1A | ***0.005*** | | | ***0.046*** | | ***0.041*** | | ***-0.240*** | | ***-0.057*** | | HIF1A | | | | ***0.000*** | | | ***0.301*** | | ***0.037*** | | ***-0.385*** | | | ***-0.219*** |
| ***MYC*** | 0.757 | 0.051 | 0.147 | -0.518 | 0.621 | MYC | 0.185 | | | 0.051 | | 0.076 | | -0.061 | | 0.277 | | MYC | | | | ***0.000*** | | | ***0.528*** | | ***0.083*** | | ***0.341*** | | | ***0.715*** |
| ***SLC2A2*** | 0.369 | 0.069 | 0.062 | -0.181 | 0.321 | SLC2A2 | 0.244 | | | 0.069 | | 0.123 | | -0.721 | | 0.321 | | SLC2A2 | | | | ***0.000*** | | | ***-0.232*** | | ***0.025*** | | ***-0.288*** | | | ***-0.177*** |
| ***SOX9*** | 0.757 | 0.041 | 0.129 | -0.246 | 0.328 | SOX9 | ***0.002*** | | | ***0.041*** | | ***0.147*** | | ***0.324*** | | ***0.987*** | | SOX9 | | | | ***0.000*** | | | ***1.090*** | | ***0.145*** | | ***0.763*** | | | ***1.418*** |
| ***PTPN1*** | ***0.000*** | ***-0.146*** | ***0.029*** | ***-0.210*** | ***-0.082*** | PTPN1 | *0.024* | | | *-0.146* | | *0.084* | | *-0.826* | | *-0.152* | | PTPN1 | | | | ***0.000*** | | | ***-0.289*** | | ***0.037*** | | ***-0.373*** | | | ***-0.207*** |
| ***PAX4*** | ***0.000*** | ***-0.144*** | ***0.025*** | ***-0.200*** | ***-0.089*** | PAX4 | *0.050* | | | *-0.144* | | *0.094* | | *-0.786* | | *-0.001* | | PAX4 | | | | 0.077 | | | -0.665 | | 0.086 | | -1.673 | | | 0.342 |
| ***STK11*** | ***0.004*** | ***-0.109*** | ***0.029*** | ***-0.174*** | ***-0.044*** | STK11 | 0.079 | | | -0.109 | | 0.075 | | -0.556 | | *0.067* | | STK11 | | | | ***0.000*** | | | ***-0.156*** | | ***0.023*** | | ***-0.209*** | | | ***-0.104*** |
| **Lipotoxicity 0.5 mM Independent Samples Test p = 0.016** | | | | | | | | | | | | | | | | | | | | | | | | | | | | | | | | |
| **12hrs** | | | | | | **24hrs** | | | | | | | | | | | | **48hrs** | | | | | | | | | | | | | | |
| **Gene** | **P value** | **Mean diff.** | **SED** | **95% CI** | | **Gene** | **P value** | | | **Mean diff.** | | **SED** | | **95% CI** | | | | **Gene** | | | | **P value** | | | **Mean. Diff.** | | **SED** | | **95% CI** | | | |
|  |  |  |  | **Lower** | **Upper** |  |  |  |  |  |  |  |  | **Lower** | | **Upper** | |  |  |  |  |  |  |  |  |  |  |  | **Lower** | | | **Upper** |
| *FOXO1* | 0.909 | -0.005 | 0.049 | -0.114 | 0.103 | FOXO1 | 0.077 | | | -0.104 | | 0.053 | | -0.222 | | 0.013 | | FOXO1 | | | | 0.492 | | | -0.036 | | 0.051 | | -0.078 | | | 0.151 |
| ***INS*** | 0.591 | 0.049 | 0.089 | -0.150 | 0.249 | INS | ***0.001*** | | | ***0.412*** | | ***0.094*** | | ***0.203*** | | ***0.623*** | | INS | | | | 0.531 | | | 0.072 | | 0.112 | | -0.176 | | | 0.321 |
| ***PDX1*** | ***0.014*** | ***-0.199*** | ***0.067*** | ***-0.349*** | ***-0.050*** | PDX1 | *0.017* | | | *0.158* | | *0.055* | | *0.035* | | *0.282* | | PDX1 | | | | 0.464 | | | 0.044 | | 0.057 | | -0.084 | | | 0.171 |
| ***NEUROD1*** | 0.652 | 0.016 | 0.035 | -0.061 | 0.094 | NEUROD1 | 0.583 | | | -0.021 | | 0.037 | | -0.103 | | 0.061 | | NEUROD1 | | | | ***0.000*** | | | ***0.357*** | | ***0.032*** | | ***0.285*** | | | ***0.429*** |
| ***SLC16A1*** | ***0.000*** | ***0.162*** | ***0.023*** | ***0.112*** | ***0.212*** | SLC16A1 | 0.189 | | | -0.040 | | 0.029 | | -0.104 | | 0.023 | | SLC16A1 | | | | 0.08 | | | -0.047 | | 0.024 | | -0.100 | | | 0.007 |
| *SYP* | 0.984 | -0.015 | 0.737 | -1.658 | 1.627 | SYP | 0.944 | | | 0.052 | | 0.738 | | -1.591 | | 1.696 | | SYP | | | | *0.047* | | | *1.668* | | *0.738* | | *0.024* | | | *3.313* |
| ***GCK*** | 0.285 | -0.056 | 0.050 | -0.167 | 0.055 | GCK | ***0.007*** | | | ***-0.166*** | | ***0.050*** | | ***-0.277*** | | ***-0.056*** | | GCK | | | | ***0.000*** | | | ***0.787*** | | ***0.050*** | | ***0.676*** | | | ***0.898*** |
| ***MAFA*** | 0.065 | -0.085 | 0.041 | -0.178 | 0.006 | MAFA | 0.185 | | | -0.064 | | 0.045 | | -0.165 | | 0.036 | | MAFA | | | | ***0.000*** | | | ***0.636*** | | ***0.039*** | | ***0.550*** | | | ***0.724*** |
| ***NKX2.2*** | 0.090 | *-0.106* | 0.057 | -0.233 | 0.020 | NKX2.2 | 0.818 | | | 0.013 | | 0.057 | | -0.114 | | 0.141 | | NKX2.2 | | | | ***0.003*** | | | ***-0.224*** | | ***0.058*** | | ***-0.353*** | | | ***-0.096*** |
| ***LDHA*** | 0.837 | -0.008 | 0.039 | -0.095 | 0.079 | LDHA | 0.347 | | | 0.037 | | 0.038 | | -0.048 | | 0.123 | | LDHA | | | | ***0.000*** | | | ***-0.295*** | | ***0.037*** | | ***-0.378*** | | | ***-0.214*** |
| ***MAFB*** | 0.085 | *-0.203* | 0.068 | -0.470 | 0.063 | MAFB | ***0.008*** | | | ***-0.096*** | | ***0.029*** | | ***-0.161*** | | ***-0.031*** | | MAFB | | | | 0.225 | | | 0.123 | | 0.073 | | -0.170 | | | 0.416 |
| ***NKX6.1*** | ***0.010*** | ***-0.149*** | ***0.047*** | ***-0.254*** | ***-0.044*** | NKX6.1 | *0.044* | | | *0.108* | | *0.047* | | *0.004* | | *0.214* | | NKX6.1 | | | | ***0.000*** | | | ***-0.347*** | | ***0.049*** | | ***-0.455*** | | | ***-0.239*** |
| ***ARNT*** | ***0.010*** | ***0.102*** | ***0.032*** | ***0.031*** | ***0.175*** | ARNT | 0.055 | | | -0.071 | | 0.033 | | -0.144 | | 0.002 | | ARNT | | | | ***0.000*** | | | ***-0.165*** | | ***0.032*** | | ***-0.237*** | | | ***-0.095*** |
| ***PAX4*** | 0.431 | 0.057 | 0.070 | -0.098 | 0.212 | PAX4 | 0.213 | | | 0.099 | | 0.075 | | -0.067 | | 0.265 | | PAX4 | | | | ***0.000*** | | | ***1.251*** | | ***0.068*** | | ***1.100*** | | | ***1.404*** |
| ***SST*** | ***0.001*** | ***0.183*** | ***0.041*** | ***0.093*** | ***0.274*** | SST | *0.021* | | | *0.117* | | *0.043* | | *0.022* | | *0.213* | | SST | | | | ***0.000*** | | | ***-0.500*** | | ***0.046*** | | ***-0.603*** | | | ***-0.398*** |
| ***DDIT3*** | 0.893 | -0.004 | 0.035 | -0.083 | 0.074 | DDIT3 | ***0.000*** | | | ***-0.261*** | | ***0.044*** | | ***-0.359*** | | ***-0.164*** | | DDIT3 | | | | ***0.000*** | | | ***-1.320*** | | ***0.042*** | | ***-1.415*** | | | ***-1.226*** |
| ***GCG*** | *0.026* | *-0.173* | *0.066* | *-0.321* | *-0.026* | GCG | ***0.000*** | | | ***-0.503*** | | ***0.065*** | | ***-0.649*** | | ***-0.358*** | | GCG | | | | ***0.000*** | | | ***1.209*** | | ***0.067*** | | ***1.061*** | | | ***1.359*** |
| ***PAX6*** | 0.331 | -0.049 | 0.048 | -0.156 | 0.058 | PAX6 | *0.047* | | | *-0.108* | | *0.048* | | *-0.215* | | *-0.002* | | PAX6 | | | | ***0.009*** | | | ***0.176*** | | ***0.055*** | | ***0.054*** | | | ***0.300*** |
| ***MYC*** | ***0.005*** | ***0.421*** | ***0.117*** | ***0.161*** | ***0.681*** | MYC | 0.154 | | | 0.156 | | 0.102 | | -0.070 | | 0.384 | | MYC | | | | ***0.001*** | | | ***-0.588*** | | ***0.103*** | | ***-0.739*** | | | ***-0.279*** |
| ***NANOG*** | 0.796 | -0.020 | 0.077 | -0.192 | 0.151 | NANOG | 0.374 | | | -0.072 | | 0.078 | | -0.246 | | 0.101 | | NANOG | | | | ***0.000*** | | | ***-0.669*** | | ***0.084*** | | ***-0.858*** | | | ***-0.482*** |
| ***SLC2A2*** | ***0.014*** | ***-0.104*** | ***0.035*** | ***-0.183*** | ***-0.026*** | SLC2A2 | *0.440* | | | *0.106* | | *0.113* | | *-0.353* | | *0.566* | | SLC2A2 | | | | ***0.000*** | | | ***-0.228*** | | ***0.037*** | | ***-0.311*** | | | ***-0.147*** |
| *HIF1A* | *0.023* | *-0.084* | *0.030* | *-0.154* | *-0.015* | HIF1A | *0.022* | | | *-0.205* | | *0.076* | | *-0.374* | | *-0.037* | | HIF1A | | | | 0.301 | | | -0.062 | | 0.057 | | -0.190 | | | 0.065 |
| ***PTPN1*** | 0.570 | 0.015 | 0.027 | -0.044 | 0.075 | PTPN1 | 0.089 | | | -0.047 | | 0.025 | | -0.104 | | 0.009 | | PTPN1 | | | | ***0.004*** | | | ***0.111*** | | ***0.030*** | | ***0.045*** | | | ***0.177*** |
| *STK11* | 0.509 | 0.036 | 0.053 | -0.081 | 0.153 | STK11 | 0.534 | | | -0.038 | | 0.059 | | -0.170 | | 0.094 | | STK11 | | | | 0.211 | | | -0.070 | | 0.053 | | -0.188 | | | 0.047 |
| *ARX* | 0.612 | 0.043 | 0.083 | -0.142 | 0.229 | ARX | 0.053 | | | 0.176 | | 0.080 | | -0.003 | | 0.355 | | ARX | | | | 0.339 | | | -0.075 | | 0.075 | | -0.242 | | | 0.091 |
| *SLC2A4* | 0.382 | -0.113 | 0.124 | -0.163 | 0.389 | SLC2A4 | 0.197 | | | *0.158* | | 0.115 | | -0.097 | | 0.414 | | SLC2A4 | | | | 0.288 | | | 0.136 | | 0.122 | | -0.135 | | | 0.409 |
| ***SOX9*** | 0.255 | 0.136 | 0.113 | -0.115 | 0.387 | SOX9 | ***0.012*** | | | ***0.329*** | | ***0.107*** | | ***0.091*** | | ***0.567*** | | SOX9 | | | | ***0.000*** | | | ***-1.430*** | | ***0.120*** | | ***-1.699*** | | | ***-1.162*** |
| ***HES1*** | ***0.013*** | ***-0.208*** | ***0.069*** | ***-0.363*** | ***-0.054*** | HES1 | *0.22* | | | *0.082* | | *0.063* | | *-0.058* | | *0.223* | | HES1 | | | | *0.019* | | | *-1.738* | | *0.254* | | *-2.801* | | | *-0.676* |
| ***NGN3*** | ***0.004*** | ***0.165*** | ***0.044*** | ***0.067*** | ***0.264*** | NGN3 | ***0.001*** | | | ***0.212*** | | ***0.044*** | | ***0.115*** | | ***0.310*** | | NGN3 | | | | ***0.000*** | | | ***0.338*** | | ***0.045*** | | ***0.237*** | | | ***0.440*** |
| *POU5F1* | *0.024* | *0.165* | *0.062* | *0.026* | *0.304* | POU5F1 | ***0.015*** | | | ***-0.187*** | | ***0.064*** | | ***-0.331*** | | ***-0.044*** | | POU5F1 | | | | *0.043* | | | *0.146* | | *0.063* | | *0.005* | | | *0.288* |
| **Cytokine assay TNF𝛂 INF𝛄 and IL1𝛃 Independent Samples Test p = 0.025** | | | | | | | | | | | | | | | | | | | | | | | | | | | | | | | | |
| **12 hours** | | | | | | **24 hours** | | | | | | | | | | | | **36 hours** | | | | | | | | | | | | | | |
| **Gene** | **P value** | **Mean diff.** | **SED.** | **95% CI** | | **Gene** | **P value** | **Mean diff.** | | | **SED.** | | **95% CI** | | | | **Gene** | | | | **P value** | | **Mean diff.** | | | **SED** | | **95% CI** | | | | |
|  |  |  |  | **Lower** | **Upper** |  |  |  |  |  |  |  | **Lower** | | **Upper** | |  |  |  |  |  |  |  |  |  |  |  | **Lower** | | **Upper** | | |
| ***FOXO1*** | 0.244 | -0.060 | 0.049 | -0.168 | 0.048 | FOXO1 | *0.021* | *-0.168* | | | *0.062* | | *-0.305* | | *-0.031* | | FOXO1 | | | ***0.000*** | | | ***-0.258*** | | | ***0.050*** | | ***-0.369*** | | | ***-0.146*** | |
| ***INS*** | ***0.014*** | ***0.149*** | ***0.050*** | ***0.037*** | ***0.262*** | INS | 0.837 | 0.009 | | | 0.045 | | -0.090 | | 0.109 | | INS | | | | 0.211 | | -0.162 | | | 0.093 | | -0.527 | | 0.202 | | |
| *PDX1* | 0.232 | -0.058 | 0.046 | -0.162 | 0.044 | PDX1 | *0.045* | *0.109* | | | *0.048* | | *0.003* | | *0.217* | | PDX1 | | | | 0.923 | | 0.005 | | | 0.048 | | -0.102 | | 0.112 | | |
| *NEUROD1* | 0.173 | 0.099 | 0.067 | -0.051 | 0.250 | NEUROD1 | 0.697 | -0.026 | | | 0.067 | | -0.176 | | 0.123 | | NEUROD1 | | | | 0.209 | | -0.154 | | | 0.115 | | -0.409 | | 0.101 | | |
| ***SLC16A1*** | ***0.000*** | ***-0.166*** | ***0.021*** | ***-0.214*** | ***-0.120*** | SLC16A1 | ***0.000*** | ***-0.354*** | | | ***0.022*** | | ***-0.403*** | | ***-0.305*** | | SLC16A1 | | | | ***0.000*** | | ***-0.278*** | | | ***0.046*** | | ***-0.380*** | | ***-0.176*** | | |
| *SYP* | 0.518 | -0.051 | 0.077 | -0.224 | 0.120 | SYP | 0.68 | 0.023 | | | 0.055 | | -0.099 | | 0.146 | | SYP | | | | 0.067 | | 0.115 | | | 0.056 | | -0.010 | | 0.241 | | |
| *GCK* | *0.034* | *0.304* | *0.124* | *0.029* | *0.580* | GCK | 0.62 | -0.063 | | | 0.124 | | -0.340 | | 0.213 | | GCK | | | | 0.793 | | -0.033 | | | 0.123 | | -0.308 | | 0.242 | | |
| *MAFA* | 0.275 | -0.118 | 0.103 | -0.347 | 0.110 | MAFA | 0.152 | -0.158 | | | 0.103 | | -0.388 | | 0.070 | | MAFA | | | | 0.181 | | -0.151 | | | 0.105 | | -0.384 | | 0.083 | | |
| ***NKX2.2*** | ***0.004*** | ***-0.221*** | ***0.060*** | ***-0.355*** | ***-0.088*** | NKX2.2 | 0.042 | -0.215 | | | 0.093 | | -0.423 | | -0.009 | | NKX2.2 | | | | 0.253 | | -0.073 | | | 0.060 | | -0.208 | | 0.061 | | |
| *LDHA* | *0.028* | *-0.122* | *0.048* | *-0.229* | *-0.016* | LDHA | 0.334 | 0.048 | | | 0.047 | | -0.057 | | 0.154 | | LDHA | | | | 0.994 | | 0.000 | | | 0.057 | | -0.129 | | 0.128 | | |
| *MAFB* | *0.042* | *-0.093* | *0.040* | *-0.182* | *-0.004* | MAFB | *0.027* | *-0.105* | | | *0.041* | | *-0.197* | | *-0.015* | | MAFB | | | | ***0.001*** | | ***-0.179*** | | | ***0.040*** | | ***-0.268*** | | ***-0.091*** | | |
| ***NKX6.1*** | ***0.003*** | ***-0.304*** | ***0.079*** | ***-0.480*** | ***-0.130*** | NKX6.1 | 0.62 | 0.041 | | | 0.082 | | -0.140 | | 0.224 | | NKX6.1 | | | | 0.714 | | -0.030 | | | 0.078 | | -0.204 | | 0.145 | | |
| *ARNT* | 0.086 | -0.124 | 0.065 | -0.269 | 0.021 | ARNT | 0.311 | 0.061 | | | 0.057 | | -0.067 | | 0.189 | | ARNT | | | | 0.611 | | 0.036 | | | 0.068 | | -0.116 | | 0.187 | | |
| *PAX4* | *0.021* | *0.368* | *0.134* | *0.069* | *0.667* | PAX4 | 0.316 | 0.141 | | | 0.134 | | -0.157 | | 0.439 | | PAX4 | | | | 0.300 | | 0.147 | | | 0.134 | | -0.152 | | 0.446 | | |
| *SST* | 0.182 | -0.143 | 0.100 | -0.366 | 0.079 | SST | 0.398 | 0.086 | | | 0.098 | | -0.132 | | 0.305 | | SST | | | | 0.952 | | 0.006 | | | 0.098 | | -0.212 | | 0.224 | | |
| *DDIT3* | 0.166 | -0.318 | 0.206 | -0.769 | 0.151 | DDIT3 | 0.956 | 0.012 | | | 0.213 | | -0.463 | | 0.487 | | DDIT3 | | | | 0.219 | | 0.270 | | | 0.206 | | -0.188 | | 0.728 | | |
| *GCG* | 0.135 | 0.412 | 0.254 | -0.153 | 0.979 | GCG | 0.172 | *-*0.372 | | | 0.253 | | -0.935 | | 0.191 | | GCG | | | | 0.278 | | -0.291 | | | 0.254 | | -0.856 | | 0.274 | | |
| *PAX6* | 0.469 | 0.055 | 0.074 | -0.109 | 0.220 | PAX6 | 0.625 | 0.037 | | | 0.075 | | -0.129 | | 0.205 | | PAX6 | | | | 0.314 | | 0.078 | | | 0.074 | | -0.086 | | 0.242 | | |
| ***MYC*** | ***0.003*** | ***-0.452*** | ***0.118*** | ***-0.715*** | ***-0.189*** | MYC | 0.84 | 0.028 | | | 0.136 | | -0.274 | | 0.330 | | MYC | | | | 0.066 | | -0.252 | | | 0.122 | | -0.525 | | 0.020 | | |
| *NANOG* | 0.112 | -0.183 | 0.105 | -0.418 | 0.051 | NANOG | 0.379 | *0.097* | | | 0.106 | | -0.139 | | 0.335 | | NANOG | | | | 0.372 | | 0.100 | | | 0.107 | | -0.138 | | 0.338 | | |
| ***SLC2A2*** | ***0.014*** | ***0.181*** | ***0.061*** | ***0.046*** | ***0.316*** | SLC2A2 | 0.41 | 0.061 | | | 0.072 | | -0.098 | | 0.222 | | SLC2A2 | | | | *0.031* | | *0.163* | | | *0.065* | | *0.018* | | *0.307* | | |
| ***HIF1A*** | ***0.008*** | ***-0.097*** | ***0.030*** | ***-0.164*** | ***-0.031*** | HIF1A | *0.024* | *-0.088* | | | *0.033* | | *-0.162* | | *-0.014* | | HIF1A | | | | 0.104 | | -0.158 | | | 0.060 | | -0.386 | | 0.071 | | |
| ***PTPN1*** | ***0.007*** | ***0.121*** | ***0.035*** | ***0.042*** | ***0.201*** | PTPN1 | 0.162 | 0.055 | | | 0.037 | | -0.026 | | 0.137 | | PTPN1 | | | | ***0.009*** | | ***0.123*** | | | ***0.038*** | | ***0.037*** | | ***0.208*** | | |
| *STK11* | 0.346 | -0.045 | 0.046 | -0.147 | 0.057 | STK11 | 0.229 | *-0.056* | | | 0.044 | | -0.154 | | 0.042 | | STK11 | | | | 0.145 | | 0.066 | | | 0.042 | | -0.027 | | 0.159 | | |
| ***ARX*** | 0.194 | -0.180 | 0.099 | -0.561 | 0.199 | ARX | ***0.001*** | ***-0.224*** | | | ***0.051*** | | ***-0.337*** | | ***-0.111*** | | ARX | | | | *0.041* | | *-0.125* | | | *0.053* | | *-0.244* | | *-0.006* | | |
| *SLC2A4* | 0.336 | 0.119 | 0.118 | -0.144 | 0.384 | SLC2A4 | 0.984 | -0.005 | | | 0.263 | | -1.033 | | 1.022 | | SLC2A4 | | | | *0.029* | | *-0.369* | | | *0.144* | | *-0.690* | | *-0.047* | | |
| *SOX9* | 0.086 | -0.591 | 0.310 | -1.282 | 0.100 | SOX9 | 0.233 | -0.396 | | | 0.312 | | -1.093 | | 0.299 | | SOX9 | | | | 0.974 | | 0.011 | | | 0.317 | | -0.695 | | 0.716 | | |
| *HES1* | 0.238 | -0.422 | 0.337 | -1.173 | 0.328 | HES1 | 0.611 | 0.177 | | | 0.338 | | -0.575 | | 0.931 | | HES1 | | | | 0.712 | | 0.128 | | | 0.338 | | -0.624 | | 0.880 | | |
| *NGN3* | ***0.14*** | ***-0.141*** | ***0.088*** | ***-0.338*** | ***0.055*** | NGN3 | 0.258 | 0.105 | | | 0.088 | | -0.090 | | 0.301 | | NGN3 | | | | *0.020* | | *0.239* | | | *0.087* | | *0.046* | | *0.432* | | |
| ***POU5F1*** | 0.102 | 0.093 | 0.052 | -0.022 | 0.208 | POU5F1 | *0.049* | *-0.072* | | | *0.032* | | *-0.146* | | *0.000* | | POU5F1 | | | | ***0.010*** | | ***-0.096*** | | | ***0.030*** | | ***-0.164*** | | ***-0.029*** | | |

**Supplementary table S2: Effects of tunicamycin on total gene expression.** The p values for statistical significance as determined by STUDENT INDEPENDENT T-TEST, mean logged fold change in expression, standard errors of difference (SED) and the 95% confidence intervals are given below. Gene list is a priori

| **Gene** | **P value** | **Mean diff.** | **SED** | **95% CI** | |
| --- | --- | --- | --- | --- | --- |
|  |  |  |  | **Lower** | **Upper** |
| *ARNT* | 0.642 | 0.017 | 0.036 | -0.081 | 0.117 |
| ***ARX*** | ***0.033*** | ***0.662*** | ***0.208*** | ***0.085*** | ***1.239*** |
| ***DDIT3*** | ***0.033*** | ***0.387*** | ***0.121*** | ***0.052*** | ***0.722*** |
| ***FOXO1*** | ***0.013*** | ***-0.216*** | ***0.051*** | ***-0.357*** | ***-0.075*** |
| *GCG* | 0.931 | -0.025 | 0.272 | -0.781 | 0.730 |
| *GCK* | 0.081 | -0.174 | 0.075 | -0.384 | 0.035 |
| *HHEX* | 0.115 | 0.499 | 0.248 | -0.190 | 1.189 |
| *INS* | 0.448 | -0.041 | 0.05 | -0.180 | 0.096 |
| *LDHA* | 0.226 | 0.028 | 0.02 | -0.027 | 0.083 |
| ***MAFA*** | ***0.050*** | ***-0.319*** | ***0.115*** | ***-0.640*** | ***0.001*** |
| *MAFB* | 0.085 | -0.086 | 0.038 | -0.193 | 0.019 |
| *NANOG* | 0.274 | -0.068 | 0.054 | -0.217 | 0.081 |
| *ND1* | 0.857 | -0.008 | 0.044 | -0.130 | 0.113 |
| ***NKX2-2*** | ***0.005*** | ***0.100*** | ***0.018*** | ***0.049*** | ***0.152*** |
| *NKX6-1* | 0.099 | 0.174 | 0.06 | -0.080 | 0.429 |
| ***PAX4*** | ***0.049*** | ***-0.345*** | ***0.123*** | ***-0.689*** | **-0.003** |
| *PAX6* | 0.161 | -0.144 | 0.084 | -0.379 | 0.089 |
| *PDX1* | 0.058 | -0.216 | 0.082 | -0.444 | 0.012 |
| *POU5F1* | 0.639 | -0.068 | 0.136 | -0.447 | 0.309 |
| *SLC16A1* | 0.351 | -0.053 | 0.051 | -0.195 | 0.088 |
| *SOX9* | 0.145 | 0.393 | 0.218 | -0.211 | 0.998 |
| *SST* | 0.111 | 0.325 | 0.16 | -0.118 | 0.770 |
| *STK11* | 0.165 | -0.129 | 0.077 | -0.343 | 0.083 |
| *SYP* | 0.056 | -0.110 | 0.041 | -0.226 | 0.005 |

**Supplementary table S3: Effects of cell insult treatments on splicing factor expression.** The p values for statistical significance as determined by STUDENT INDEPENDENT T-TEST, mean logged fold change in expression, standard errors of difference (SED) and the 95% confidence intervals are given below. Gene list is a priori, but results reaching Bonferroni significance for 5 treatments (p = 0.01) are indicated in bold italic type. Nominally significant results are indicated by underlined italic text. Bonferroni corrections were performed to take account of the number of treatments but not for the splicing regulators as these are known to be affected by cell stresses in a number of other cell types (16).

| **25 mM high glucose Independent Samples Test p=0.01** | | | | | | |
| --- | --- | --- | --- | --- | --- | --- |
|  | Sig. (2-tailed) | Mean Difference | Std. Error Difference | 95% CI | | |
|  |  |  |  | Lower | | Upper |
| *AKAP17A* | 0.538 | 0.035 | 0.052 | 0.109 | | -0.179 |
| *HNRNPA0* | 0.816 | -0.016 | 0.063 | -0.189 | | 0.158 |
| *HNRNPA1* | 0.872 | 0.004 | 0.021 | -0.055 | | 0.062 |
| ***HNRNPA2B1*** | ***0.006*** | ***0.176*** | ***0.033*** | ***0.084*** | | ***0.268*** |
| *HNRNPD* | 0.667 | 0.083 | 0.180 | -0.416 | | 0.583 |
| *HNRNPH3* | 0.243 | 0.086 | 0.063 | -0.089 | | 0.262 |
| *HNRNPK* | 0.800 | -0.014 | 0.054 | -0.163 | | 0.134 |
| *HNRNPM* | 0.790 | 0.038 | 0.135 | -0.337 | | 0.414 |
| *HNRNPUL2* | 0.586 | 0.030 | 0.050 | -0.110 | | 0.170 |
| *IMP3* | 0.364 | 0.064 | 0.063 | -0.110 | | 0.239 |
| *LSM14A* | 0.461 | 0.050 | 0.061 | -0.120 | | 0.219 |
| *LSM2* | 0.503 | -0.445 | 0.605 | -2.124 | | 1.234 |
| *PNISR* | 0.062 | 0.114 | 0.044 | -0.009 | | 0.237 |
| *SF3B1* | 0.444 | 0.069 | 0.081 | -0.156 | | 0.294 |
| *SRSF1* | 0.537 | 0.025 | 0.037 | -0.078 | | 0.129 |
| *SRSF2* | 0.052 | 0.142 | 0.052 | -0.002 | | 0.286 |
| *SRSF3* | 0.117 | 0.090 | 0.045 | -0.035 | | 0.216 |
| *SRSF6* | 0.653 | -0.020 | 0.040 | -0.132 | | 0.093 |
| *SRSF7* | 0.280 | -0.079 | 0.063 | -0.253 | | 0.096 |
| *TRA2B* | 0.154 | 0.135 | 0.077 | -0.079 | | 0.348 |
| **2.5 mM low glucose Independent Samples Test p=0.01** | | | | | | |
|  | Sig. (2-tailed) | Mean Difference | Std. Error Difference | 95% CI | | |
|  |  |  |  | Lower | | Upper |
| *AKAP17A* | *0.029* | *0.159* | *0.048* | *0.026* | | *0.291* |
| *HNRNPA0* | *0.022* | *0.103* | *0.028* | *0.024* | | *0.181* |
| *HNRNPA1* | 0.242 | 0.159 | 0.098 | -0.250 | | 0.567 |
| ***HNRNPA2B1*** | ***0.004*** | ***0.179*** | ***0.030*** | ***0.097*** | | ***0.262*** |
| *HNRNPD* | *0.047* | *-0.374* | *0.132* | *-0.741* | | *-0.007* |
| *HNRNPH3* | 0.154 | 0.105 | 0.060 | -0.061 | | 0.271 |
| *HNRNPK* | 0.063 | 0.069 | 0.027 | -0.006 | | 0.144 |
| *HNRNPM* | 0.879 | 0.022 | 0.128 | -0.513 | | 0.557 |
| *HNRNPUL2* | 0.471 | 0.022 | 0.027 | -0.054 | | 0.097 |
| *IMP3* | 0.217 | 0.028 | 0.019 | -0.025 | | 0.081 |
| ***LSM14A*** | ***0.004*** | ***0.109*** | ***0.019*** | ***0.057*** | | ***0.160*** |
| *LSM2* | 0.290 | -0.712 | 0.505 | -2.812 | | 1.388 |
| ***PNISR*** | ***0.005*** | ***0.202*** | ***0.036*** | ***0.100*** | | ***0.303*** |
| *SF3B1* | *0.047* | *0.202* | *0.071* | *0.005* | | *0.399* |
| ***SRSF1*** | ***0.001*** | ***0.107*** | ***0.012*** | ***0.072*** | | ***0.141*** |
| *SRSF2* | *0.015* | *0.285* | *0.069* | *0.093* | | *0.476* |
| ***SRSF3*** | ***0.009*** | ***0.141*** | ***0.030*** | ***0.059*** | | ***0.223*** |
| ***SRSF6*** | ***0.001*** | ***0.201*** | ***0.021*** | ***0.143*** | | ***0.259*** |
| *SRSF7* | 0.667 | 0.026 | 0.055 | -0.128 | | 0.179 |
| *TRA2B* | 0.776 | 0.016 | 0.052 | -0.128 | | 0.159 |
| **Hypoxia Independent Samples Test p=0.01** | | | | | | |
|  | Sig. (2-tailed) | Mean Difference | Std. Error Difference | | 95% CI | |
|  |  |  |  |  | Lower | Upper |
| *AKAP17A* | 0.034 | 0.190 | 0.060 | | 0.023 | 0.357 |
| *HNRNPA0* | 0.291 | 0.029 | 0.024 | | -0.038 | 0.096 |
| *HNRNPA1* | 0.011 | -0.098 | 0.022 | | -0.158 | -0.038 |
| ***HNRNPA2B1*** | **0.004** | **0.120** | **0.020** | | **0.064** | **0.175** |
| *HNRNPD* | 0.140 | -0.367 | 0.200 | | -0.921 | 0.187 |
| *HNRNPH3* | 0.098 | 0.126 | 0.059 | | -0.037 | 0.289 |
| *HNRNPK* | 0.056 | 0.071 | 0.027 | | -0.003 | 0.146 |
| *HNRNPM* | 0.884 | 0.021 | 0.132 | | -0.345 | 0.386 |
| *HNRNPUL2* | 0.564 | 0.017 | 0.027 | | -0.058 | 0.092 |
| *IMP3* | 0.274 | 0.021 | 0.016 | | -0.025 | 0.067 |
| ***LSM14A*** | **0.004** | **0.139** | **0.023** | | **0.076** | **0.201** |
| *LSM2* | 0.252 | -0.794 | 0.508 | | -2.861 | 1.274 |
| ***PNISR*** | **0.007** | **0.234** | **0.046** | | **0.107** | **0.362** |
| *SF3B1* | 0.018 | 0.201 | 0.052 | | 0.058 | 0.345 |
| *SRSF1* | 0.024 | 0.056 | 0.016 | | 0.012 | 0.100 |
| *SRSF2* | 0.094 | 0.245 | 0.112 | | -0.066 | 0.555 |
| ***SRSF3*** | **0.001** | **0.160** | **0.018** | | **0.111** | **0.209** |
| ***SRSF6*** | **0.003** | **0.190** | **0.030** | | **0.106** | **0.275** |
| *SRSF7* | 0.994 | -0.000 | 0.054 | | -0.151 | 0.150 |
| *TRA2B* | 0.715 | -0.020 | 0.051 | | -0.162 | 0.122 |
| **Lipotoxicity Independent Samples Test p=0.01** | | | | | | |
|  | Sig. (2-tailed) | Mean Difference | Std. Error Difference | | 95% CI | |
|  |  |  |  |  | Lower | Upper |
| *AKAP17A* | 0.127 | 0.126 | 0.066 | | -0.056 | 0.309 |
| *HNRNPA0* | 0.288 | -0.051 | 0.042 | | -0.167 | 0.065 |
| *HNRNPA1* | 0.065 | -0.039 | 0.016 | | -0.083 | 0.004 |
| *HNRNPA2B1* | 0.824 | -0.014 | 0.060 | | -0.179 | 0.151 |
| *HNRNPD* | 0.749 | -0.074 | 0.214 | | -0.669 | 0.522 |
| *HNRNPH3* | 0.146 | 0.119 | 0.066 | | -0.064 | 0.302 |
| *HNRNPK* | 0.749 | 0.011 | 0.033 | | -0.081 | 0.104 |
| *HNRNPM* | 0.967 | 0.006 | 0.133 | | -0.364 | 0.376 |
| *HNRNPUL2* | 0.453 | 0.025 | 0.030 | | -0.058 | 0.107 |
| ***IMP3*** | **0.006** | **0.084** | **0.016** | | **0.039** | **0.128** |
| *LSM14A* | 0.668 | 0.012 | 0.026 | | -0.060 | 0.084 |
| *LSM2* | 0.438 | 0.533 | 0.620 | | -1.189 | 2.256 |
| *PNISR* | 0.277 | -0.046 | 0.037 | | -0.148 | 0.056 |
| *SF3B1* | 0.270 | -0.088 | 0.069 | | -0.279 | 0.103 |
| *SRSF1* | 0.743 | -0.005 | 0.013 | | -0.040 | 0.031 |
| *SRSF2* | 0.639 | -0.033 | 0.064 | | -0.211 | 0.146 |
| *SRSF3* | 0.034 | -0.096 | 0.031 | | -0.181 | -0.012 |
| *SRSF6* | 0.762 | 0.012 | 0.036 | | -0.089 | 0.112 |
| *SRSF7* | 0.837 | -0.013 | 0.059 | | -0.177 | 0.151 |
| *TRA2B* | 0.118 | -0.106 | 0.053 | | -0.254 | 0.042 |
| **Cytokine assay Independent Samples Test *p*=0.01** | | | | | | |
|  | Sig. (2-tailed) | Mean Difference | Std. Error Difference | | 95% CI | |
|  |  |  |  |  | Lower | Upper |
| ***AKAP17A*** | **0.002** | **0.240** | **0.032** | | **0.151** | **0.329** |
| *HNRNPA0* | 0.264 | 0.038 | 0.030 | | -0.044 | 0.120 |
| *HNRNPA1* | 0.162 | 0.025 | 0.015 | | -0.016 | 0.066 |
| ***HNRNPA2B1*** | **0.001** | **0.217** | **0.024** | | **0.151** | **0.282** |
| *HNRNPD* | 0.056 | -0.364 | 0.136 | | -0.743 | 0.014 |
| *HNRNPH3* | 0.053 | 0.143 | 0.053 | | -0.003 | 0.289 |
| *HNRNPK* | 0.033 | 0.087 | 0.027 | | 0.012 | 0.163 |
| *HNRNPM* | 0.179 | 0.221 | 0.136 | | -0.156 | 0.597 |
| *HNRNPUL2* | 0.656 | -0.013 | 0.027 | | -0.087 | 0.061 |
| *IMP3* | 0.736 | -0.004 | 0.011 | | -0.035 | 0.027 |
| ***LSM14A*** | **0.003** | **0.118** | **0.018** | | **0.068** | **0.167** |
| *LSM2* | 0.253 | -0.790 | 0.508 | | -2.860 | 1.281 |
| ***PNISR*** | **0.003** | **0.243** | **0.039** | | **0.135** | **0.351** |
| *SF3B1* | 0.030 | 0.168 | 0.051 | | 0.026 | 0.309 |
| ***SRSF1*** | **<0.001** | **0.117** | **0.009** | | **0.093** | **0.141** |
| ***SRSF2*** | **0.006** | **0.274** | **0.051** | | **0.134** | **0.414** |
| ***SRSF3*** | **0.002** | **0.185** | **0.027** | | **0.110** | **0.260** |
| *SRSF6* | 0.113 | 0.058 | 0.029 | | -0.021 | 0.137 |
| *SRSF7* | 0.370 | -0.046 | 0.046 | | -0.172 | 0.081 |
| *TRA2B* | 0.867 | 0.009 | 0.050 | | -0.131 | 0.149 |

**Supplementary table S4: Effects of cell insult treatments on alternatively spliced gene expression.** The p values for statistical significance as determined by STUDENT INDEPENDENT T-TEST, mean logged fold change in expression, standard errors of difference (SED) and the 95% confidence intervals are given below. Gene list is a priori, but results reaching Bonferroni significance for 3 time points (p = 0.017) are indicated in bold italic type. Nominally significant results are indicated by underlined italic text. Bonferroni corrections were performed to take account of the number of timepoints but not for the genes in the target panel as these are á priori.

| **25 mM high glucose Independent Samples Test *p*=0.016** | | | | | | | | | | | | | | | | | | | |
| --- | --- | --- | --- | --- | --- | --- | --- | --- | --- | --- | --- | --- | --- | --- | --- | --- | --- | --- | --- |
|  | *p* value | Mean Diff. | SED | 95% CI | |  | *p* value | Mean diff | SED | 95% CI | |  | *p* value | Mean diff. | SED | | 95% CI | | |
|  |  |  |  | Lower | Upper |  |  |  |  | Lower | Upper |  |  |  |  |  | Lower | | Upper |
| *INS* | 0.619 | -.0854 | 0.149 | -0.493 | 0.664 | *INS* | 0.184 | 0.139 | 0.098 | -0.357 | 0.078 | *INS* | 0.167 | -0.122 | 0.082 | | -0.061 | | 0.306 |
| *NANOG* | 0.571 | -0.653 | 0.111 | -0.183 | 0.314 | *NANOG* | 0.962 | 0.005 | 0.107 | -0.244 | 0.234 | *NANOG* | 0.595 | -0.059 | 0.108 | | -0.181 | | 0.300 |
| ***PAX6*** | ***0.007*** | ***0.494*** | ***0.147*** | ***-0.822*** | ***-0.166*** | *PAX6* | ***0.002*** | ***-0.357*** | ***0.082*** | ***0.169*** | ***0.546*** | *PAX6* | ***0.003*** | ***1.278*** | ***0.332*** | | ***-2.018*** | | ***-0.539*** |
| *PTPN1* | *0.045* | *0.398* | *0.174* | *-0.787* | *-0.011* | ***PTPN1*** | ***0.011*** | ***-0.417*** | ***0.125*** | ***0.126*** | ***0.708*** | *PTPN1* | ***0.003*** | ***0.974*** | ***0.256*** | | ***-1.545*** | | ***-0.404*** |
| ***POU5F1*** | ***0.004*** | ***0.277*** | ***0.075*** | ***-0.445*** | ***-0.111*** | ***POU5F1*** | ***0.014*** | ***-0.173*** | ***0.058*** | ***0.044*** | ***0.303*** | *POU5F1* | 0.139 | -0.090 | 0.056 | | -0.035 | | 0.215 |
| **2.5 mM low glucose Independent Samples Test *p*=0.016** | | | | | | | | | | | | | | | | | | | |
|  | *p* value | Mean Diff. | SED | 95% CI | |  | *p* value | Mean diff. | SED | 95% CI | |  | *p* value | Mean diff. | SED | | 95% CI | | |
|  |  |  |  | Lower | Upper |  |  |  |  | Lower | Upper |  |  |  |  |  | Lower | | Upper |
| *INS* | 0.135 | -0.196 | 0.121 | -0.073 | 0.465 | *INS* | 0.631 | -0.086 | 0.156 | -0.524 | 0.697 | *INS* | 0.631 | -0.179 | 0.156 | | -0.524 | | 0.697 |
| *NANOG* | 0.728 | -0.038 | 0.108 | -0.201 | 0.279 | *NANOG* | 0.694 | 0.045 | 0.111 | -0.293 | 0.203 | *NANOG* | 0.694 | -0.128 | 0.111 | | -0.293 | | 0.203 |
| *PAX6* | 0.895 | 0.027 | 0.202 | -0.477 | 0.423 | *PAX6* | 0.920 | 0.015 | 0.148 | -0.345 | 0.315 | *PAX6* | 0.920 | 2.090 | 0.148 | | -0.345 | | 0.315 |
| *PTPN1* | 0.072 | 0.379 | 0.189 | -0.800 | 0.041 | *PTPN1* | 0.821 | -0.040 | 0.173 | -0.345 | 0.426 | *PTPN1* | 0.821 | 0.442 | 0.173 | | -0.345 | | 0.426 |
| *POU5F1* | 0.304 | 0.202 | 0.151 | -0.807 | 0.403 | *POU5F1* | ***0.005*** | ***0.238*** | ***0.066*** | ***-0.385*** | ***-0.093*** | *POU5F1* | ***0.005*** | ***0.017*** | ***0.066*** | | ***-0.385*** | | ***-0.093*** |
| **<3% O2 Hypoxic conditions Independent Samples Test *p*=0.016** | | | | | | | | | | | | | | | | | | | |
|  | *p* value | Mean diff. | SED | 95% CI | |  | *p* value | Mean diff. | SED | 95% CI | |  | P value | Mean diff. | SED | 95% CI | | | |
|  |  |  |  | Lower | Upper |  |  |  |  | Lower | Upper |  |  |  |  | Lower | | Upper | |
| ***INS*** | *0.042* | *-0.117* | *0.052* | *-0.230* | *-0.005* | *INS* | 0.624 | 0.030 | 0.061 | -0.101 | 0.162 | ***INS*** | ***0.011*** | ***-0.264*** | ***0.084*** | ***-0.451*** | | ***-0.077*** | |
| *NANOG* | 0.955 | -0.004 | 0.085 | -0.187 | 0.178 | *NANOG* | 0.538 | 0.051 | 0.081 | -0.124 | 0.227 | *NANOG* | 0.424 | -0.094 | 0.113 | -0.346 | | 0.158 | |
| *PAX6* | 0.357 | 0.073 | 0.077 | -0.093 | 0.241 | *PAX6* | 0.901 | 0.010 | 0.083 | -0.169 | 0.190 | *PAX6* | 0.510 | -0.056 | 0.083 | -0.242 | | 0.128 | |
| ***PTPN1*** | 0.784 | 0.049 | 0.174 | -0.378 | 0.477 | *PTPN1* | 0.582 | -0.079 | 0.141 | -0.384 | 0.225 | *PTPN1* | ***0.005*** | ***0.335*** | ***0.092*** | ***0.129*** | | ***0.541*** | |
| ***POU5F1*** | ***0.007*** | ***-0.317*** | ***0.075*** | ***-0.504*** | ***-0.130*** | *POU5F1* | 0.112 | -0.127 | 0.056 | -0.249 | -0.006 | *POU5F1* | 0.029 | 0.179 | 0.070 | 0.023 | | 0.336 | |

| **0.5 mM Palmitic Acid Independent Samples Test *p*=0.016** | | | | | | | | | | | | | | | | | |
| --- | --- | --- | --- | --- | --- | --- | --- | --- | --- | --- | --- | --- | --- | --- | --- | --- | --- |
|  | *p* value | Mean diff. | SED | 95% CI | |  | *p value* | Mean diff | SED | 95% CI | |  | *p* value | Mean diff. | SED | 95% CI | |
|  |  |  |  | Lower | Upper |  |  |  |  | Lower | Upper |  |  |  |  | Lower | Upper |
| *INS* | *0.036* | *0.091* | *0.038* | *-0.175* | *-0.007* | *INS* | 0.840 | 0.007 | 0.035 | -0.085 | 0.071 | *INS* | 0.904 | 0.015 | 0.117 | -0.498 | 0.466 |
| *NANOG* | 0.455 | -0.244 | 0.314 | -0.456 | 0.945 | *NANOG* | 0.313 | -0.334 | 3.147 | -0.367 | 0.103 | *NANOG* | 0.509 | -0.216 | 0.315 | -0.487 | 0.919 |
| ***PAX6*** | *0.015* | *0.344* | *0.118* | *-0.606* | *-0.082* | *PAX6* | 0.914 | -0.128 | 0.117 | -0.247 | 0.273 | *PAX6* | ***0.006*** | ***0.426*** | ***0.124*** | ***-0.702*** | ***-0.150*** |
| *PTPN1* | 0.614 | -0.068 | 0.131 | -0.224 | 0.362 | *PTPN1* | 0.308 | -0.802 | 0.773 | -0.896 | 0.225 | *PTPN1* | 0.380 | -0.684 | 0.738 | -0.101 | 0.238 |
| ***POU5F1*** | 0.264 | 0.230 | 0.195 | -0.665 | 0.204 | *POU5F1* | 0.168 | -0.288 | 0.194 | -0.144 | 0.720 | *POU5F1* | ***0.002*** | ***-0.787*** | ***0.195*** | ***0.352*** | ***1.223*** |
| **Pro-inflammatory Cytokines Independent Samples Test *p*=0.016** | | | | | | | | | | | | | | | | | |
|  | *p* value | Mean diff. | SED | 95% CI | |  | *p* value | Mean diff. | SED | 95% CI | |  | *p* value | Mean diff. | SED | 95% CI | |
|  |  |  |  | Lower | Upper |  |  |  |  | Lower | Upper |  |  |  |  | Lower | Upper |
| *INS* | 0.945 | -0.003 | 0.054 | -0.117 | 0.125 | *INS* | 0.764 | 0.0143 | 0.046 | -0.118 | 0.089 | *INS* | 0.083 | 0.084 | 0.044 | -0.182 | 0.013 |
| *NANOG* | 0.138 | -0.164 | 0.102 | -0.063 | 0.391 | *NANOG* | 0.212 | 0.138 | 0.104 | -0.371 | 0.093 | *NANOG* | 0.279 | 0.123 | 0.108 | -0.364 | 0.117 |
| *PAX6* | 0.527 | -0.340 | 0.519 | -0.815 | 1.496 | *PAX6* | 0.529 | -0.347 | 0.533 | -0.841 | 1.536 | *PAX6* | 0.211 | -0.675 | 0.505 | -0.450 | 1.800 |
| *PTPN1* | 0.379 | -0.356 | 7.366 | -10.125 | 23.844 | *PTPN1* | 0.434 | 0.188 | 0.231 | -0.703 | 0.326 | *PTPN1* | 0.218 | -0.305 | 0.232 | -0.212 | 0.824 |
| ***POU5F1*** | 0.508 | -0.242 | 0.352 | -0.543 | 1.028 | ***POU5F1*** | ***0.011*** | ***1.135*** | ***0.363*** | ***-1.943*** | ***-0.327*** | *POU5F1* | ***0.002*** | ***1.426*** | ***0.354*** | ***-2.216*** | ***-0.637*** |

**Supplementary table S5: GO pathway analysis for genes demonstrating dysregulated splicing in islets from donors with T2D**

| **GO Molecular function** | **Overlap** | **P-value** | **Adjusted P-value** |
| --- | --- | --- | --- |
| RNA binding (GO:0003723) | 597/1388 | 1.06E-22 | 1.16E-19 |
| protein serine/threonine kinase activity (GO:0004674) | 173/369 | 8.77E-11 | 4.80E-08 |
| cadherin binding (GO:0045296) | 151/314 | 1.41E-10 | 5.14E-08 |
| protein kinase activity (GO:0004672) | 224/514 | 8.48E-10 | 2.32E-07 |
| kinase activity (GO:0016301) | 131/281 | 2.50E-08 | 5.47E-06 |
| phosphotransferase activity, alcohol group as acceptor (GO:0016773) | 119/255 | 9.98E-08 | 1.82E-05 |
| adenyl ribonucleotide binding (GO:0032559) | 126/280 | 5.20E-07 | 8.13E-05 |
| Ras GTPase binding (GO:0017016) | 89/185 | 7.82E-07 | 1.07E-04 |
| mRNA binding (GO:0003729) | 86/180 | 1.70E-06 | 2.07E-04 |
| phosphatidylinositol binding (GO:0035091) | 54/101 | 2.19E-06 | 2.40E-04 |
| GTPase regulator activity (GO:0030695) | 121/276 | 4.29E-06 | 4.27E-04 |
| ATP binding (GO:0005524) | 113/256 | 5.97E-06 | 0.001 |
| Rab GTPase binding (GO:0017137) | 65/131 | 6.37E-06 | 0.001 |
| GTPase activator activity (GO:0005096) | 109/250 | 1.67E-05 | 0.001 |
| DNA-dependent ATPase activity (GO:0008094) | 40/73 | 2.06E-05 | 0.002 |
| purine ribonucleoside triphosphate binding (GO:0035639) | 161/397 | 3.02E-05 | 0.002 |
| protein kinase binding (GO:0019901) | 196/496 | 2.92E-05 | 0.002 |
| phosphatidylinositol phosphate binding (GO:1901981) | 43/82 | 4.27E-05 | 0.003 |
| ATP-dependent helicase activity (GO:0008026) | 44/86 | 7.57E-05 | 0.004 |
| RNA-dependent ATPase activity (GO:0008186) | 37/69 | 7.72E-05 | 0.004 |
| 1-phosphatidylinositol binding (GO:0005545) | 12/15 | 1.30E-04 | 0.006 |
| RNA helicase activity (GO:0003724) | 36/68 | 1.36E-04 | 0.006 |
| aminoacyl-tRNA ligase activity (GO:0004812) | 25/42 | 1.23E-04 | 0.006 |
| ATP-dependent RNA helicase activity (GO:0004004) | 36/68 | 1.36E-04 | 0.006 |
| transition metal ion transmembrane transporter activity (GO:0046915) | 21/33 | 1.12E-04 | 0.006 |
| ATPase activity, coupled (GO:0042623) | 47/95 | 1.27E-04 | 0.006 |
| ubiquitin-like protein-specific protease activity (GO:0019783) | 39/75 | 1.21E-04 | 0.006 |
| kinase binding (GO:0019900) | 165/419 | 1.47E-04 | 0.006 |
| phosphatase binding (GO:0019902) | 49/103 | 3.07E-04 | 0.012 |
| actin binding (GO:0003779) | 105/255 | 3.51E-04 | 0.013 |
| repressing transcription factor binding (GO:0070491) | 29/54 | 4.33E-04 | 0.015 |
| actin filament binding (GO:0051015) | 58/128 | 4.55E-04 | 0.016 |
| mannosyltransferase activity (GO:0000030) | 16/25 | 0.001 | 0.023 |
| thiol-dependent ubiquitinyl hydrolase activity (GO:0036459) | 44/93 | 0.001 | 0.023 |
| ATPase activity, coupled to transmembrane movement of ions, phosphorylative mechanism (GO:0015662) | 15/23 | 0.001 | 0.023 |
| translation factor activity, RNA binding (GO:0008135) | 34/68 | 0.001 | 0.025 |
| protein homodimerization activity (GO:0042803) | 244/665 | 0.001 | 0.025 |
| divalent inorganic cation transmembrane transporter activity (GO:0072509) | 22/39 | 0.001 | 0.025 |
| poly(U) RNA binding (GO:0008266) | Dec-17 | 0.001 | 0.026 |
| mitogen-activated protein kinase kinase binding (GO:0031434) | 33/66 | 0.001 | 0.027 |
| MAP kinase kinase kinase activity (GO:0004709) | 38/79 | 0.001 | 0.028 |
| phosphoprotein phosphatase activity (GO:0004721) | 60/137 | 0.001 | 0.028 |
| small GTPase binding (GO:0031267) | 32/64 | 0.001 | 0.029 |
| ubiquitin-like protein ligase activity (GO:0061659) | 78/187 | 0.001 | 0.030 |
| Rab guanyl-nucleotide exchange factor activity (GO:0017112) | 27/52 | 0.001 | 0.032 |
| ATPase activity, coupled to movement of substances (GO:0043492) | 29/57 | 0.001 | 0.032 |
| phosphatidylinositol-3,4,5-trisphosphate binding (GO:0005547) | 19/33 | 0.001 | 0.033 |
| double-stranded RNA binding (GO:0003725) | 30/60 | 0.002 | 0.036 |
| Rho guanyl-nucleotide exchange factor activity (GO:0005089) | 30/60 | 0.002 | 0.036 |
| hydrolase activity, acting on acid anhydrides, catalyzing transmembrane movement of substances (GO:0016820) | 23/43 | 0.002 | 0.038 |
| protein deacetylase activity (GO:0033558) | 23/43 | 0.002 | 0.038 |
| ATP-dependent microtubule motor activity, minus-end-directed (GO:0008569) | Dec-18 | 0.002 | 0.041 |
| ubiquitin protein ligase activity (GO:0061630) | 79/193 | 0.002 | 0.043 |
| RNA polymerase II carboxy-terminal domain kinase activity (GO:0008353) | Nov-16 | 0.002 | 0.043 |
| histone deacetylase activity (GO:0004407) | 22/41 | 0.002 | 0.043 |
| cysteine-type peptidase activity (GO:0008234) | 32/66 | 0.002 | 0.043 |
| mRNA 3'-UTR binding (GO:0003730) | 31/64 | 0.003 | 0.049 |
| GTPase binding (GO:0051020) | 36/77 | 0.003 | 0.049 |
| thiol-dependent ubiquitin-specific protease activity (GO:0004843) | 36/77 | 0.003 | 0.049 |
| Rac GTPase binding (GO:0048365) | 23/44 | 0.003 | 0.049 |
| **GO Biological Processes** | **Overlap** | **P-value** | **Adjusted P-value** |
| protein phosphorylation (GO:0006468) | 212/471 | 7.71E-11 | 3.82E-07 |
| phosphorylation (GO:0016310) | 178/387 | 3.19E-10 | 7.92E-07 |
| regulation of translation (GO:0006417) | 109/214 | 8.56E-10 | 1.41E-06 |
| vesicle-mediated transport (GO:0016192) | 185/411 | 1.23E-09 | 1.52E-06 |
| RNA metabolic process (GO:0016070) | 98/192 | 5.17E-09 | 5.13E-06 |
| mRNA processing (GO:0006397) | 132/284 | 2.77E-08 | 2.29E-05 |
| transmembrane receptor protein tyrosine kinase signaling pathway (GO:0007169) | 173/397 | 6.97E-08 | 4.94E-05 |
| nucleotide-excision repair (GO:0006289) | 59/109 | 4.35E-07 | 2.40E-04 |
| peptidyl-lysine modification (GO:0018205) | 62/116 | 4.06E-07 | 2.40E-04 |
| chromatin remodeling (GO:0006338) | 62/117 | 6.04E-07 | 2.99E-04 |
| nuclear envelope disassembly (GO:0051081) | 31/47 | 8.48E-07 | 3.82E-04 |
| protein sumoylation (GO:0016925) | 41/69 | 9.85E-07 | 3.91E-04 |
| endosomal transport (GO:0016197) | 106/230 | 1.02E-06 | 3.91E-04 |
| cytosolic transport (GO:0016482) | 62/119 | 1.30E-06 | 4.29E-04 |
| regulation of small GTPase mediated signal transduction (GO:0051056) | 71/141 | 1.22E-06 | 4.29E-04 |
| cellular protein modification process (GO:0006464) | 379/1002 | 1.47E-06 | 4.56E-04 |
| regulation of gene silencing by miRNA (GO:0060964) | 40/68 | 1.91E-06 | 0.001 |
| mRNA splicing, via spliceosome (GO:0000398) | 117/262 | 2.07E-06 | 0.001 |
| mitotic nuclear envelope disassembly (GO:0007077) | 29/45 | 3.88E-06 | 0.001 |
| regulation of gene silencing by RNA (GO:0060966) | 34/56 | 4.18E-06 | 0.001 |
| regulation of posttranscriptional gene silencing (GO:0060147) | 34/56 | 4.18E-06 | 0.001 |
| cytoskeleton organization (GO:0007010) | 64/127 | 3.87E-06 | 0.001 |
| tRNA aminoacylation (GO:0043039) | 27/41 | 4.55E-06 | 0.001 |
| peptidyl-serine phosphorylation (GO:0018105) | 71/146 | 6.07E-06 | 0.001 |
| protein complex assembly (GO:0006461) | 88/190 | 6.38E-06 | 0.001 |
| lysosomal transport (GO:0007041) | 41/73 | 7.21E-06 | 0.001 |
| vascular endothelial growth factor receptor signaling pathway (GO:0048010) | 40/71 | 8.39E-06 | 0.002 |
| RNA processing (GO:0006396) | 89/194 | 9.08E-06 | 0.002 |
| peptidyl-serine modification (GO:0018209) | 80/171 | 1.04E-05 | 0.002 |
| RNA splicing, via transesterification reactions with bulged adenosine as nucleophile (GO:0000377) | 105/237 | 1.03E-05 | 0.002 |
| DNA repair (GO:0006281) | 124/289 | 1.17E-05 | 0.002 |
| regulation of glycolytic process (GO:0006110) | 34/58 | 1.24E-05 | 0.002 |
| mRNA export from nucleus (GO:0006406) | 54/106 | 1.44E-05 | 0.002 |
| Golgi vesicle transport (GO:0048193) | 117/272 | 1.78E-05 | 0.003 |
| protein autophosphorylation (GO:0046777) | 81/176 | 1.94E-05 | 0.003 |
| gene expression (GO:0010467) | 167/412 | 2.24E-05 | 0.003 |
| mRNA transport (GO:0051028) | 53/105 | 2.40E-05 | 0.003 |
| DNA metabolic process (GO:0006259) | 132/315 | 2.52E-05 | 0.003 |
| protein modification by small protein conjugation (GO:0032446) | 162/399 | 2.66E-05 | 0.003 |
| positive regulation of cellular amide metabolic process (GO:0034250) | 35/62 | 2.86E-05 | 0.004 |
| cellular response to DNA damage stimulus (GO:0006974) | 137/330 | 3.09E-05 | 0.004 |
| mRNA-containing ribonucleoprotein complex export from nucleus (GO:0071427) | 51/101 | 3.34E-05 | 0.004 |
| cellular response to insulin stimulus (GO:0032869) | 55/111 | 3.34E-05 | 0.004 |
| tRNA aminoacylation for protein translation (GO:0006418) | 28/47 | 4.78E-05 | 0.005 |
| positive regulation of phosphorylation (GO:0042327) | 92/209 | 4.74E-05 | 0.005 |
| tRNA export from nucleus (GO:0006409) | 22/34 | 5.28E-05 | 0.005 |
| tRNA-containing ribonucleoprotein complex export from nucleus (GO:0071431) | 22/34 | 5.28E-05 | 0.005 |
| RNA export from nucleus (GO:0006405) | 58/120 | 5.17E-05 | 0.005 |
| post-Golgi vesicle-mediated transport (GO:0006892) | 33/59 | 6.18E-05 | 0.006 |
| protein localization to plasma membrane (GO:0072659) | 62/131 | 6.40E-05 | 0.006 |
| protein dephosphorylation (GO:0006470) | 60/126 | 6.69E-05 | 0.007 |
| histone ubiquitination (GO:0016574) | 16/22 | 6.88E-05 | 0.007 |
| extracellular matrix organization (GO:0030198) | 99/230 | 7.31E-05 | 0.007 |
| Wnt signaling pathway, calcium modulating pathway (GO:0007223) | 23/37 | 8.99E-05 | 0.008 |
| protein localization to membrane (GO:0072657) | 73/161 | 8.85E-05 | 0.008 |
| Golgi organization (GO:0007030) | 58/122 | 9.29E-05 | 0.008 |
| intracellular protein transport (GO:0006886) | 141/348 | 9.58E-05 | 0.008 |
| positive regulation of protein modification process (GO:0031401) | 74/164 | 9.63E-05 | 0.008 |
| positive regulation of exocytosis (GO:0045921) | 22/35 | 1.01E-04 | 0.008 |
| phosphatidylinositol metabolic process (GO:0046488) | 54/113 | 1.35E-04 | 0.011 |
| regulation of coenzyme metabolic process (GO:0051196) | 24/40 | 1.41E-04 | 0.011 |
| protein deacylation (GO:0035601) | 13/17 | 1.50E-04 | 0.012 |
| histone monoubiquitination (GO:0010390) | 17/25 | 1.54E-04 | 0.012 |
| regulation of carbohydrate catabolic process (GO:0043470) | 23/38 | 1.61E-04 | 0.012 |
| protein modification by small protein removal (GO:0070646) | 109/262 | 1.71E-04 | 0.013 |
| positive regulation of cell morphogenesis involved in differentiation (GO:0010770) | 32/59 | 1.74E-04 | 0.013 |
| tRNA transport (GO:0051031) | 22/36 | 1.83E-04 | 0.013 |
| regulation of telomere maintenance via telomerase (GO:0032210) | 29/52 | 1.82E-04 | 0.013 |
| histone modification (GO:0016570) | 48/99 | 2.01E-04 | 0.014 |
| transcription-coupled nucleotide-excision repair (GO:0006283) | 38/74 | 2.07E-04 | 0.015 |
| organelle assembly (GO:0070925) | 149/377 | 2.47E-04 | 0.017 |
| regulation of autophagy (GO:0010506) | 87/204 | 2.81E-04 | 0.019 |
| protein targeting to vacuole (GO:0006623) | 18/28 | 2.89E-04 | 0.020 |
| viral life cycle (GO:0019058) | 51/108 | 2.93E-04 | 0.020 |
| response to calcium ion (GO:0051592) | 40/80 | 2.96E-04 | 0.020 |
| regulation of cellular catabolic process (GO:0031329) | 30/56 | 3.67E-04 | 0.024 |
| regulation of telomere maintenance via telomere lengthening (GO:1904356) | 13/18 | 3.86E-04 | 0.024 |
| global genome nucleotide-excision repair (GO:0070911) | 20/33 | 4.20E-04 | 0.024 |
| maturation of 5.8S rRNA from tricistronic rRNA transcript (SSU-rRNA, 5.8S rRNA, LSU-rRNA) (GO:0000466) | 14/20 | 3.81E-04 | 0.024 |
| 7-methylguanosine mRNA capping (GO:0006370) | 20/33 | 4.20E-04 | 0.024 |
| 7-methylguanosine RNA capping (GO:0009452) | 20/33 | 4.20E-04 | 0.024 |
| regulation of hydrolase activity (GO:0051336) | 40/81 | 4.11E-04 | 0.024 |
| regulation of myeloid cell differentiation (GO:0045637) | 34/66 | 4.05E-04 | 0.024 |
| negative regulation of intracellular signal transduction (GO:1902532) | 71/162 | 3.82E-04 | 0.024 |
| snRNA transcription from RNA polymerase II promoter (GO:0042795) | 36/71 | 4.13E-04 | 0.024 |
| snRNA transcription (GO:0009301) | 36/71 | 4.13E-04 | 0.024 |
| regulation of cellular component organization (GO:0051128) | 63/141 | 4.24E-04 | 0.024 |
| regulation of DNA metabolic process (GO:0051052) | 26/47 | 4.61E-04 | 0.025 |
| RNA secondary structure unwinding (GO:0010501) | 26/47 | 4.61E-04 | 0.025 |
| regulation of cellular component movement (GO:0051270) | 26/47 | 4.61E-04 | 0.025 |
| ATP-dependent chromatin remodeling (GO:0043044) | 19/31 | 4.78E-04 | 0.026 |
| positive regulation of cellular protein metabolic process (GO:0032270) | 44/92 | 0.001 | 0.028 |
| protein localization to cell periphery (GO:1990778) | 54/118 | 0.001 | 0.028 |
| regulation of cellular response to stress (GO:0080135) | 49/105 | 0.001 | 0.028 |
| regulation of vesicle fusion (GO:0031338) | 32/62 | 0.001 | 0.030 |
| protein deubiquitination (GO:0016579) | 105/258 | 0.001 | 0.030 |
| mitotic cell cycle phase transition (GO:0044772) | 92/222 | 0.001 | 0.031 |
| axon guidance (GO:0007411) | 69/159 | 0.001 | 0.032 |
| regulation of GTPase activity (GO:0043087) | 80/189 | 0.001 | 0.032 |
| protein modification process (GO:0036211) | 41/85 | 0.001 | 0.032 |
| protein transport (GO:0015031) | 129/327 | 0.001 | 0.033 |
| water-soluble vitamin metabolic process (GO:0006767) | 38/78 | 0.001 | 0.038 |
| transcription elongation from RNA polymerase II promoter (GO:0006368) | 45/96 | 0.001 | 0.038 |
| dephosphorylation (GO:0016311) | 56/125 | 0.001 | 0.038 |
| Golgi to endosome transport (GO:0006895) | 14/21 | 0.001 | 0.039 |
| regulation of phosphorylation (GO:0042325) | 54/120 | 0.001 | 0.040 |
| positive regulation of endothelial cell migration (GO:0010595) | 35/71 | 0.001 | 0.044 |
| regulation of ATP metabolic process (GO:1903578) | 24/44 | 0.001 | 0.045 |
| ncRNA processing (GO:0034470) | 93/228 | 0.001 | 0.048 |
| positive regulation of translation (GO:0045727) | 38/79 | 0.001 | 0.048 |

**Supplementary table S6: Delta cell proportions in islet preparations from control donors and those with type 1 or type 2 diabetes.** Table of median and interquartile range for the number of delta cells between patients with T1D and T2D compared to their respective controls.

| **Sample name** | ***p* value** | **Median** | **Interquartile range** | **Minimum** | **Maximum** |
| --- | --- | --- | --- | --- | --- |
| Control for T1D | 3.0 x 10^-6^ | 4.5 | 3 | 0 | 7 |
| T1D cases |  | 8.0 | 6 | 3 | 18 |
| Control for T2D | 2.2 x 10^-4^ | 5.0 | 3 | 0 | 9 |
| T2D cases |  | 8.5 | 6 | 3 | 15 |

**Supplementary table S7: Effects of rescue from treatment with either 25 mM high glucose or 0.5 mM tunicamycin by 72 hrs restoration to normal culture media on splicing factor, total gene expression and patterns of alternative splicing.** The p values for statistical significance as determined by STUDENT INDEPENDENT T-TEST, mean logged fold change in expression, standard errors of difference (SED) and the 95% confidence intervals are given below. Results meeting the Bonferroni corrected p value *p=<0.01* for 5 tests are presented in bold italic type*.* Bonferroni correction was not applied to the number of genes as these are á priori.

| **Control vs 24 hrs 25 mM glucose *p*=0.050** | | | | | | | | | | | | | | | | |
| --- | --- | --- | --- | --- | --- | --- | --- | --- | --- | --- | --- | --- | --- | --- | --- | --- |
| **Treatment 24 hrs 25 mM glucose** | | | | | | | | | | | **Treatment and rescue** | | | | | |
|  | P value | | Mean diff | | SED | | 95% CI | | | | | P value | Mean diff | SED | 95% CI | |
|  |  |  |  |  |  |  | Lower | | Upper | | |  |  |  | Lower | Upper |
| *ARX* | 0.620 | | -0.639 | | 0.119 | | -0.268 | | 0.396 | | | 0.996 | -0.0008 | 0.150 | -0.419 | 0.417 |
| ***FOXO1*** | ***0.010*** | | ***-0.164*** | | ***0.035*** | | ***-0.262*** | | ***-0.065*** | | | 0.157 | -0.050 | 0.288 | -0.130 | 0.029 |
| *GCG* | 0.269 | | -0.183 | | 0.143 | | -0.214 | | 0.581 | | | 0.832 | -0.006 | 0.026 | -0.805 | 0.068 |
| ***GCK*** | ***0.006*** | | ***-0.137*** | | ***0.026*** | | ***0.065*** | | ***0.210*** | | | 0.418 | 0.044 | 0.049 | -0.091 | 0.180 |
| *HHEX* | 0.205 | | -0.226 | | 0.149 | | -0.189 | | 0.641 | | | 0.611 | -0.074 | 0.134 | -0.447 | 0.299 |
| *INS* | 0.428 | | -0.071 | | 0.071 | | -0.235 | | 0.375 | | | 0.967 | -0.044 | 0.966 | -0.272 | 0.264 |
| ***MAFA*** | ***0.020*** | | ***-0.122*** | | ***0.032*** | | ***0.032*** | | ***0.213*** | | | 0.257 | 0.035 | 0.026 | -0.038 | 0.108 |
| *ND1* | 0.370 | | -0.255 | | 0.022 | | -0.069 | | 0.120 | | | 0.279 | 0.042 | 0.034 | -0.052 | 0.137 |
| *NKX2-2* | 0.585 | | 0.032 | | 0.055 | | -0.186 | | 0.121 | | | 0.424 | 0.345 | 0.247 | -0.439 | 0.506 |
| *NKX6-1* | 0.436 | | 0.050 | | 0.058 | | -0.211 | | 0.111 | | | 0.502 | -0.054 | 0.073 | -0.259 | 0.150 |
| ***PAX4*** | ***0.021*** | | ***-0.170*** | | ***0.046*** | | ***0.041*** | | ***0.298*** | | | 0.448 | 0.142 | 0.155 | -0.470 | 0.754 |
| *PAX6* | 0.300 | | -0.060 | | 0.045 | | -0.113 | | 0.234 | | | 0.481 | -0.048 | 0.062 | -0.222 | 0.125 |
| *PDX1* | 0.236 | | -0.072 | | 0.052 | | -0.072 | | 0.216 | | | 0.205 | -0.055 | 0.036 | -0.157 | 0.046 |
| *SST* | 0.955 | | -0.004 | | 0.084 | | -0.228 | | 0.238 | | | 0.715 | -0.030 | 0.078 | -0.249 | 0.188 |
| **Control Vs 24hrs 25 mM glucose *p*=0.050** | | | | | | | | | | | | | | | | |
| **Treatment 24 hrs 25 mM glucose** | | | | | | | | | | | **Treatment and rescue** | | | | | |
|  | | P value | | Mean diff | | SED | | 95% CI | | | | P  value | Mean diff | SED | 95% CI | |
| Gene | |  |  |  |  |  |  | Lower | | Upper | |  |  |  | Lower | Upper |
| ***INS*** | | ***0.004*** | | ***0.424*** | | ***0.072*** | | ***-0.623*** | | ***-0.226*** | | 0.476 | 0.058 | 0.077 | -0.124 | 0.241 |
| *NANOG* | | 0.511 | | 0.653 | | 0.923 | | 0.302 | | 0.171 | | 0.983 | -0.002 | 0.107 | -0.256 | 0.251 |
| ***PAX6*** | | ***0.003*** | | ***-1.278*** | | ***0.127*** | | ***0.539*** | | ***0.131*** | | 0.364 | -0.082 | 0.084 | -0.255 | 0.090 |
| *PTPN1* | | 0.353 | | 0.959 | | 0.096 | | 0.131 | | 0.323 | | 0.476 | 0.058 | 0.077 | -0.124 | 0.241 |
| ***POU5F1*** | | ***0.034*** | | ***0.373*** | | ***0.134*** | | ***0.039*** | | ***0.707*** | | 0.167 | -0.373 | 0.237 | -0.954 | 0.207 |

**Supplementary table S8: Effects of SH6 AKT pathway inhibition and treatment with 25 mM high glucose on splicing factor and patterns of alternative splicing.** The p values for statistical significance as determined by STUDENT INDEPENDENT T-TEST, mean logged fold change in expression, standard errors of difference (SED) and the 95% confidence intervals are given below. Statistically significant results are indicated in bold typeface**.**

| **SH6 and 25 mM glucose** | | | | | |
| --- | --- | --- | --- | --- | --- |
|  | | Sig. (2-tailed) | Std. Error Difference | 95% CI | |
|  | |  |  | Lower | Upper |
| 18S-Hs99999901_s1 | | 0.850 | 0.094 | -0.281 | 0.243 |
| AKAP17A-Hs00946624_m1 | | 0.621 | 0.104 | -0.232 | 0.343 |
| GUSB-Hs00939627_m1 | | 0.282 | 0.042 | -0.168 | 0.064 |
| HNRNPA0-Hs00246543_s1 | | 0.082 | 0.017 | -0.008 | 0.084 |
| HNRNPA1-Hs01656228_s1 | | 0.466 | 0.168 | -0.331 | 0.601 |
| HNRNPA2B1-Hs00242600_m1 | | 0.152 | 0.089 | -0.090 | 0.404 |
| HNRNPD-Hs01086914_g1 | | 0.543 | 0.038 | -0.131 | 0.080 |
| HNRNPH3-Hs01032113_g1 | | 0.945 | 0.056 | -0.159 | 0.151 |
| HNRNPK-Hs00829140_s1 | | 0.890 | 0.055 | -0.160 | 0.144 |
| HNRNPM-Hs00246018_m1 | | 0.638 | 0.056 | -0.185 | 0.128 |
| HNRNPUL2-BSCL2;HNRNPUL2-Hs00859848_m1 | | 0.428 | 0.062 | -0.227 | 0.117 |
| IDH3B-Hs00199382_m1 | | 0.485 | 0.022 | -0.044 | 0.078 |
| IMP3-Hs00251000_s1 | | 0.313 | 0.058 | -0.229 | 0.095 |
| LSM14A-Hs00385941_m1 | | 0.619 | 0.353 | -1.171 | 0.790 |
| LSM2-Hs01061967_g1 | | 0.385 | 0.048 | -0.179 | 0.086 |
| PNISR-Hs00369090_m1 | | 0.817 | 0.087 | -0.263 | 0.220 |
| PPIA-Hs04194521_s1 | | 0.509 | 0.093 | -0.190 | 0.324 |
| SF3B1-Hs00202782_m1 | | 0.759 | 0.068 | -0.211 | 0.167 |
| SRSF1-Hs00199471_m1 | | 0.556 | 0.026 | -0.055 | 0.088 |
| SRSF2-Hs00427515_g1 | | 0.699 | 0.047 | -0.111 | 0.150 |
| SRSF3-Hs00751507_s1 | | 0.610 | 0.051 | -0.112 | 0.168 |
| SRSF6-Hs00607200_g1 | | 0.665 | 0.053 | -0.122 | 0.172 |
| SRSF7-Hs00196708_m1 | | 0.729 | 0.084 | -0.202 | 0.265 |
| TRA2B-Hs00907493_m1 | | 0.922 | 0.044 | -0.117 | 0.126 |
| **SH6 treated cells exposed to 25 mM high glucose** | | | | | |
|  | Sig. (2-tailed) | | Std. Error Difference | 95% CI | |
|  |  |  |  | Lower | Upper |
| HPRT1 | 0.599 | | 0.373 | -1.250 | 0.824 |
| IDH3B | 0.753 | | 0.419 | -1.305 | 1.023 |
| INS | 0.866 | | 0.083 | -0.245 | 0.215 |
| NANOG | 0.161 | | 0.112 | -0.504 | 0.119 |
| PAX6 | 0.179 | | 0.030 | -0.035 | 0.132 |
| POU5F1 | 0.146 | | 0.104 | -0.101 | 0.477 |

**Supplementary table S9: Details of antibodies and experimental conditions.**

| **Antibody** | **Supplier** | **Cat #** | **Lot number** | **Species** | **Concentration used:** |
| --- | --- | --- | --- | --- | --- |
| INS | DAKO | 80564 | 10088287 | Guinea-pig polyclonal | 1/363 |
| GCG | Abcam | Ab10988 | GR260160-1 | Mouse monoclonal IgG1 | 1/2000 |
| SST | Abcam | Ab30788 | GR213035-1 | Rat monoclonal IgG2b | 1/200 |

**Supplemental table S10: Patient data for nPOD islet samples.**

| **Disease status** | **Case ID** | **Age (years)** | **Duration (years)** |
| --- | --- | --- | --- |
| T1D | SC107 | 18 | 5 |
| T1D | SC109 | 20 | 6 |
| T1D | E557 | 22 | 4 |
| T1D | SC112 | 22 | 9 |
| T1D | SC116 | 35 | 15 |
| T1D | nPOD6038-01PB | 37.5 | 20 |
| T1D | nPOD6086-02PB | 78 | 74 |
| Control | 12142 | 17 | - |
| Control | 146/66 | 18 | - |
| Control | PAN1 | 22 | - |
| Control | 329/72 | 24 | - |
| Control | 447/71 | 32 | - |
| Control | nPOD6095-06PB | 40 | - |
| Control | nPOD6012-4PT | 68 | - |
| T2D | 38/66 | 56 | 0.25 |
| T2D | 386/66 | unknown | unknown |
| T2D | 184/66 | 60 | unknown |
| T2D | 192/71 | 65 | unknown |
| T2D | 192/69 | 66 | unknown |
| Control | 110/96 | 58 | - |
| Control | 202/75 | 60 | - |
| Control | 224/66 | 67 | - |
| Control | 44/66 | 60 | - |
| control | 244/66 | 63 | - |

**Supplementary table S11: Assay details for gene involved in beta cell identity, function or fate, markers of hormone expression or cellular stress.**

| Gene Symbol | Assay ID |
| --- | --- |
| STK11 | Hs00176092_m1 |
| FOXO1 | Hs01054576_m1 |
| PAX6 | Hs00240871_m1 |
| GCK | Hs01564555_m1 |
| GUSB | Hs00939627_m1 |
| SLC16A1 | Hs01560299_m1 |
| MAFA | Hs01651425_s1 |
| NKX6-1 | Hs00232355_m1 |
| ARX | Hs00292465_m1 |
| PPIA | Hs04194521_s1 |
| LDHA | Hs01378790_g1 |
| NEUROD1 | Hs01922995_s1 |
| SOX9 | Hs01001343_g1 |
| PTF1A | Hs00603586_g1 |
| HPRT1 | Hs02800695_m1 |
| PDGFRA | Hs00998026_m1 |
| SYP | Hs00300531_m1 |
| VIM | Hs00958111_m1 |
| ONECUT1 | Hs00413554_m1 |
| NEUROG3 | Hs01875204_s1 |
| HES1 | Hs00172878_m1 |
| GCG | Hs01031536_m1 |
| PTPN1 | Hs00942477_m1 |
| POU5F1 | Hs04260367_gH |
| NKX2-2 | Hs00159616_m1 |
| INS | Hs00355773_m1 |
| HIF1A | Hs00153153_m1 |
| NANOG | Hs04399610_g1 |
| PAX4 | Hs00173014_m1 |
| SST | Hs00356144_m1 |
| ARNT | Hs01121918_m1 |
| MYCL | Hs00420495_m1 |
| PDX1 | Hs00236830_m1 |
| SLC2A2 | Hs01096908_m1 |
| DDIT3 | Hs00358796_g1 |
| MAFB | Hs00271378_s1 |
| HHEX | Hs00242160_m1 |

**Supplementary table S12: Assay Ids for splicing factors quantified in this study.**

| Gene symbol | Assay ID |
| --- | --- |
| AKAP17A | Hs00946624_m1 |
| HNRNPA0 | Hs00246543_s1 |
| HNRNPA1 | Hs01656228_s1 |
| HNRNPA2B1 | Hs00955384_m1 |
| HNRNPD | Hs01086912_m1 |
| HNRNPH3 | Hs01032113_g1 |
| HNRNPK | Hs03989611_gH |
| HNRNPM | Hs00246018_m1 |
| HNRNPUL2 | Hs01398726_g1 |
| IMP3 | Hs00251000_s1 |
| 18s rRNA | Hs99999901_s1 |
| LSM14A | Hs00385941_m1 |
| LSM2 | Hs01061967_g1 |
| PNISR | Hs00369090_m1 |
| SF3B1 | Hs00961640_g1 |
| SRSF1 | Hs00199471_m1 |
| SRSF2 | Hs00427515_g1 |
| SRSF3 | Hs00751507_s1 |
| SRSF6 | Hs00740177_g1 |
| SRSF7 | Hs01032695_m1 |
| TRA2B | Hs00190210_m1 |
| IDH3B | Hs00199382_m1 |
| PPIA | Hs04194521_s1 |
| GUSB | Hs00939627_m1 |

**Supplementary table S13: Sequences of isoform specific probes and primers used in this study.**

| Assay Name | Assay IDs | Forward Primer Seq. | Reverse Primer Seq. | Reporter 1 Sequence |
| --- | --- | --- | --- | --- |
| INS_EX1 | NM_001185097 | CCATCAAGCAGGTCTGTTCCAA | GGGCCATGGCAGAAGGA | CCTTTGCGTCAGATCACT |
| INS_EX1DEL | NM_000207 | GGCTTCTTCTACACACCCAAGAC | CCTCCAGGGCCAAGGG | CTGCAGGGCAGCCTG |
| INS_EX1LNG | NM_001185098 | GGACAGGCTGCATCAGAAGAG | GGGCCATGGCAGAAGGA | CCATCAAGCAGATCACTG |
| PTPN1_EX2DEL | NM_001278618.1 | AGTTCGAGCAGATCGACAAGTC | GCGTTGATATAGTCATTATCTTCTTGATGTAGT | CTATGGTCAACTGGTAAATG |
| NANOG1_EX4LNG | NM_024865.3 | TGGCCGAAGAATAGCAATGGT | CATCCCTGGTGGTAGGAAGAGTA | ACGCAGAAGGCCTCAGC |
| PAX6_EX5DEL | NM_001258462.1  NM_001258463.1  NM_001310158.1  NM_001310160.1  NM_001310161.1  NM_001604.5 | CGTGCGACATTTCCCGAATT | GTCTCGTAATACCTGCCCAGAATTT | ATCCGTTGGACACCTGC |
| NANOG_EX4TRU | NM_001297698 | TGGCCGAAGAATAGCAATGGT | GGTTCCCAGTCGGGTTCAC | ACGCAGGGATGCCTG |
| POU5F1_EX3 |  | GAAGAGGATCACCCTGGGATATACA | TGGCTGAATACCTTCCCAAATAGAAC | CAGGCCGATGTGGCTC |

**Supplementary Figure 1: Immunocytochemistry images of glucagon staining in EndoC βH1 cells**

Representative image shows absence of glucagon staining in EndoC βH1 cells treated with 25 mM glucose for 24 hours.


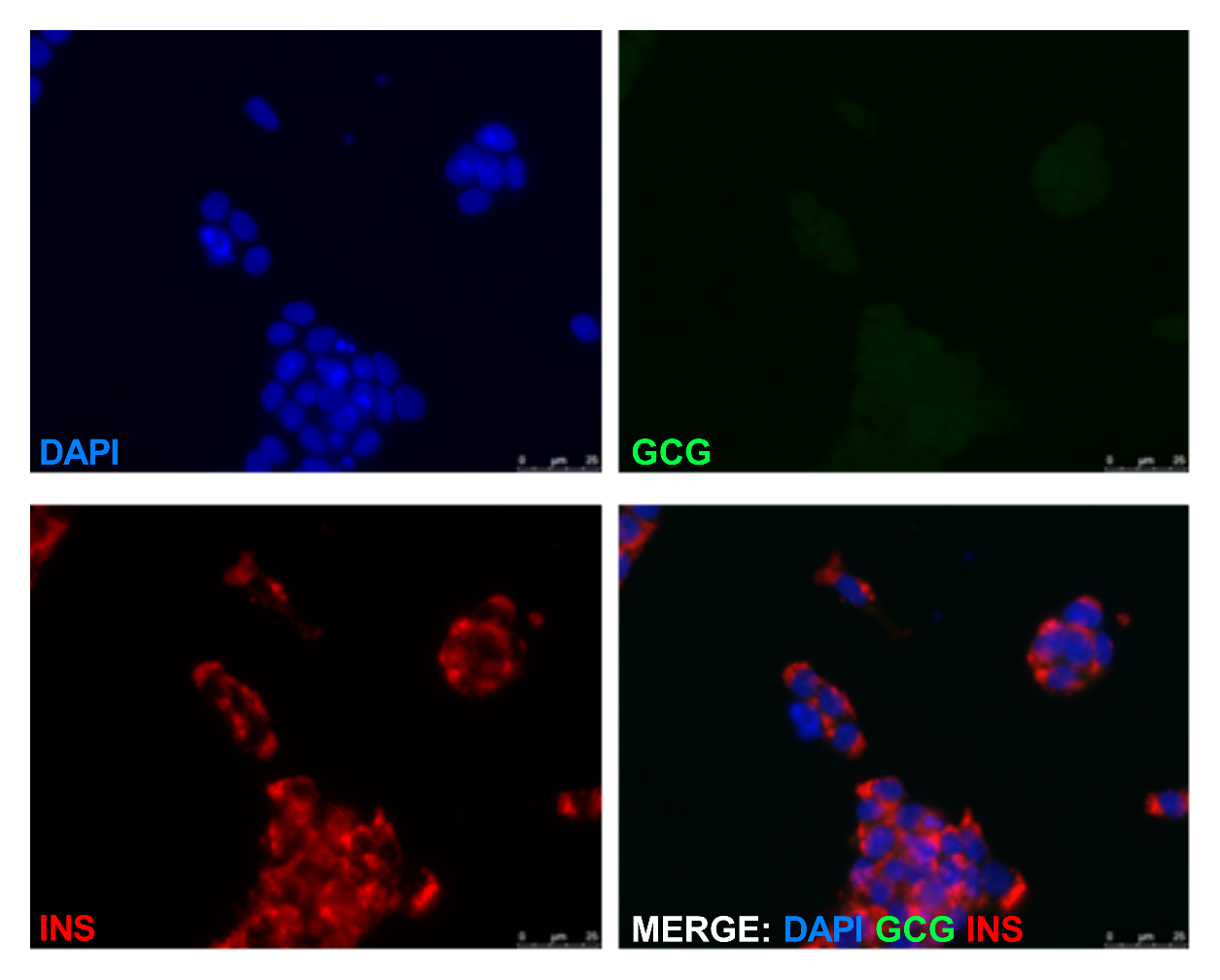

Supplement: Supplemental_tables_ddz094 [file supplemental_tables_ddz094.docx]
